# N-BLR, a primate-specific non-coding transcript leads to colorectal cancer invasion and migration

**DOI:** 10.1186/s13059-017-1224-0

**Published:** 2017-05-24

**Authors:** Isidore Rigoutsos, Sang Kil Lee, Su Youn Nam, Simone Anfossi, Barbara Pasculli, Martin Pichler, Yi Jing, Cristian Rodriguez-Aguayo, Aristeidis G. Telonis, Simona Rossi, Cristina Ivan, Tina Catela Ivkovic, Linda Fabris, Peter M. Clark, Hui Ling, Masayoshi Shimizu, Roxana S. Redis, Maitri Y. Shah, Xinna Zhang, Yoshinaga Okugawa, Eun Jung Jung, Aristotelis Tsirigos, Li Huang, Jana Ferdin, Roberta Gafà, Riccardo Spizzo, Milena S. Nicoloso, Anurag N. Paranjape, Maryam Shariati, Aida Tiron, Jen Jen Yeh, Raul Teruel-Montoya, Lianchun Xiao, Sonia A. Melo, David Menter, Zhi-Qin Jiang, Elsa R. Flores, Massimo Negrini, Ajay Goel, Menashe Bar-Eli, Sendurai A. Mani, Chang Gong Liu, Gabriel Lopez-Berestein, Ioana Berindan-Neagoe, Manel Esteller, Scott Kopetz, Giovanni Lanza, George A. Calin

**Affiliations:** 1 Computational Medicine Center, Sidney Kimmel Medical College at Thomas Jefferson University, Philadelphia, PA USA; 2 Department of Experimental Therapeutics, The University of Texas MD Anderson Cancer Center, Houston, TX USA; 3 Present address: Institute of Gastroenterology, Department of Internal Medicine, Yonsei University College of Medicine, Seoul, Korea; 4 Gastroenterology, Department of Internal Medicine, Kyungpook National University Medical School, Daegu, Korea; 5 Present address: Laboratory of Oncology, IRCCS Casa Sollievo della Sofferenza, San Giovanni Rotondo, FG Italy; 6 Present address: Division of Oncology, Medical University of Graz, Graz, Austria; 7 Present address: Institute of Oncology Research (IOR), Research Division of the Oncology Institute of Southern Switzerland (IOSI), Bellinzona, Switzerland; 8 Center for RNA interference and non-coding RNA, The University of Texas MD Anderson Cancer Center, Houston, TX USA; 9 Department of Molecular Medicine, Ruder Boskovic Institute, Zagreb, Croatia; 10 Department of Pathology and Laboratory Medicine, The Children’s Hospital of Philadelphia, Philadelphia, PA USA; 11 Department of Gynecologic Oncology, The University of Texas MD Anderson Cancer Center, Houston, TX USA; 12 Center for Gastrointestinal Research, and Center for Translational Genomics and Oncology, Baylor Scott & White Research Institute and Charles A. Sammons Cancer Center, Baylor University Medical Center, Dallas, TX USA; 13 Department of Surgery, School of Medicine, Gyeongsang National University, Jin-ju, South Korea; 14 Department of Pathology, NYU Langone Medical Center, New York, NY 10016 USA; 15 Department of Cancer Biology, The University of Texas MD Anderson Cancer Center, Houston, TX USA; 16 Present address: Institute of Biochemistry, Faculty of Medicine, University of Ljubljana, Ljubljana, Slovenia; 17 Department of Morphology, Surgery and Experimental Medicine, University of Ferrara, Ferrara, Italy; 18 Present address: CRO, National Cancer Institute, 33081 Aviano, Italy; 19 Department of Translational Molecular Pathology, The University of Texas MD Anderson Cancer Center, Houston, TX USA; 20 Department of Medicine, Nassau University Medical Center, 2201 Hempstead Tpke, East Meadow, NY 11554 USA; 21 Departments of Surgery and Pharmacology, UNC Lineberger Comprehensive Cancer Center, University of North Carolina at Chapel Hill, Chapel Hill, NC USA; 22 Present address: Centro Regional de Hemodonación, Universidad de Murcia, IMIB-Arrixaca, CIBEER (CB15/00055), Murcia, Spain; 23 Division of Quantitative Science, The University of Texas MD Anderson Cancer Center, Houston, TX USA; 24 i3S - Instituto de Investigação e Inovação em Saúde, Universidade do Porto, and Ipatimup - Institute of Pathology and Molecular Immunology of the University of Porto, 4200 Porto, Portugal; 25 Department of Pathology, Faculty of Medicine of Porto University, 4200-319 Porto, Portugal; 26 Department of Gastrointestinal Medical Oncology, The University of Texas MD Anderson Cancer Center, Houston, TX USA; 27 Department of Molecular Oncology, Moffitt Cancer Center, Tampa, FL USA; 28 Research Center for Functional Genomics, Biomedicine and Translational Medicine, Medfuture, Cluj-Napoca Romania; 29 Research Center for Advanced Medicine - University of Medicine and Pharmacy “Iuliu Haţieganu”, Cluj-Napoca, Romania; 30 Department of Functional Genomics, Proteomics and Experimental Pathology- The Oncology Institute “ Prof Dr. Ion Chiricuta, Cluj-Napoca, Romania; 31 Cancer Epigenetics and Biology Program, Bellvitge Biomedical Research Institute (IDIBELL), L’Hospitalet, Barcelona, Catalonia Spain; 32 Physiological Sciences Department, School of Medicine and Health Sciences, University of Barcelona (UB), Barcelona, Catalonia Spain; 33 Institucio Catalana de Recerca i Estudis Avançats (ICREA), Barcelona, Catalonia Spain; 34 Department of Medical Sciences, University of Ferrara, Ferrara, Italy; 35 Present address: ProQR Therapeutics, Leiden, Netherlands; 36 Present address: National Cancer Institute, Bethesda, MD USA

**Keywords:** Non-coding RNA, Pyknons, Transcription, ncRNA, lncRNA, N-BLR, EMT, CRC, CLL

## Abstract

**Background:**

Non-coding RNAs have been drawing increasing attention in recent years as functional data suggest that they play important roles in key cellular processes. N-BLR is a primate-specific long non-coding RNA that modulates the epithelial-to-mesenchymal transition, facilitates cell migration, and increases colorectal cancer invasion.

**Results:**

We performed multivariate analyses of data from two independent cohorts of colorectal cancer patients and show that the abundance of N-BLR is associated with tumor stage, invasion potential, and overall patient survival. Through *in vitro* and *in vivo* experiments we found that N-BLR facilitates migration primarily via crosstalk with E-cadherin and ZEB1. We showed that this crosstalk is mediated by a pyknon, a short ~20 nucleotide-long DNA motif contained in the N-BLR transcript and is targeted by members of the miR-200 family. In light of these findings, we used a microarray to investigate the expression patterns of other pyknon-containing genomic loci. We found multiple such loci that are differentially transcribed between healthy and diseased tissues in colorectal cancer and chronic lymphocytic leukemia. Moreover, we identified several new loci whose expression correlates with the colorectal cancer patients’ overall survival.

**Conclusions:**

The primate-specific N-BLR is a novel molecular contributor to the complex mechanisms that underlie metastasis in colorectal cancer and a potential novel biomarker for this disease. The presence of a functional pyknon within N-BLR and the related finding that many more pyknon-containing genomic loci in the human genome exhibit tissue-specific and disease-specific expression suggests the possibility of an alternative class of biomarkers and therapeutic targets that are primate-specific.

**Electronic supplementary material:**

The online version of this article (doi:10.1186/s13059-017-1224-0) contains supplementary material, which is available to authorized users.

## Background

Novel experimental methods and recent technological advances have established that in addition to the protein-coding regions, significant parts of the human and other genomes give rise to short and long non-coding RNAs (ncRNAs) [1]. In terms of diversity, ncRNAs handily outnumber protein-coding transcripts complicating functional investigations [2]. Indeed, many classes of experimentally identified ncRNAs have been reported in the literature, including microRNAs (miRNAs), Piwi-interacting RNAs (piRNAs), long intergenic non-coding RNAs (lincRNAs), transcription initiation RNAs (tiRNAs), miRNA-offset RNAs (moRNAs), sno-derived RNAs (sdRNAs), transfer RNA (tRNA) fragments [3–5] or long enhancer ncRNAs (eRNAs) [6], and others. However, the full repertoire of ncRNAs and their functional involvement in the regulation of cellular processes and, by extension, in the onset and progression of human disorders remains largely unknown [6, 7].

The best-studied ncRNA transcripts are miRNAs. Between 19 and 23 nucleotides (nt) in length, miRNAs bind their target messenger RNAs (mRNAs) in a sequence-dependent manner thereby regulating their targets’ levels [8, 9]. During the past 15 years, miRNAs have been implicated in many disease settings including cancers [10] and also found to act as mediators of molecular interactions that obviate direct molecular contact [11].

Long non-coding RNA (lncRNAs) burst onto the scene much later than miRNAs and many of them are currently known in the public domain [7, 12]. Although the full spectrum of lncRNAs remains unclear, several have been shown to be important in diverse contexts such as chromatin modification and remodeling [13, 14], X chromosome inactivation [15–17], lineage-specific transcriptional silencing [18], regulation of mRNA export [19], activation of a growth-control gene program [20] or of homeobox genes [21], and lineage-specific silencing [22]. LncRNAs have also been linked to human conditions such as brachydactyly [23] and Prader–Willi syndrome [24], and to cancers such as melanoma [25], colon [26, 27], and prostate cancer [28].

Pyknons (“peak-non-s”) are a class of short DNA sequence motifs that were initially identified computationally in the human genome using an unsupervised motif discovery process [29, 30]. A core property of pyknons is that they have multiple exact copies in the intergenic and intronic regions of the genome and in at least one mRNA. It is worth noting that nearly all mRNAs contain one or more pyknons, suggesting the possibility of long-distance interactions without direct molecular contact [11, 31]. A comparison of human and mouse pyknon sequences showed that pyknons are not syntenic, their sequences are organism-specific and not conserved across genomes, and their intronic copies are over-represented in the same groups of protein-coding genes in human and mouse [30, 32, 33]. The pyknons’ numerous genomic copies raise intriguing prospects for regulatory control [32], something that received experimental support recently [33, 34]. Pyknons have also been reported in plants where they are found to have the same properties as their animal counterparts [35]. It has also been reported that the DNA methyltransferase DNMT1 binds RNAs at pyknon loci and that the corresponding regions are hypo-methylated [36].

In what follows, we describe our discovery and characterization of a novel pyknon-containing lncRNA that we termed N-BLR (pronounced: eNaBLeR). We examine N-BLR’s expression in normal colon and colorectal cancer (CRC) and elucidate its role in shaping the epithelial-to-mesenchymal transition (EMT) and in enabling migration and invasion. We further examine, *in vitro* and *in vivo*, the molecular mechanism underlying the phenotype induced by N-BLR and discuss how a pyknon motif in N-BLR’s sequence can modulate N-BLR’s abundance in CRC. With the help of a microarray panel that we custom-designed, we investigate the transcription patterns of an additional 2500+ human genome loci that contain pyknons and find that many of these sequences are transcribed and associated, in various combinations, with the normal or pathological states of several tissues.

## Results

### Transcription of pyknon-containing segments of DNA correlates with clinical parameters and the overall survival of CRC patients

Initially, we sought to examine whether pyknons represent “passive” DNA motifs (e.g. genomic locations to which transcription factors could bind) or “active” sources of novel transcripts. We reasoned that regions associated with loss of heterozygosity (LOH) and “fragile sites” might represent good starting points, given that both have been shown to contain an excess of functionally relevant regulatory sequences [37]. To this end, we designed an exploratory collection of 11 quantitative real-time polymerase chain reaction (qRT-PCR) assays for pyknon instances in these regions; we denoted these 11 regions as pyk-reg-14, pyk-reg-17, pyk-reg-26, pyk-reg-27, pyk-reg-40, pyk-reg-41, pyk-reg-42, pyk-reg-43, pyk-reg-44, pyk-reg-83, and pyk-reg-90, respectively (Additional file 1: Table S1 and Additional file 2: Table S2). Owing to our long-standing interest in CRC [26], we used the 11 assays to explore the possibility of transcription across several microsatellite stable (MSS) and microsatellite instable high (MSI-H) cell lines: Colo320, SW480, HCT116, LS174, HT-29, Colo205, and SW620. We observed transcription from all 11 genomic pyknon locations with expression levels that varied among the seven cell lines (Additional file 3: Figure S1).

**Fig. 1:**
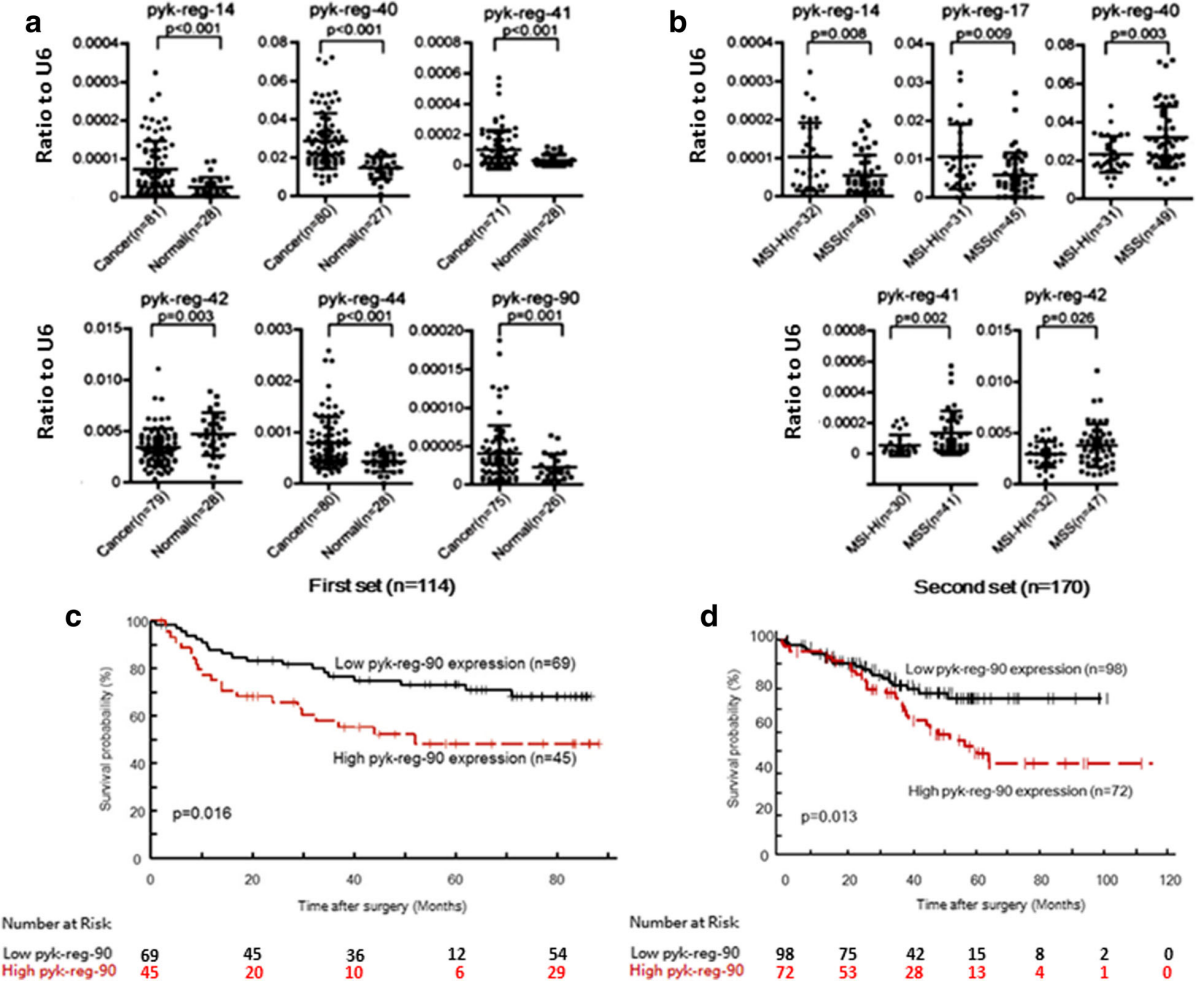
Pyknon loci expression in CRC samples by qRT-PCR. **a** Expression and distribution of pyknon-containing regions were analyzed between CRC and paired normal samples (first set, see Additional file 4: Table S3) by qRT-PCR. **b** Expression and distribution of pyknon-regions were analyzed between MSS and MSI-H CRC by qRT-PCR. The number of samples with measurable expression values (under Ct of 35) is presented in parentheses. The numbers of cancer and normal samples in some cases differ from one another because patients with no expression values for the U6 or for pyknon regions were excluded. Two-sided t-test was used to evaluate differences between two groups. Y-axis values represent ratio of each pyknon region to U6: ratios were calculated with the 2^–ΔCt^ method using U6 levels for normalization. **c**, **d** Kaplan–Meier curves reveal a poor clinical prognosis for patients with high pyk-reg-90 expression in both cohorts (the first set had n = 114 and the second set n = 170 patients); the association was statistically significant with *p* = 0.016 and *p* = 0.013 for each set, respectively (log-rank test). The high/low pyk-reg-90 expression was determined according to a cutoff value corresponding to the mean value of all patients

Spurred by these findings, we expanded our investigations to tissue samples from human normal colon and CRC and evaluated a first set of 81 tumor samples (randomly selected among the 127 samples of the first CRC patient cohort; see Additional file 4: Table S3) and 28 adjacent normal mucosa samples of Caucasian ancestry. In this group of 81 tumor and 28 normal samples, we found significant differences in CRC compared with normal tissue in the abundance of pyk-reg-14, pyk-reg-40, pyk-reg-41, pyk-reg-42, pyk-reg-44, and pyk-reg-90 (Fig. 1a). Additionally, we detected significant differences between MSS and MSI-H CRCs for pyk-reg-14, pyk-reg-17, pyk-reg-40, pyk-reg-41, and pyk-reg-42 (Fig. 1b). One of the loci in particular, pyk-reg-90, stood apart from the rest. Both univariate and multivariate logistic regression analysis performed on this first CRC patient cohort revealed a significant correlation between high levels of pyk-reg-90 and high tumor stage (stages III and IV) with an odds ratio of 3.72 (*p* = 0.001) and 3.49 (*p* = 0.011), respectively (Additional file 5: Table S4a). Moreover, we found that high levels of pyk-reg-90 were also associated with poor overall survival (OS) (*p* = 0.016, Fig. 1c and Additional file 6: Table S4b). When we analyzed a second independent cohort of 170 CRC patients (Additional file 7: Table S5), we observed a similar correlation between high levels of pyk-reg-90 and poor survival (Fig. 1d), high tumor stage (Additional file 8: Table S6a), and OS (Additional file 9: Table S6b). We also examined a third independent cohort (Additional file 10: Table S7) consisting exclusively of 21 metastatic CRC patient-derived xenografts and found pyk-reg-90 to be present in 15 of the 21 samples (*p* = 0.026 when compared with the probability of observing this frequency accidentally; Additional file 3: Figure S2).

### Cloning of the N-BLR lncRNA and expression by in situ hybridization

The pyk-reg-90 instance of interest is located in the 3p21.1–3p21.2 region on the forward strand of chromosome 3. By performing GeneRacer cloning, we were able to clone N-BLR (a novel pyk-reg-90-containing lncRNA) in HCT116 and Colo320 cells as well as normal colon and establish its identity as an 844-nt mono-exonic transcript (Additional file 3: Figure S3A and C left), without any other species homolog except a primate predicted ncRNA (Additional file 3: Figure S3E). Subsequent Sanger sequencing carried out independently at two different locations (Calin laboratory and Rigoutsos laboratory) confirmed that the same exact sequence, in terms of nucleotide content and length, was cloned from all three sources. N-BLR is transcribed from a contiguous block of genomic DNA (i.e. it is not spliced) on the forward strand of chromosome 3, in the intergenic space between the *POC1A* locus and the *ALAS1* locus. *POC1A* is located on the reverse strand of chromosome 3, i.e. on the strand opposite from N-BLR, and its transcription start site (TSS) is approximately 1.2 kb upstream from N-BLR (Additional file 3: Figure S3B). *ALAS1* is on the same strand as N-BLR but more than 40 kb downstream from it. Notably, N-BLR does not harbor any long open reading frame: this suggests lack of protein-coding potential, which we were able to verify by using an *in vitro* transcription-translation assay (Additional file 3: Figure S3C right). This was also corroborated independently using two software tools that evaluate a transcript’s protein coding potential (Additional file 3: Figure S3D). Moreover, we verified that in the genomic neighborhood of pyk-reg-90 transcription preferentially favors the forward strand, i.e., it is sense to the N-BLR transcript (Additional file 3: Figure S4A). We also searched for additional transcripts using primers targeting flanking regions at 1 kb, 2.5 kb, and 5 kb beyond N-BLR, on both the forward and the reverse strands: except for the region immediately 5′ to N-BLR, where the *POC1A* gene is located, all other qRT-PCR-identified transcripts were expressed at levels lower than N-BLR’s (Additional file 3: Figure S4B).

**Fig. 2:**
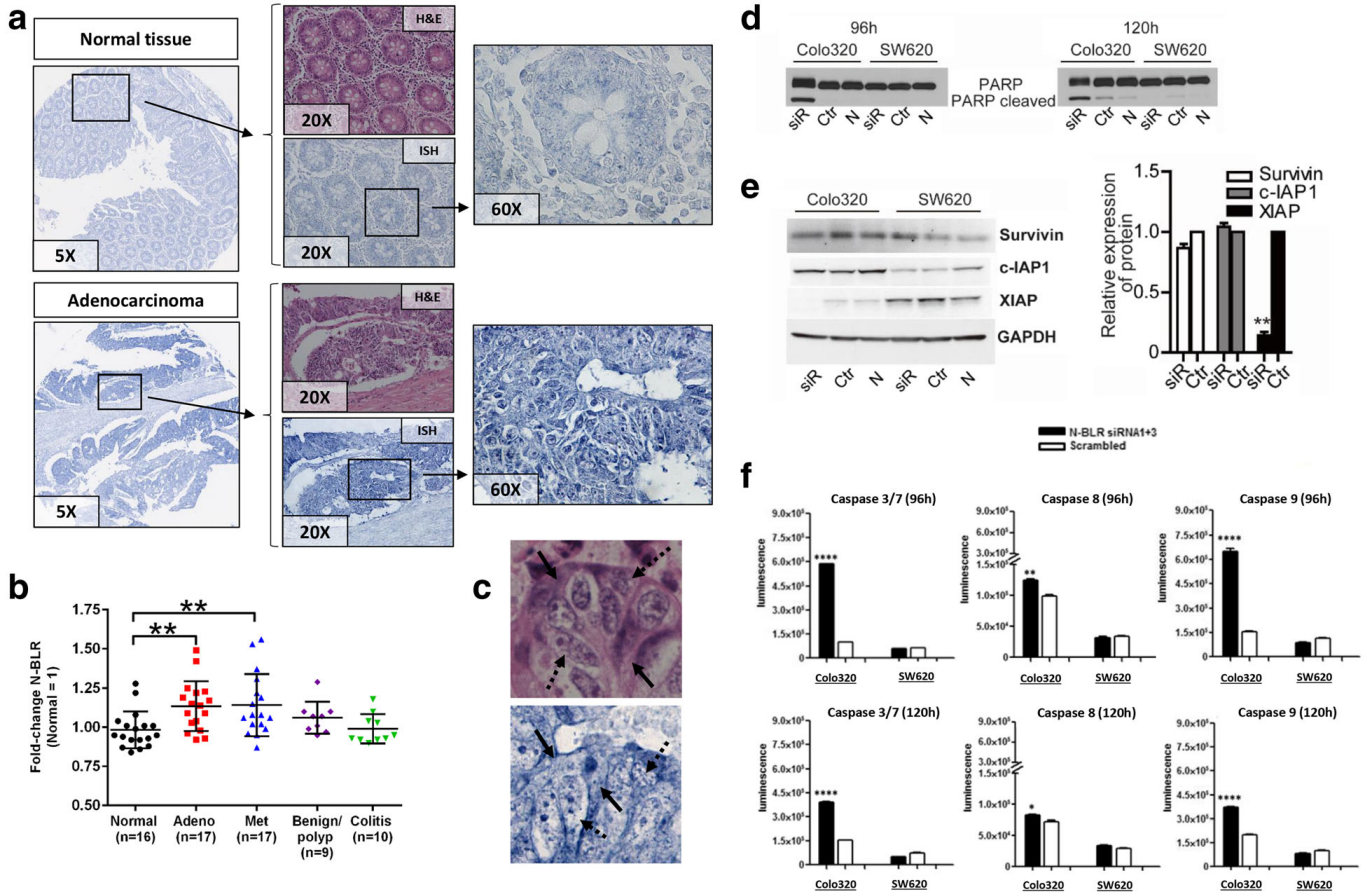
Properties of N-BLR. **a** ISH of the tissue microarray (described in Additional file 3: Figure S5) shows differential expression of N-BLR in colon cancer (Adenocarcinoma) and normal colon (Normal tissue). Hematoxylin and eosin (H&E) staining of matched tissues was added to distinguish tissue morphology. Increasing magnifications were provide to evaluate the distribution of N-BLR in the nucleus and in the cytoplasm of cells (5X, 20X, and 60X). **b** Image analysis of ISH was conducted to measure the expression levels of N-BLR in the different tissues. Adenocarcinoma and metastatic colon cancer tissues expressed higher levels of N-BLR compared with normal colon tissue. There were not significant differences between normal tissue and benign/polyp and colitis tissues. **c** ISH data on cytoplasmic/nuclear localization of N-BLR. The full *arrows* point to cytoplasm and the dashed arrows to nucleus. Those two cellular compartments were identified using H&E staining. The H&E staining and ISH for N-BLR were done on serial sections; therefore, perfect overlapping of tissue morphology did not occur between the two images that show the same tissue area. **d** PARP-1 expression following transfection of Colo320 and SW620 cells with siRNAs (N-BLR siRNA1 + 3 pool) against N-BLR. Profiling was carried out at 96 and 120 h of siRNA transfection. **e** *left* Expression of survivin, c-IAP-1, XIAP after 96 h following transfection of Colo320 and SW620 cells with siRNAs (N-BLR siRNA1 + 3 pool) against N-BLR. *right* Quantification of survivin, c-IAP-1, XIAP in Colo320 cells. **f** Activity of Caspase 3/7, Caspase 8, and Caspase 9 following transfection of Colo320 and SW620 cells with siRNAs (N-BLR siRNA1 + 3 pool) against N-BLR. Profiling was carried out after 96 and 120 h (siR = N-BLR siRNA 1 + 3 pool; Ctr = scramble control siRNA; N = lipofectamine only; GAPDH was used as loading control). (Student’s t-test; *p < 0.05; **p < 0.01; ***p < 0.001; and ****p < 0.0001)

We also used custom-designed LNA probes against N-BLR to carry out in situ hybridization (ISH) on a large commercially obtained tissue microarray (TMA) containing normal tissue, adenocarcinoma, metastatic, benign/polyp, and colitis samples from colon (Additional file 3: Figure S5A). As can be seen in Fig. 2a and b and Additional file 3: Figure S5B, we observed significantly higher expression levels of N-BLR in cancer (primary adenocarcinoma and metastatic tumors) compared with normal colon tissue, which is concordant with our qRT-PCR findings on N-BLR expression levels (Fig. 1a). Moreover, we did not measure significant differences comparing colitis and benign/polyp lesions with normal tissue, suggesting that overexpression of N-BLR occurs specifically in epithelial malignant cells and not in the tumor microenvironment or in premalignant or inflammatory lesions. ISH images from cancer tissue at high magnification also indicated that the N-BLR transcript was present in both the nucleus and the cytoplasm, with a predominance in the latter (Fig. 2c and Additional file 3: Figure S5C). The same cellular distribution of N-BLR was also observed in HCT116 and SW480 CRC cell lines, with SW480 exhibiting the highest cytoplasm/nucleus N-BLR ratio (Additional file 3: Figure S5D).

### N-BLR is a novel regulator of the apoptotic pathway

To address the function of N-BLR in CRC cells, we silenced its expression in Colo320 and SW620. Colo320 cells have high endogenous levels of N-BLR, whereas SW620 cells express it at minimal levels (Additional file 3: Figure S1); therefore, we used SW620 cells as negative control to exclude off-target effects of the silencing approach. We designed four siRNAs against N-BLR (labeled N-BLR siRNA1, N-BLR siRNA2, N-BLR siRNA3, and N-BLR siRNA4) and tested their ability to target N-BLR. SiRNA1 and siRNA3 were the most effective against N-BLR. Therefore, we combined them in a siRNA pool (N-BLR siRNA1 + 3 pool) that could reduce N-BLR levels to less than 30%, in a dose-dependent manner (Additional file 3: Figure S6A). Following a titration from 50 nM to 300 nM (Additional file 3: Figure S6B), we selected the concentration of 100 nM for subsequent experiments, in accordance to our N-BLR knockdown results and previously reported studies showing efficient lncRNAs knockdown at this concentration [38–41]. Following transfection with the siRNA pool, N-BLR levels began decreasing at 48 h and they remained low at a second measurement at 96 h (Additional file 3: Figure S6C). Cell counts of Colo320, but not of SW620 (data not shown), were significantly decreased at 96 h following treatment with either N-BLR siRNA1 or siRNA3, or the N-BLR siRNA1 + 3 pool (Additional file 3: Figure S6D).

Apoptotic profiling of Colo320 cells following siRNA treatment with the N-BLR siRNA1 + 3 pool revealed significantly increased levels of cleaved PARP-1, a substrate for activated cell-death proteases Caspases-3 and Caspase-7 compared with scrambled control siRNA (Fig. 2d). Expression of the X-linked inhibitor of apoptosis (XIAP), an inhibitor of Caspase-3 and Caspase-7, was abolished in Colo320 cells treated with N-BLR siRNA1 + 3 pool (*p* < 0.001), but not in SW620 “control” cells (Fig. 2e). We also confirmed the decreased mRNA levels of XIAP in Colo320 cells after 96 h transfection with N-BLR siRNA1 + 3 pool (Additional file 3: Figure S6E left). We did not observe any significant variations in the levels of the other two IAP family members, namely survivin and c-IAP1. The levels of activity of both initiator Caspase-8/9 and effector Caspase-3/7 were significantly increased in Colo320 cells, but not in SW620 “control” cells after N-BLR siRNA1 + 3 pool transfection (Fig. 2f). The higher apoptosis in Colo320, but not SW620, was further confirmed by cell cycle analyses (Additional file 3: Figure S6F and G).

N-BLR’s levels were profiled in additional colon cancer cell lines (Additional file 3: Figure S7A). In addition, the effect of siRNA-mediated N-BLR knockdown on apoptosis was assessed in two additional cell lines, SW480 and RKO. We found that downregulation of N-BLR was significantly associated with increased apoptosis at 96 h and 120 h following N-BLR siRNA1 + 3 pool transfection (Additional file 3: Figure S7B). Conversely, the stable overexpression of N-BLR in two independent cell lines, SW620 and HCT116, was associated with a decreased apoptosis (Additional file 3: Figure S7C), confirming that the apoptotic phenotype identified in Colo320 MSS cells can be reproduced in multiple colon cancer models of both MSS and MSI phenotype by using both upregulation and downregulation of N-BLR expression.

### N-BLR promotes invasion and migration

**Fig. 3:**
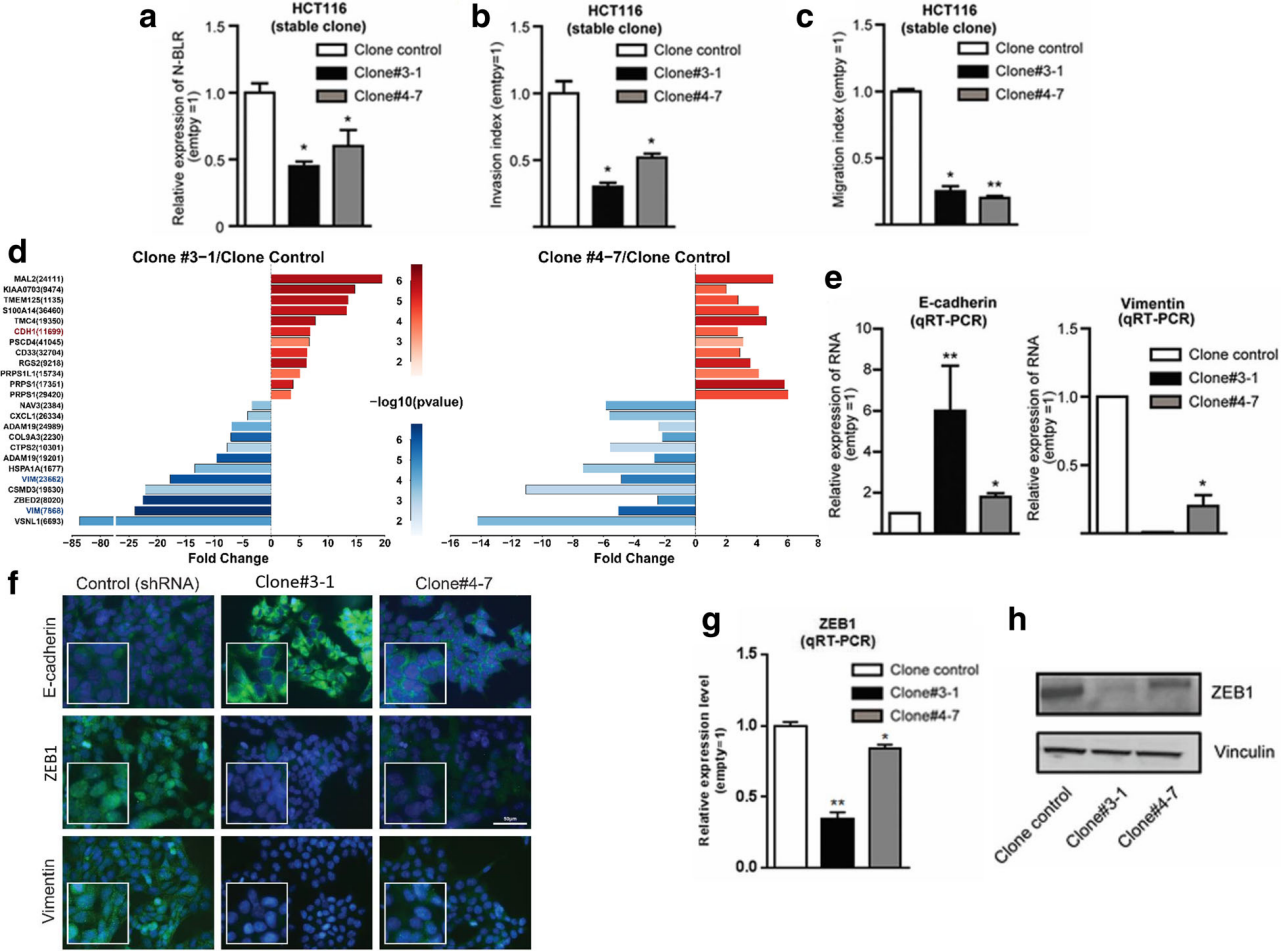
The effect of N-BLR knockdown on invasion by specific siRNAs. **a** N-BLR abundance is decreased in stably silenced clones. **b** Invasion assays at 36 h show significant reduction of stably silenced N-BLR invading cells. **c** Migration assay at 24 h identified also significant reduction in migration of stably silenced N-BLR clones. **d** The 12 most significantly differentially expressed genes for both upregulated and downregulated genes. The data originated from 44 K Agilent microarray where HCT116 stable shRNA N-BLR clones #3-1 and #4-7 were compared with HCT116 empty vector control clone. The probes recognizing E-cadherin and vimentin are in *red* and *blue*, respectively. **e** Confirmation of microarray data by real time PCR shows that E-cadherin is increased and vimentin is markedly decreased in stably silenced clones (#3-1 and #4-7). **f** E-cadherin, vimentin, and ZEB1 were identified *in vitro* by immunofluorescence with specific antibodies. Immunofluorescence signal of E-cadherin (*green color*) was markedly increased in both clones. The ZEB1 signal was present in cells with empty vector (*green color*) but not in clones #3-1 and #4-7. *Blue color* indicate nuclei. Single *green*, *blue*, and merged channel images of ZEB1 are reported in Additional file 3: Figure S9B. **g** ZEB1 mRNA downregulation in HCT116 stable shRNA N-BLR clones #3-1 and #4-7 compared with control HCT116 empty vector clone. **h** Western blotting for E-cadherin and ZEB1 measured in the same clones; vinculin was used as loading control. (Student’s t-test; *p < 0.05; **p < 0.01; ***p < 0.001; ****p < 0.0001)

To further investigate the effect of N-BLR downregulation during tumorigenesis, we evaluated the ability to modulate the migratory and invasive properties of cancer cells, which support the dissemination from the primary tumor and the metastatic spread to distant organs. To this end, we selected the HCT116 cells because their endogenous N-BLR levels are relatively high among the panel of colon cancer cell lines examined. Moreover, HCT116 cells exhibit greater adhesive capabilities compared with Colo320 cultures that have both adherent and non-adherent populations. We established HCT116 clones (Clone #3-1 and Clone #4-7) that stably expressed N-BLR shRNA and had significantly reduced levels of N-BLR (Fig. 3a). With regard to their motility ability, both clones showed a concomitant decrease by more than 50% in their invasion ability (Fig. 3b) and more than 60% reduction in their migration ability (Fig. 3c) compared with HCT116 cells transfected with empty vector (clone control). The ability of N-BLR to affect the motility of tumor cells was also evaluated by transiently overexpressing N-BLR in RKO cells that have relatively low levels of endogenous N-BLR. The transient increase in the levels of N-BLR resulted in enhanced capability of RKO cells to migrate and invade (Additional file 3: Figure S8A–C).

To understand the molecular basis regulating the mobility ability, we used microarrays to evaluate the effect of N-BLR on the expression of protein-coding genes in the two HCT116 clones (Clone #3-1, Clone #4-7). We found E-cadherin (CDH1) to be among the most upregulated and vimentin (VIM) among the most downregulated genes (Fig. 3d). This is notable since CDH1 and vimentin are involved in the EMT and cell motility control in human colon carcinoma [42]. We confirmed these findings by qRT-PCR (Fig. 3e) and immunofluorescence (Fig. 3f and Additional file 3: Figure S9A–C). Furthermore, the downregulation of vimentin, associated with N-BLR knockdown, was accompanied by downregulation of ZEB1 (Fig. 3f–h). ZEB1 is a known transcription factor that acts as negative regulator of E-cadherin and positive regulator of a number of other mesenchymal markers, including vimentin, N-cadherin, and matrix metalloproteinases; thereby, ZEB1 facilitates cell migration, invasion, and the eventual metastasis to distant organs [43].

### N-BLR and endogenous miRNAs are reciprocally regulated

**Fig. 4:**
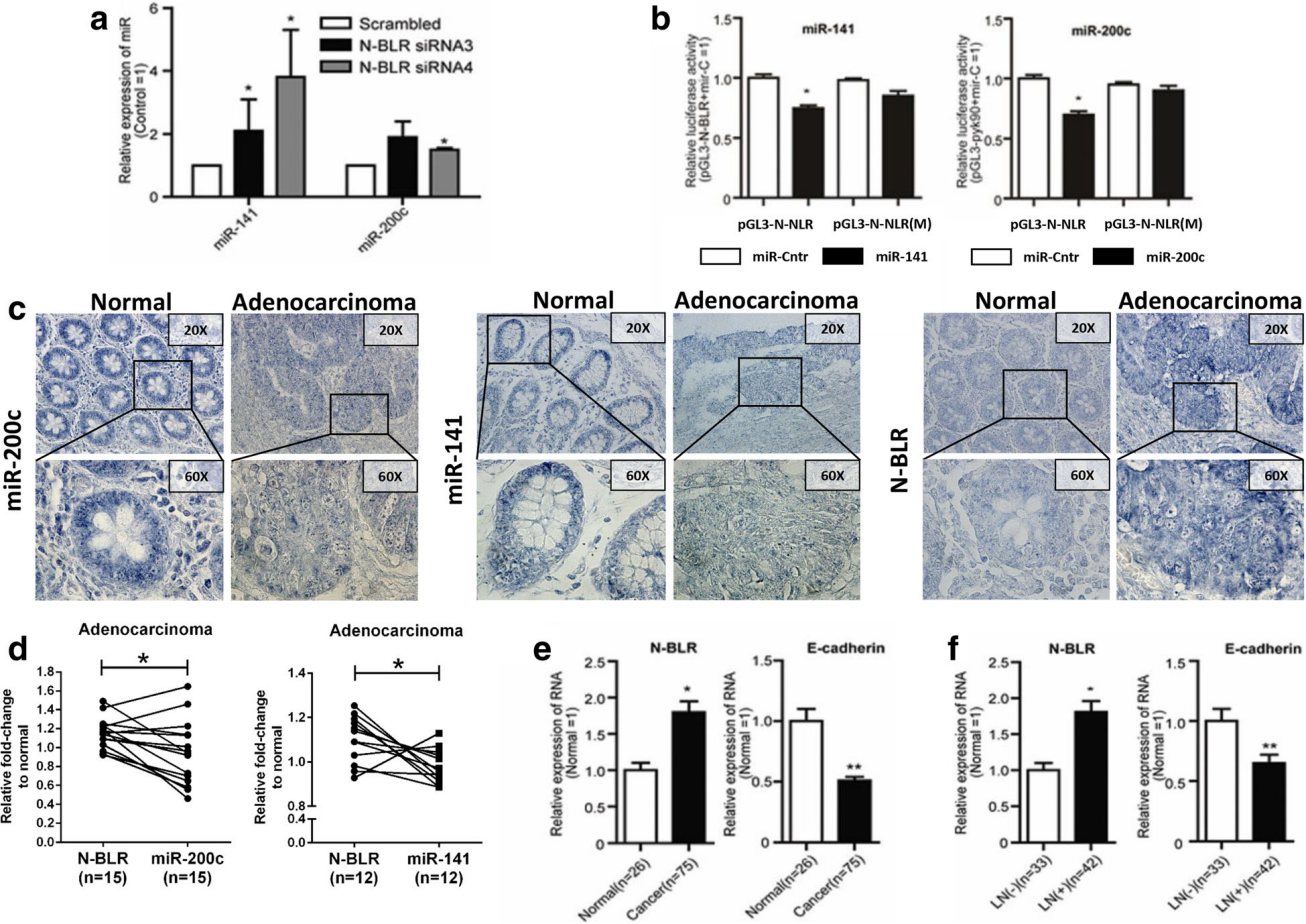
Interaction between N-BLR and miR-200 family members. **a** The effect of transient transfection of N-BLR siRNA3 and siRNA4 on the miR-200 family in Colo320 cells. MiR-141-3p and miR-200c-3p were increased in both N-BLR siRNAs transfected cells compared with scramble control. **b** A luciferase vector including the full N-BLR sequence (pGL3-N-BLR) as well as vectors that were mutated separately at the interaction sites of either miR-141-3p or miR-200c-3p [pGL3-N-BLR(M)] were constructed. Luciferase activity is decreased only when miR-141-3p and miR-200c-3p are co-transfected with the WT construct but not when a mutated vector is used. **c** Most representative images from ISH of tissue microarray showed lower levels of both miR-141-3p and miR-200c-3p in adenocarcinoma tissue compared with normal tissue, whereas an inverse pattern was found for N-BLR levels. **d** Image analysis were performed to evaluate the association between the levels of miR-141-3p and miR-200c-3p and those of N-BLR. The quantification was performed in a pair-matched fashion, so that the levels of the three targets were quantified on the same tissue spot of the microarray. **e** N-BLR and E-cadherin expression in tumor and normal samples: N-BLR was increased and E-cadherin was decreased in CRC when compared to normal colon. **f** The same is true when CRC with lymph node invasion (LN+) were compared with cases without lymph node involvement (LN–). *Asterisks* mark cases with statistically significant difference compared with scrambled. (Student’s t-test; *p < 0.05; **p < 0.01; ***p < 0.001; ****p < 0.0001)

In light of N-BLR’s presence also in the cytoplasm, we next examined the possibility that its transcript can interact with mature miRNAs. It was previously reported that the miR-200 family is involved in the regulation of EMT through a negative feedback loop with the ZEB1 and ZEB2 transcription factors [44]. Therefore, we further investigated the possibility of an interaction between N-BLR and the miR-200 family. To prioritize among the miR-200 family’s members, we used the rna22 algorithm [45] to predict putative miRNA targets: miR-141-3p and miR-200c-3p were predicted to target N-BLR (Additional file 3: Figure S10A). Interestingly, when we transiently knocked down N-BLR in Colo320 cells, we noted a concomitant increase in the levels of miR-141-3p and miR-200c-3p (Fig. 4a). We observed the same pattern in HCT116 shRNA N-BLR clones (#3-1 and #4-7) as well (Additional file 3: Figure S10B). We also confirmed these results in RKO cells, where N-BLR was transiently knocked down using the N-BLR siRNA1 + 3 pool (Additional file 3: Figure S10C). On the contrary, in the transiently overexpressing N-BLR RKO cells that were used for the migration/invasion assays shown in Additional file 3: Figure S8, the levels of miR-141-3p and miR-200c-3p were significantly reduced compared with cells transfected with empty vector control (Additional file 3: Figure S10D). Similarly, when we transfected RKO cells with either miR-141-3p or miR-200c-3p mimics, the levels of N-BLR were decreased by ~30% (Additional file 3: Figure S11). We confirmed a direct molecular coupling between both miR-141-3p and miR-200c-3p and N-BLR using luciferase assays and constructs carrying either the wild-type (WT) or the mutant miRNA response element sites within N-BLR (Fig. 4b). Given the above-mentioned involvement of N-BLR in the EMT, and of the miR-200 family in the EMT, we conclude that N-BLR and the two miRNAs are linked into a feedback loop that regulates the events occurring during EMT.

We also wanted to assess whether this interaction also occurs in tumor tissue. To this end, we measured the levels of miR-141-3p and miR-200c-3p and compared them with those of N-BLR by pair-matching individual tissue cores. We found an inverse correlation between the levels of miR-141-3p and miR-200c-3p on one hand and those of N-BLR on the other, as measured by ISH in the same tissue cores from the TMA (Fig. 4c and d). Particularly in adenocarcinoma, high N-BLR levels were associated with low levels of miR-141-3p and miR-200c-3p. Furthermore, the levels of miR-141-3p and miR-200c-3p, as measured by qRT-PCR, were significantly lower in CRC tumors than in normal colon samples (Additional file 3: Figure S12A and B left). We also evaluated if this inversed correlation between the levels of miR-141-3p and miR-200c-3p and those of N-BLR was associated with the clinical outcome of CRC patients. We found that low levels of both miR-141-3p and miR-200c-3p were associated with a poor OS of CRC patients (Additional file 3: Figure S12A and B right), and high levels of N-BLR associated with poor OS (Fig. 1c and d). This further confirmed indirectly the inverted correlation between the two short ncRNAs (miR-141-3p and miR-200c-3p) and the lncRNA N-BLR.

Having established that the levels of N-BLR are inversely correlated to those of miR-141-3p and miR-200c-3p, we sought to determine whether this finding persists in clinical samples as well. Indeed, we observed an inverse relationship between N-BLR and E-cadherin levels in our first cohort (Additional file 4: Table S3) of CRC patients (Fig. 4e). We observed the same pattern when we compared adenocarcinoma cases having tumor positive lymph-nodes (i.e. metastases to the lymph nodes) with tumor negative (Fig. 4f). These results showed that the N-BLR expression levels can affect the epithelial phenotype of tumor cells (E-cadherin levels) and accordingly regulate their ability to migrate.

### N-BLR modulates resistance to 5-fluorouracil (5-FU) through miR-200c-3p and XIAP

Because it has been reported that miR-200c-3p can target XIAP in pancreatic beta cells [46], we sought to determine whether the finding extends to the CRC context. Indeed, increased levels of miR-200c-3p were associated with significant decreased levels of the mRNA of its target gene XIAP (Additional file 3: Figure S6E). Interestingly, increased levels of XIAP are known to reduce the 5-FU-induced apoptosis and increase 5-FU resistance in CRC [47]. Having established above that N-BLR can regulate miR-200c-3p levels, we assessed whether N-BLR and miR-200c-3p play a role in regulating the 5-FU-induced apoptosis. To this end, we transiently transfected Colo320 with miR-200c-3p mimic. After 72 h, we treated the cells with different concentrations of 5-FU. The ectopic expression of miR-200c-3p led to the downregulation of XIAP at both mRNA and protein level (Additional file 3: Figure S13A left) and rendered Colo320 cells more susceptible to 5-FU-induced apoptosis (Additional file 3: Figure S13A right). To corroborate these results, we tested the HCT116 and RKO clones that stably overexpressed WT N-BLR. Both cell clones exhibited a small but statistically significant increase in their ability to resist to 5-FU-induced apoptosis compared with clones that stably expressed the empty vector (Additional file 3: Figure S13B). When RKO cells were transiently transfected to overexpress WT N-BLR, we measured a decrease in the levels of miR-200c-3p, as expected, and, again, a concomitant small but statistically significant increase in the levels of XIAP and in the ability to resist 5-FU-induced apoptosis (Additional file 3: Figure S13C).

### The 20-nt pyknon motif in N-BLR influences its interaction with miRNAs

**Fig. 5:**
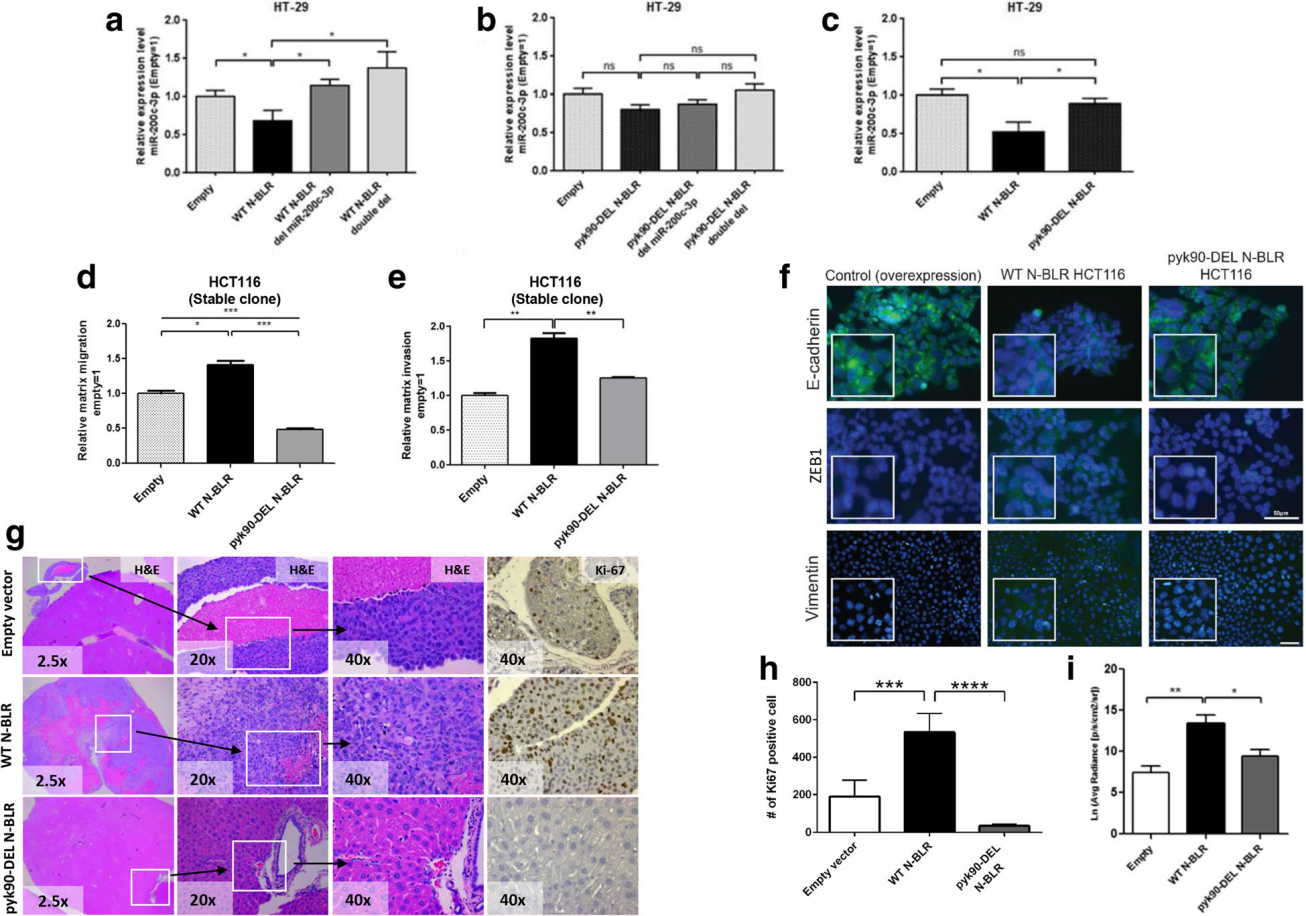
The 20-nt pyknon motif influences the functional role of N-BLR. **a**. miR-200c-3p levels following 48 h of co-transfection with empty pcDNA 3.1 vector, WT N-BLR vector, WT N-BLR del miR-200c-3p, WT N-BLR double del for both miR-200c-3p and miR-141-3p binding sites in HT-29 cell lines. The levels of miR-200c-3p were significantly reduced by the overexpression of the WT N-BLR compared to the empty vector, whereas they were restored by the overexpression of the mutant vector. **b** miR-200c-3p expression levels following 48 h of co-transfection with empty pcDNA 3.1 vector, pyk90-DEL N-BLR vector, pyk90-DEL N-BLR del miR-200c-3p, pyk90-DEL N-BLR double del for both miR-200c-3p and miR-141-3p binding sites. The lack of the pyk90 motif within N-BLR is likely to critically impair the binding between N-BLR and miR-200c-3p, thus the levels of the miRNA do not decrease significantly compared to the empty vector. **c** Comparison of miR-200c-3p expression levels between WT N-BLR and pyk90-DEL N-BLR cells: the binding of miR-200c-3p to N-BLR is partly dependent on the presence of the pyk90 motif. *Y-axis* values represent the ratio of miR-200c-3p and miR-141-3p to U6. Ratios were calculated with the 2^–ΔCt^ method using U6 levels for normalization. For each set of co-transfection experiments, the expression levels of miR-200c-3p were corrected by subtracting the values derived from the corresponding miRNA mimic negative control. Data are shown as mean ± SEM: n = 4. **d** Migration assays at 24 h show a significant increase of migrating cells with stable overexpression of WT N-BLR. Conversely, stable overexpression of pyk90-DEL N-BLR leads to a dramatic decrease of migratory capabilities even compared to the empty vector stable clone. **e** Similarly to migration, invasion assays at 36 h identified a significant increase of the invading population among the stable WT BLR overexpressing cells compared with the empty vector stable clone. While overexpression of pyk90-DEL N-BLR did not produce such gain of function, although not significant, it still conferred an edge of invasion over the empty clones. Data are shown as mean ± SEM: n = 3. **f** E-cadherin, ZEB1, and vimentin detection by immunofluorescence in HCT116 N-BLR overexpressing clones. The signal of E-cadherin (*green color*) was markedly decreased in WT N-BLR clone. The ZEB1 signal was absent in cells with empty vector (*green color*) but visible in WT N-BLR overexpressing clone. *Blue color* indicates nuclei. Single *green*, *blue*, and merged channel images of ZEB1 are reported in Additional file 3: Figure S9C. **g** Representative H&E images and immunohistochemical staining of Ki67 in liver metastases from nude mice after approximately four to six weeks of intrasplenic injection with empty vector, WT N-BLR, and pyk90-DEL N-BLR overexpressing HCT116 clones are shown. **h** Quantification of Ki-67 staining is reported. **i** WT N-BLR enhances liver metastases in the injected mice. Weekly imaging was performed using the Xenogen IVIS spectrum system within 12 min following injection of D-Luciferin (150 mg/mL). Living image 4.1 software was used to determine the regions of interest (ROI), and average photon radiance (p/s/cm2/sr) was measured for each mouse. Data were log-transformed before analysis. Data are shown as mean ± SEM: EMPTY n = 4, WT N-BLR n = 5, pyk90-DEL N-BLR n = 7. (Student’s t-test; *p < 0.05; **p < 0.01; ***p < 0.001; ****p < 0.0001)

Next, we examined whether the 20-nt pyknon motif from the 844-nt long N-BLR transcript could affect the direct coupling of miR-141-3p and miR-200c-3p to N-BLR. According to our in silico miRNA target predictions, a segment of the miR-200c-3p binding site is shared with the 5′ region of the pyk90 motif (Additional file 3: Figure S14A). We constructed pcDNA3.1 plasmids containing either WT N-BLR or pyk90-deleted N-BLR (pyk90-DEL construct from position 779 to 798 of N-BLR); then, for each of the two N-BLR variants we constructed a set of mutant vectors carrying the deletion either for miR-141-3p or miR-200c-3p binding sites or both (Additional file 3: Figure S14B). N-BLR overexpressing vectors were transiently co-transfected with either miR-141-3p or miR-200c-3p into HT-29 cells. As expected, ectopic expression of WT N-BLR significantly reduced the levels of miR-200c-3p and miR-141-3p compared with the corresponding variants containing the deleted binding sites for each miRNA (Fig. 5a and Additional file 3: Figure S14C-E). In both cases, a non-significant effect of the double deletion was observed compared with the single deletion, supporting the specificity of each miRNA for the correspondent N-BLR interaction region and the reliability of our predictions. More interestingly, the ectopic expression of the pyk90-DEL N-BLR transcript, which lacks part of the miR-200c-3p binding site, could not induce the reduction of miR-200c-3p levels (Fig. 5b and c), whereas it was still able to significantly affect miR-141-3p levels (Additional file 3: Figure S14E and F). These results suggest the importance of this primate-specific pyknon motif (pyk90). They also suggest that other valuable pyknon-containing transcripts await discovery.

### miR-141-3p and miR-200c-3p interaction with N-BLR influence ZEB1 expression

Having shown the inverse correlation between N-BLR and N-BLR and the ZEB1-targeting miR-141-3p and miR-200c-3p, we sought to determine whether the modulation of N-BLR could influence the expression levels of ZEB1 and, by extension, the levels of E-cadherin. To this end, we ectopically induced the expression of N-BLR in HT-29 cells, which have low endogenous levels of N-BLR (Additional file 3: Figure S7A). We used individual vectors containing the following sequences: (1) WT N-BLR; (2) N-BLR with the miR-141-3p binding site deleted (WT N-BLR del miR-141-3p); (3) N-BLR with the miR-200c-3p biding site deleted (WT N-BLR del miR-200c-3p); and (4) N-BLR with both the miR-200c-3p and miR-141-3p binding sites deleted (WT N-BLR double del). We found that upon overexpression of WT N-BLR, the levels of ZEB1 were increased compared to the empty vector control, in concordance with the rest of our findings. On the other hand, we did not measure any change in ZEB1 levels compared with the empty vector control when we overexpressed the three N-BLR constructs carrying the deletions for miR-141-3p and miR-200c-3p binding sites (Additional file 3: Figure S15A). We also confirmed in RKO cells that transient transfection with the WT N-BLR vector could lower the levels of miR-141-3p and miR-200c-3p (Additional file 3: Figure S10D) and could increase the levels of ZEB1 (Additional file 3: Figure S15B). In an analogous experiment, when we transfected RKO cells with miR-141-3p and miR-200c-3p mimics, we were able to lower the levels of ZEB1 measured at 48 h following the transfection (Additional file 3: Figure S15C). These results suggest that the upregulation of N-BLR expression in colon cancer cells could regulate the acquisition of EMT phenotype by buffering the levels of both miR-141-3p and miR-200c-3p resulting in the upregulation of their target gene ZEB1.

### Deletion of the pyknon motif from the N-BLR transcript has functional consequences

In light of the above data, we further investigated the impact of deleting the 20-nt pyknon motif on N-BLR’s ability to regulate migration, invasion, and colony formation. As expected, stably overexpressing WT N-BLR in HCT116 cells significantly increased their ability to migrate and invade compared with cells stably expressing empty vector control. On the other hand, when we overexpressed the pyk90-DEL N-BLR vector we did not observe any notable increase in migration and invasion (Fig. 5d and e). We independently confirmed these results by transiently overexpressing N-BLR vectors in HCT116 cells (Rigoutsos laboratory) (Additional file 3: Figure S16A). We also found that overexpression of the WT N-BLR increased the cells’ ability to form colonies compared to the empty vector, whereas the overexpression of the pyk90-DEL N-BLR vector did not have any significant effect (Additional file 3: Figure S16B). Furthermore, by immunofluorescence analysis, we observed a reduction of the expression of E-cadherin and an increased expression of both ZEB1 and vimentin in WT N-BLR HCT116 cells compared with the empty vector clone (Fig. 5f and Additional file 3: Figure S9C). We also evaluated the effect of deleting only the portion of the pyk90 motif that is in between the miR-141-3p and miR-200c-3p binding sites (pyk90-DEL2 N-BLR, from position 784 to 798 of N-BLR). This deletion did not affect the migration and invasion ability of RKO and HCT116 cells, which continued to behave similarly to cells transfected with the WT N-BLR (Additional file 3: Figure S17A and B). These results further supported the critical role of the WT pyk90 motif in affecting N-BLR’s functions, which regulate key molecular factors involved in the development of the aggressive cancer cell phenotype (EMT phenotype, increased migration and invasion, and increased colony formation ability).

Finally, to corroborate the relevance of these findings, we evaluated the ability of N-BLR to regulate the malignant phenotype of tumor cells using an *in vivo* model of metastasis. Nude mice underwent intra-splenic injection of stably overexpressing either WT N-BLR or pyk90-DEL N-BLR or expressing the empty vector (control). The metastatic spread to the liver of HCT116 was assessed by histological examination and bio-luminescence assay. HCT116 cells that overexpressed WT N-BLR showed an increased ability to colonize and invade the liver, as demonstrated by the massive infiltration of liver tissue by tumor cells and higher proliferative index (Ki-67 levels) resulting in increased metastatic burden. On the other hand, the HCT116 cells that overexpressed the control or the pyk90-DEL N-BLR vectors showed reduced metastatic potential (Fig. 5 g and h). These findings were confirmed independently by bioluminescence assay (Fig. 5i). These *in vivo* data further support the biological importance of the pyknon motif within the span of the N-BLR transcript.

### Genome-wide profiling of pyknon transcripts

In light of the many and diverse observations we reported above, we conjectured that the genomic instances of the pyknon DNA motifs could serve as “homing beacons” that might allow us to locate lncRNAs with potential functional relevance. To investigate this possibility, we built a custom microarray. We prioritized among the more than 209,000 human pyknons [30, 32] by focusing on a subset of pyknon instances that occur in the previously reported “cancer associated genomic regions” or “CAGRs” [37] and are either intergenic or intronic. We identified 1292 such locations that are distributed across all chromosomes (Additional file 3: Figure S18A) and correspond to 300 unique pyknon motifs. The probes of the array were designed to investigate transcription from the forward and the reverse strands of the genome at these 1292 locations. Specifically, we centered a 100 nt window at each pyknon instance and designed a 40 nt probe within the window that overlapped with the corresponding pyknon (Additional file 3: Figure S18B). At each location, probes were designed separately for each strand. In 230 instances, the candidate probe sequences did not pass quality control leaving us with a grand total of 2354 array probes. For comparison purposes, probes for human miRNAs were added to the microarray. A standardized list of all known human pyknons together with a complete list of their coordinates across the span of the human genome can be found at http://cm.jefferson.edu/pyknons.html.

### Unique and non-unique probes reveal tissue-specific expression profiles and disease-specific profiles that correlate with patients’ OS

**Fig. 6:**
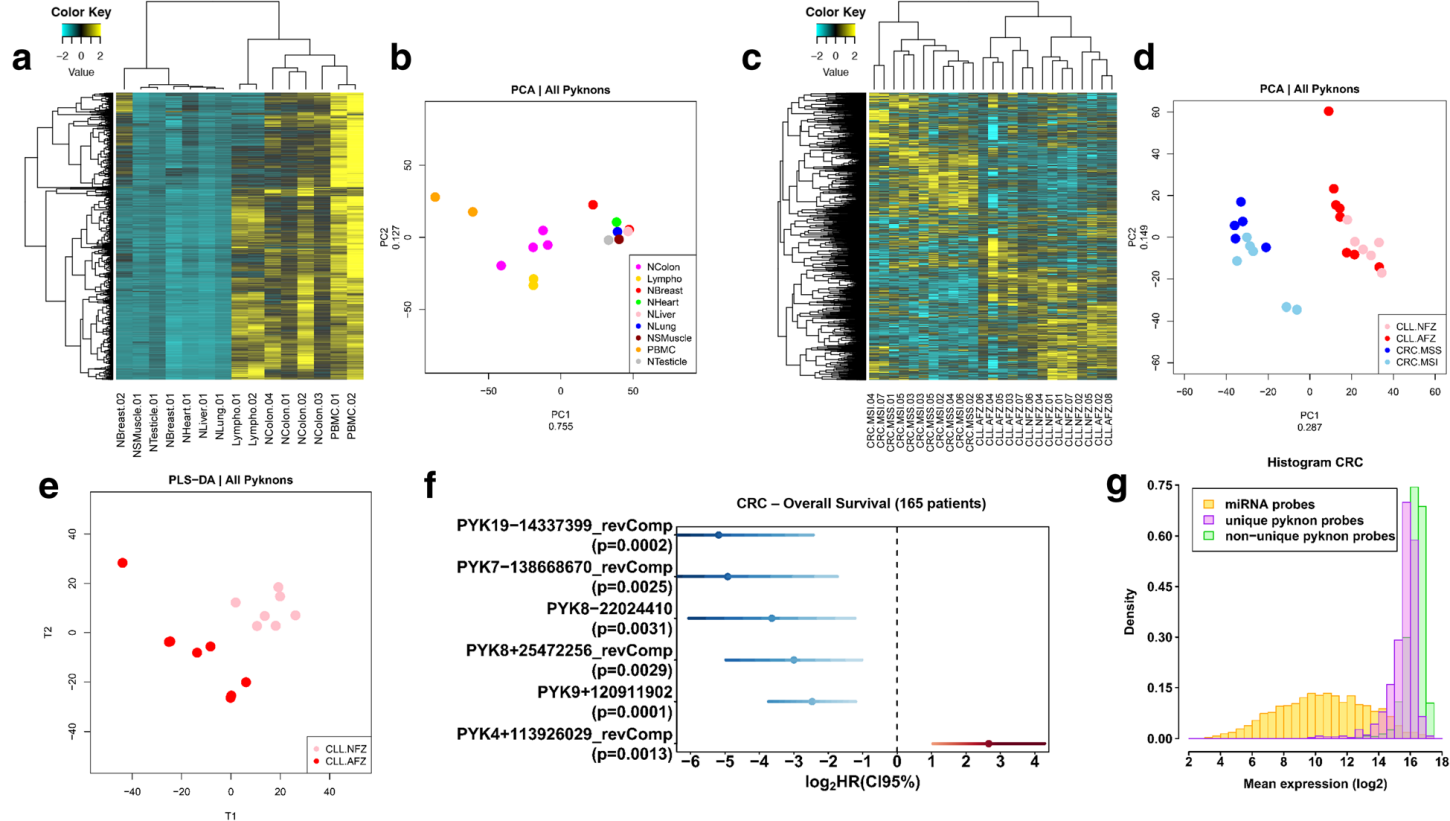
Pyknon expression across tissues and tissue states. **a**–**e** Pyknon clusters showing tissue and disease specificity among normal (**a**, **b**) or diseased (**c**–**e**) tissue samples. **a**, **c** *Heatmaps* of standardized pyknon expression profiles. *Dendrograms* were constructed with Hierarchical Clustering using Pearson correlation as a metric. **b**, **d** Principal component analysis (PCA) of the normal (**b**) or the diseased (**d**) samples. The *X-axis* corresponds to the first principal component (PC1) and the *Y-axis* to the second principal component (PC2). The numbers next to the PC labels represent the amount of information from the original dataset that is projected on each one. **e** Partial Least Squares-Discriminant Analysis showing the perfect separation of the samples with normal (CLL-NFZ) or aberrant (CLL-AFZ) FISH profile of chromosome arm 17p and ZAP-70 levels. *CRC-MSS* colorectal cancer sample without microsatellite instability, *CRC-MSI* colorectal cancer sample with microsatellite instability, *Lympho* B-lymphocytes, *NBreast* normal breast tissue, *NColon* normal colon tissue, *NHeart* normal heart tissue, *NLiver* normal liver tissue, *NLung* normal lung tissue, *NSMuscle* normal skeletal muscle tissue, *NTesticle* normal testicle, *PBMC* mononuclear cells. **f** The COX OS analyses of the pyknon expression using the genome-wide array identified a set of six transcribed pyknons that are associated at a *p* < 0.01 with OS in CRC. All these six probes were chosen for the analyses because they correspond to an unambiguous genomic location. The *blue bars* correspond to a negative HR, meaning an association with good prognosis, while the *red bar* correspond to a positive HR, meaning an association with poor prognosis. **g** Expression of probed pyknons in comparison with human miRNAs. Pyknon transcription levels are higher than those of miRNAs—probability density values of normalized intensities for the miRNA and pyknon probes across all 165 CRC arrays used for the data from Fig. 6f

We collected 15 normal samples from different individuals that spanned nine different tissues (four colon, two breast, one lung, one heart, one skeletal muscle, one testicle, one liver, two mononuclear cells, and two B-lymphocytes). We used our microarray to examine potential expression from the genomic regions interrogated by the probes. By analyzing normal samples, we found several pyknon profiles that clustered according to the tissue of origin, which in turn suggests the existence of tissue-specific pyknon signatures (Fig. 6a and b). In fact, the pyknon probes exhibited higher tissue specificity in normal tissues compared to miRNAs as gauged by the Spearman correlation (Additional file 3: Figure S19). Furthermore, pyknon transcript signatures distinguished healthy colon from CRC samples and CLL from healthy B-cell samples (Additional file 3: Figure S20). Using an independent approach (qRT-PCR), we confirmed the data obtained from the array for selected pyknon-regions comparing leukemia samples with normal B cell counterparts (pyk-reg-14 in Additional file 3: Figure S21A) and normal colon samples with colon cancer samples (pyk-reg-10 and pyk-reg-40 in Additional file 3: Figure S21B and C).

We also used the array to examine the expression of pyknon-containing transcripts in diseased samples. We identified that pyknon expression differentiates CRC tissues from the most frequent leukemia in the Western world, the chronic lymphocytic leukemia (CLL) [48] (Fig. 6c and d). We also showed that pyknon signatures could distinguish CLL samples with good versus poor prognosis as characterized by the levels of the tyrosine kinase ZAP-70, one of the most widely used prognostic marker in CLL and also by 17p deletion (Fig. 6e).

Since we determined that N-BLR expression was significantly associated with the OS in CRC patients, we further explored if the full set of pyknon transcripts we identified using this custom array was also associated with OS. To this end, we collected a fourth set of 165 CRC patients (Additional file 11: Table S8) for which clinical data were available as well as enough RNA material for the array hybridization. By performing COX analysis, we identified a set of six pyknon-transcript probes of unambiguous genomic origin (unique probes), associated at a *p* < 0.01 with patients’ survival (Fig. 6f). We further identified that pyknon-containing transcripts probed by unique probes exhibit higher expression levels than the miRNAs in these samples (Fig. 6 g). Furthermore, the expression of a set of 122 pyknon transcript probes was an independent prognostic factor for OS when analyzed by the COX model (Additional file 12: Table S9). These data demonstrate that the expression of transcripts containing the organism-specific pyknon motifs are not only tissue-specific but also disease-specific and support their potential use as novel biomarkers for the identification of tissue-specific and cancer-specific pathogenic mechanisms.

## Discussion

In this work, we presented our findings on N-BLR, a pyknon-containing primate-specific lncRNA and a novel modulator of the EMT process and apoptotic pathway in CRC. N-BLR localizes to the cytoplasm where it directly interacts with miR-141-3p and miR-200c-3p, two members of the highly conserved miR-200 family known to inhibit EMT [44]. We particularly observed that an increase in the levels of N-BLR was associated with decreased levels of miR-141-3p and miR-200c-3p and accordingly increased levels of ZEB1, whereas a decrease of N-BLR levels was associated with opposite effects on miR-141-3p, miR-200c-3p, and ZEB1 expression. In addition, the increase in the levels of ZEB1 induced by N-BLR upregulation was associated with acquisition of EMT phenotype (downregulation of E-cadherin and upregulation of vimentin), whereas the decrease of ZEB1 levels induced by N-BLR knockdown had opposite effects. These results made us conclude that these three non-coding transcripts (N-BLR, miR-141-3p, and miR-200c-3p) and three coding genes (E-cadherin, vimentin, and ZEB1) comprise a new component of signaling interactions in the EMT pathway. N-BLR also plays an important role *in vivo*: indeed, we found that the overexpression of WT N-BLR endowed colon cancer cells with increased ability to metastasize and invade liver compared with the overexpression of N-BLR harboring a pyk90 deletion (pyk90-DEL N-BLR). Our results are in concordance with the recent finding that miR-200c-3p plays an important role in controlling EMT and the metastatic process of colon cancer cells to the liver [49].

A key element to the discovered interactions is the 20 nt pyknon motif that is contained near the 3′ end of N-BLR. This human-specific motif partially overlaps with the binding of the EMT-regulating miR-200c-3p and our deletion studies proved that these interactions are functionally important. Indeed, we showed that targeted deletion of the motif affected colony formation, invasion, and migration, whereas the minimal deletion of the part of pyknon region that is not included in the miRNA binding site (the direct interaction is not abolished in this case) had no functional effects. It is important to stress that many more interactions are likely to occur between N-BLR and miRNAs and influence the malignant phenotype and these have to be further explored in a systematic way.

We also found that N-BLR acts as an inhibitor of apoptosis. We particularly showed that increased levels of N-BLR were associated with a decrease in miR-200c-3p and increase in XIAP expression levels. It was reported that miR-200c-3p could target XIAP, thereby leading to decreased levels of XIAP and cell viability [46]; tumor cells were more resistant to the apoptosis induced by 5-FU, when they express higher levels of XIAP [47]. Unsurprisingly, we found that ectopic expression of miR-200c-3p induced increased susceptibility to 5-FU-induced apoptosis. Conversely, N-BLR-mediated decrease of levels of miR-200c-3p was associated with increased levels of XIAP and resistance to 5-FU-induced apoptosis. On the other hand, the decreased levels of N-BLR were associated with a concomitant increase in miR-200c-3p levels, downregulation of the inhibitor of apoptosis XIAP and a subsequent upregulation of caspase activity (Caspases 3/7, 8, and 9) and levels of cleaved PARP-1, resulting in increased levels of apoptosis. Based on all these findings, we would expect an associated increased resistance to apoptosis in those CRC settings where N-BLR is upregulated. This might explain, at least in part, the association between increased N-BLR levels and poor prognosis that we observed in two independent cohorts from two different patient populations (Ferrara, Italy and Dallas, Texas).

In summary, our findings suggest a model whereby N-BLR may mediate the switch from an epithelial to a mesenchymal cell phenotype by sequestering miR-141-3p and miR-200c-3p. This would result in the upregulation of ZEB1, which in turn directly suppresses E-cadherin. Thus, in this context, an increase in the expression levels of N-BLR, such as we observed in the cell lines and the CRC samples, can induce a concomitant interaction between N-BLR and available copies of the endogenous miR-141-3p/miR-200c-3p pool resulting in a reduced targeting of ZEB1. In turn, the increase of ZEB1 expression levels can induce a consequent decrease of E-cadherin levels and the transition toward a mesenchymal phenotype resulting in an increase in migratory and invasive potential. Moreover, the reduction of free miR-200c-3p can increase the levels of its target XIAP, resulting in an increased ability to resist apoptotic stimuli, including those related to the current chemotherapy drugs for CRC patients (such as 5-FU).

Analogous interactions for a different lncRNA (lncRNA-ATB) were recently reported in a different disease context [50]. LncRNA-ATB was shown to promote invasion and metastasis in hepatocellular carcinoma through interactions with members of the miR-200 family and with ZEB1/ZEB2. Inspection of the genomic sequence of lncRNA-ATB reveals that it is a composite of three LINE-1 retrotransposon fragments and one full-length SINE retrotransposon. The latter has numerous other instances in the human genome. This raises the possibility that N-BLR may be one of several lncRNAs that could be involved in very complex interactions such as those that we described in [29, 30, 32, 51] and more recently in [31].

Furthermore, our work expands the potential number of primate-specific transcripts from the few already identified to date (for an interesting example, see ref [52]) to potentially tens of thousands, as most of the pyknon DNA regions that we examined show evidence of transcription. We already generated several lines of evidence that additional genomic instances for pyk90 outside the chromosome 3 location of N-BLR are actively transcribed (Ling H and Calin GA, data not shown). Therefore, the pyknon-containing-transcripts, even if each is expressed at lower levels than coding genes, due to their much larger number could represent an efficient system that uses sequence-complementarity to buffer highly expressed miRNAs and potentially exogenous sequences such as viral transcripts or to achieve regulatory control as part of normal post-transcriptional regulation [51]. It is also worth mentioning that the N-BLR transcript is primate-specific and thus not conserved in rodents. As such, N-BLR’s activity cannot be captured by mouse models of colon cancer. This represents another intriguing dimension of the intricacies of human disease and highlights the importance of discovering N-BLR’s regulatory control of the EMT and apoptosis. In this regard, N-BLR and other similar molecules would be different from miRNAs [10], transcribed UCRs [53], or lincRNAs [54]. In fact, organism-specific transcripts can be thought of as representing a paradigm shift supported by the increasing realization that human cancers differ from animal models involving the same gene and the specific human mutation [55]. These properties of primate-specific transcripts make N-BLR and similar molecules promising as novel prognostic indicators. Our data also have potential implications for the cell-to-cell communication and the development of new lncRNA-based therapeutics [56].

## Conclusions

Our work discussed the discovery and study of N-BLR, a primate-specific lncRNA. Our analyses indicate that N-BLR is a novel molecular player in the mechanisms underlying the metastatic potential in CRC. This, together with our pyknon microarray findings, suggests that N-BLR and likely other transcripts among those that were profiled by the microarray could prove important to our understanding of key molecular processes and might potentially find uses as novel biomarkers or novel therapeutics in human cancers and other diseases.

## Methods

### Patient samples

This study made use of four cohorts of patients. The first cohort, including 127 colon samples and 28 adjacent normal mucosa collected between 2003 and 2008, was obtained from the Department of Experimental and Diagnostic Medicine, University of Ferrara, Ferrara, Italy (Dr. Giovanni Lanza and Dr. Roberta Gafà) (Additional file 4: Table S3). For 114 samples, complete follow-up information was available and was used for the survival analyses. The second cohort of 170 colorectal cancer samples was obtained from the Center for Gastrointestinal Research and Center for Epigenetics, Baylor Research Institute and Charles A. Sammons Cancer Center, Dallas, Texas, USA (Additional file 7: Table S5). The third cohort of 21 metastatic colon cancer samples was obtained from an independent source (Dr. Jen-Jen Yeh, University of North Carolina, USA) (Additional file 10: Table S7). The fourth cohort of patients included 165 patients with primary CRC adenocarcinoma that underwent surgical resection of primary tumor at the University of Texas MD Anderson Cancer Center (UTMDACC) during July 2001 to July 2009 (Additional file 11: Table S8). There were 85 male and 80 female patients with a median age of 53 years (range = 29–94 years). Most of them were stage II–III (153 patients) and 12 were stage IV CRC; none of them had received neoadjuvant treatment. Among the stage II-III patients, 95 received adjuvant chemotherapy of 5-FU based regimen plus oxaliplatin or irinotecan with a median of eight cycles (range = 1–12 cycles). Median follow-up time was 8.6 years. All these tissue samples were obtained from fresh surgical specimens, snap-frozen in liquid nitrogen, and stored at –80 °C. All samples were obtained after histology confirmation. Nineteen peripheral blood samples (15 CLL and four normal) were also used in this study.

### RNA extraction and qRT-PCR

Total RNA from both tissues and cell lines was isolated by using TRIzol reagent (Invitrogen, Carlsbad, CA, USA) and DNase-digested (Ambion), according to manufacturers’ instructions. RNA from nuclear and cytoplasmic compartment was isolated using Ambion’s Protein and RNA Isolation System, PARIS™ Kit (ThermoFisher Scientific). Total complementary DNA (cDNAs) was reverse transcribed using SuperScript III cDNA kit (Invitrogen) with random hexamers, according to the manufacturer’s protocol. qRT-PCR analysis was carried out with iQ SYBR Green Supermix (Bio-Rad) and gene-specific primers (Additional file 2: Table S2). For the quantification of XIAP mRNA, TaqMan Gene Expression Assay probe (ThermoFisher Scientific) was used. For the quantification of N-BLR, either specific primers from Additional file 2: Table S2 or customized TaqMan Gene Expression Assay probe were used. For the quantification of ZEB1 and GAPDH mRNA, either specific primers from Additional file 2: Table S2 or TaqMan Gene Expression Assay probes were used. For pyknon-containing regions, we centered a 100-nt region at each pyknon and used the Primer3 program to design 20-nt primers for each window manually. We carried out qRT-PCR and then products were loaded on 3% agarose gels. Only primers that showed a single clear band and good melting curve were selected and products were confirmed by sequencing. U6 snRNA was employed as endogenous control. For miRNA analysis, 10 ng of RNA were used for cDNA synthesis with specific stem-loop RT primers for miR-200c-3p, miR-141-3p, and U6 snRNA by TaqMan MicroRNA Reverse Transcription Kit (ThermoFisher Scientific) according to the manufacturer’s protocol. Real-time PCR was performed as above, using TaqMan microRNA assays (#002300, #000463, and #001973, ThermoFisher Scientific). The 2^–ΔCt^ method was used to calculate the relative amount of each transcript compared with expression of endogenous control (U6 and GAPDH). If expression values for the RNA of interest were not obtained after 35 cycles of amplification in two successive experiments in duplicate wells, then the specific values were considered not available.

### Cloning pyknon-containing regions

We used the GeneRacer kit (Invitrogen) to carry out the rapid amplification of cDNA ends (RACE) method for N-BLR. The kit was used in accordance with the manufacturers’ protocols. We obtained cDNA from DNase-treated total RNA from HCT116 cell (2 μg). The 5′- and 3′-RACE products were cloned into pCR4-TOPO (Invitrogen) and transformed into *E. coli* TOP10 cells. Cloned RACE products were fully sequenced in both directions.

### *In vitro* translation assay

To test the translation potential of investigated lncRNAs, we performed *in vitro* translation assay using TnT® T7 Quick Starter Bundle Chemiluminescent (Cat No. L1210, from Promega, Madison, WI, USA) according to the manufacturer’s instructions. Briefly, reaction components including TNT® T7 Quick Master Mix, Methionine, plasmid DNA template (pcDNA3.1 empty vector or luciferase T7 positive control vector or pcDNA-pyk90 vector) and Transcend™ Biotin-Lysyl-tRNA were incubated at 30 °C for 90 min. Once the 50 μL translation reaction is complete, 1 μL aliquot was added into 15 μL of SDS sample buffer, heated at 90–100 °C for 2 min, loaded on an SDS-polyacrylamide gel, and transferred to a nitrocellulose membrane using a semi-dry system. The Transcend™ Non-Radioactive Translation Detection Systems (Cat No. L50811, from Promega, Madison, WI, USA) was used for the detection of proteins synthesized *in vitro* according to the manufacturer’s instructions. Additionally, the luciferase activity in the positive control was verified with a luciferase assay measured with a microplate luminometer.

### SiRNA studies

We designed siRNAs against N-BLR using the Dharmacon algorithm (Dharmacon siDESIGN http://www.dharmacon.com/sidesign/). Each of four highest-ranking siRNA sequences for N-BLR was tested in our experiments. These siRNAs were re-suspended in 1X siRNA buffer (Dharmacon, LaFayette CO, USA) to a stock concentration of 50 μM. The performance was assessed at 24 h intervals post-transfection by qRT-PCR. The cells were transfected with the corresponding siRNA pool at the final concentrations indicated in the main text by using Lipofectamine 2000 (Invitrogen) according to the manufacturer’s protocol for further analysis. As control, we used a pool of non-targeting siRNAs (Dharmacon).

### Cell count and viability

Colo320, SW620, and SW480 cells were cultured in RPMI 1640 1X with L-Glutamine medium (#10-040-CV, Corning Cellgro), whereas HCT116, RKO, and HT-29 cells were cultured in McCoy’s 5A 1X with L-Glutamine medium (#10-050-CV, Corning Cellgro), supplemented with 10% fetal bovine serum and 1% penicillin and streptomycin. CRC cell lines were plated in 24-well plates at a concentration of 1 × 10^5^ cells/well in an antibiotic-free medium one day before transfection. After transfection, cells were collected using trypsin-ethylenediaminetetraacetic acid (EDTA) (Mediatech) and the cell count and viability were determined by using the Vi-cell Viability Analyzer 1.01 (Beckman Coulter, Fullerton, CA, USA) at 0, 24, 48, 96, and 120 h following siRNA transfection. The cell viability for the apoptosis induced by 5-FU was measured with CellTiter 96® AQueous One Solution Cell Proliferation Assay (MTS) (Promega).

### Apoptotic assays

Cells were plated in six-well plates at a concentration of 5 × 10^5^ cells/well in an antibiotic-free medium one day before transfection. We harvested cells at 48, 96, and 120 h following transfection, using trypsin-EDTA (Mediatech) and dissolved in NP40 lysis buffer (0.5% NP40, 250 mM NaCl, 50 mM Hepes, 5 mM EDTA, 0.5 mM egtazic acid) freshly supplemented with a complete protease inhibitor and phosphatase inhibitor cocktails 1 and 2 (Roche). Proteins were purified and the levels of PARP protein quantified with the rabbit polyclonal anti-PARP1 antibody (Cell Signaling Technology) using standard procedures for Western blotting. Normalization was performed with mouse monoclonal anti-ACTB antibody (Cell Signaling Technology). For further confirmation of apoptosis, Colo320, SW620, SW480, RKO, and HCT116 cell lines were analyzed using the Caspase-3/7, 8, and 9 assays according to the manufacturer’s protocol (Promega, Madison, WI, USA). To dissect the detailed pathway of apoptosis, we used antibodies specific to XIAP (Cell Signaling Technology) and c-IAP1 (Cell Signaling Technology).

### N-BLR shRNA and overexpressing stable clone establishment

We transfected vectors containing pSuper.retro.puro shRNA (OligoEngine, Seattle, WA, USA) specifically designed against the gene in HCT116 cells by Lipofectamine 2000 (Invitrogen), according to manufacturer’s guidelines. Clone selection was performed with G418 (2 mg/mL), and the expression level of N-BLR was tested by qRT-PCR. For construction of lentiviral vector expressing N-BLR gene, human N-BLR was PCR-amplified by Pfu Ultra II Fusion HS DNA Polymerase (Stratagene, Agilent Technologies) from commercial Human Genomic DNA and subcloned into the XbaI and NotI sites of pCDH-CMV-MCS-EF1-puro lentiviral vector. The pyk90-DEL N-BLR variant was produced by using Quick Change II XL Site-Directed Mutagenesis kit (Stratagene, Agilent Technologies). Following infection, the cells were selected with puromycin (2 μg/mL).

### Vector construction and transient N-BLR/miRNA co-transfection

The WT and pyk90-DEL N-BLR sequences were PCR-amplified by Platinum® Taq DNA Polymerase High Fidelity (Invitrogen, Life Technologies) from the pCDH-CMV-MCS-EF1-Puro vectors used for the establishment of stable N-BLR overexpressing HCT116 clones and subcloned into the HindIII and XhoI sites of the pcDNA3.1 vector (Invitrogen). The pcDNA3.1-WT N-BLR and pcDNA3.1-pyk90-DEL N-BLR constructs carrying the single deletion for either miR-200c-3p or miR-141-3p binding sites, the double deletion for both miRs’ binding sites and the deletion between the miR-200c-3p and miR-141-3p binding sites synthesized by using Quik-Change II XL Site-Directed Mutagenesis kit (Stratagene, Agilent Technologies) and named WT N-BLR-del-miR200c, WT N-BLR-del-miR141, WT N-BLR double del, pyk90-DEL N-BLR-del-miR200c, pyk90-DEL N-BLR-del-miR141, pyk90-DEL N-BLR double del, and pyk90-DEL2 N-BLR. Transfections were performed using the Lipofectamine 2000 kit (Invitrogen) according to the manufacturer’s instructions. The mirVana miRNA Mimics hsa-miR-200c-3p, hsa-miR-141-3p (MC11714, MC10860, Life Technologies), and mirVana miRNA Mimic Negative Control #1 were used for transfection at a final concentration of 50 nM. The cells were harvested and RNA was extracted for qRT-PCR analysis after 48 h following transfection.

### Luciferase reporter assay

Luciferase reporter assay to confirm miRNA interactions were executed as we previously described [57].

### Cell cycle analyses

Cells were synchronized by serum starvation (0.1% FBS) for 48 h at 37 °C and then transfected with either siRNA scramble control or N-BLR siRNA1 + 3 at the concentration 100 nM. Cell cycle was analyzed 48 and 96 h after transfection by cytometry (BCI Gallios Analyzer, Beckman Coulter).

### Migration assays

Cell migration assays were performed according to modified protocol described previously [58]. Stable N-BLR shRNA expressing clones #3-1 and #4-7, stable N-BLR variants (WT and pyk90 DEL)-overexpressing clones, and the empty vector clone were re-suspended in serum free media (65,000 cells/insert) and seeded onto a 0.1% gelatin-coated inserts. After 24 h, cells that migrated to the bottom of the wells were fixed and stained with HEMA 3™ (Fisher Scientific, MA, USA) and counted by microscope. For each well, ten random fields were counted and the average number of cells was determined. The experiments were performed in triplicate. For transient transfection, RKO and HCT116 cells were harvested after 48 h following transfection with vectors containing either WT N-BLR, pyk90 DEL N-BLR, pyk90-DEL2 N-BLR, or empty control vector and seeded onto 0.1% gelatin-coated inserts for assessing migration as described above. Migration results were normalized by the total number of cells to minimize the effect of proliferation/viability.

### Invasion assays

Invasion assays were performed by using transwells with 8.0 μm porous membrane coated with an invasion matrix containing Type IV Collagen (#C6745-1ML, Sigma Aldrich), Human Laminin (# l6274), and Gelatin diluted in 1X PBS. HCT116 cells were transfected with siRNAs against N-BLR (N-BLR siRNAs1 + 3 pool) and control siRNAs at a final concentration of 100 nM for 48 h and then 300,000 cells were plated on the top of the transwell. The same number of cells was also plated in a separate culture well for normalization purposes (total cells). Each experiment was performed in triplicate. The same experiments were performed also with HCT116 stable shRNA N-BLR expressing clones #3-1 and #4-7, stable WT and pyk90 DEL N-BLR variants overexpressing clones, and the empty vector clone. The invasion assay was stopped after 36 h and cells were fixed and stained with HEMA 3. For each well, ten random fields were counted and the average number of cells was determined. For transient transfection of RKO and HCT116 cells, we followed the same protocol as for the migration assay. The invasion results were normalized by the total number of cells to minimize the effect of proliferation/viability.

### Colony formation assay

Colony formation assay was performed in HCT116 clones transiently overexpressing either WT N-BLR or pyk90-DEL N-BLR and compared to empty vector containing cells. Five hundred cells were seeded into a 60 mm dish and cultured for two weeks. Afterwards, cells were fixed by 100% methanol and stained with 0.2% crystal violet. Pictures were captured by GE imager (GE Healthcare Life Sciences) and colony number was counted.

### Colony formation in semi-solid agar

Six-well plates were pre-coated with 0.5% bottom agar layer with culture media. Then, cells were trypsinized, re-suspended in 0.4% upper agar layer, and seeded into the pre-coated six-well plate at the density of 500 cells per well, in triplicate. Each well was further overlaid with 0.3% agar on top. Colonies were checked after two weeks. Pictures were captured by GE imager (GE Healthcare Life Sciences) and colony number was counted.

### Immunofluorescence assays for E-cadherin and vimentin

About 0.8 × 10^5^ cells from clones #3-1 and #4-7 and empty vector clone were seeded on a 96-well plate. The experiments were done as previously described [59]. The cells were then incubated with anti-vimentin (V9, Novus) and anti-E-cadherin (BD Transduction) overnight, washed three times with PBST for 5 min, and finally incubated with secondary antibodies (Invitrogen) and DAPI. All matched samples were photographed (clones and empty cells) using a immunofluorescence microscope and identical exposure times. Each experiment was performed in triplicate.

### ISH for N-BLR

The frozen tissue sections were first digested with 5 μg/mL proteinase K for 5 min at room temperature and then loaded onto a Ventana Discovery Ultra system (Ventana Medical Systems, Inc, Tucson, AZ, USA) for ISH or immunohistochemistry analysis. The tissue slides were incubated with double-DIG labeled custom LNA probe for N-BLR (Exiqon) for 2 h at 55 °C. The miR-200c-3p and miR-141-3p LNA probes were purchased from Exiqon. The digoxigenins were detected with a polyclonal anti-DIG antibody and Alkaline Phosphatase conjugated second antibody (Ventana) using NBT-BCIP as the substrate. The double-DIG labeled control U6 snRNA probe is also from Exiqon. CK19 was detected using mouse anti-CK 19 antibody (1:200, Biogenex) and HRP conjugated anti-mouse antibody using DAB as the substrate (Ventana).

### Western blots

The western blot analysis was performed as previously described [60]. In brief, cells were lysed with Cell Lysis Buffer (Cell Signaling) containing Protease Inhibitor Cocktail (Sigma). Total proteins were separated by a 4–20% Criterion TGX Precast Gel (Bio-rad) and then transferred onto Trans-Blot® Turbo™ Midi Nitrocellulose Transfer Packs (Bio-rad). The membrane was incubated with primary followed by secondary antibodies after blocking with 5% non-fat milk (Bio-rad). Immunochemical detection was performed using either the Thermo Scientific™ SuperSignal™ West Femto Chemiluminescent Substrate (Thermo Scientific) or Amersham™ ECL™ Western Blotting Detection Reagent (GE Healthcare Life Science). The following antibodies were used at the dilution recommended by the manufacturer: β-actin (Sigma Aldrich, AC-15), GAPDH (Santa Cruz, sc-51905), XIAP (Cell Signaling, #2042), PARP (Cell Signaling, #9542), Survivin (Cell Signaling, 6E4), c-IAP (Cell Signaling, #4952), ZEB1 (Santa Cruz, sc-10572), and vinculin (Cell Signaling, E1E9V).

### Image analysis

To quantify the levels of N-BLR, miR-141-3p, and miR-200c-3p in the ISH of tissue microarray, images of each tissue core were automatically captured using a Perkin Elmer Caliper Vectra 2 microscope and then analyzed using inForm 2.0 image analysis software (Perkin Elmer, Inc., Waltham, MA, USA) [61, 62]. In particular, the quantification of N-BLR, miR-141-3p, and miR-200c-3p expression was automatically calculated as mean intensity measured within the tumor tissue (adenocarcinoma and metastatic), normal tissue, benign/polyp tissue, and colitis tissue. Non-epithelial tissue (e.g. stromal tissue) was excluded from the analysis. We excluded individual TMA cores, when they did not have enough tissue (epithelial versus non-epithelial tissue) for inForm 2.0 image analysis. Both image acquisition and analysis were performed at the North Campus Flow Cytometry and Cellular Imaging Core Facility at the UTMDACC (Co-director: Jared K. Burks, Department of Leukemia).

### Animal models and tissue processing experiments

Female athymic nude mice were purchased from the NCI, Frederick Cancer Research and Development Center (Frederick, MD, USA). These animals were cared for according to guidelines by the American Association for Accreditation of Laboratory Animal Care and the U.S. Laboratory Animals. All mouse studies were approved and supervised by the UTMDACC Institutional Animal Care and Use Committee. All animals used were aged six to eight weeks at the time of injection. For all the animal experiments, cells were trypsinized, washed, and re-suspended in Hanks’ balanced salt solution (HBSS; Gibco) before injection. For the intrasplenic cancer model, 1 × 10^6^ HTC116 cells per mouse in 50 uL HBSS were injected intrasplenic (experimental liver metastases model). The mice were anesthetized under isofluorane for splenic isolation and cell line injection (day 1), as well as the following day after injection (day 2) to perform splenectomy [50]. Liver metastases continued until mice in any group became moribund (approximately four to six weeks). Weekly imaging was performed using the Xenogen IVIS spectrum system within 12 min following injection of D-Luciferin (150 mg/mL). Living image 4.1 software was used to determine the regions of interest (ROI) and average photon radiance (p/s/cm2/sr) was measured for each mouse. For all the experiments, once mice became moribund in any group, they were all sacrificed, necropsied, and livers were harvested. The number of liver metastases and location of tumor nodules were recorded. Tumor tissue was either fixed in formalin for paraffin embedding, frozen in optimal cutting temperature (OCT) media to prepare frozen slides, or snap-frozen for lysate preparation.

### Protein-coding gene expression by 44 K Agilent array and data analyses

Agilent 44 K two color arrays of the N-BLR siRNA transfected clones (#3-1 and #4-7) were performed in duplicate, along with RNA from empty vector transfected clone in each array. The analysis was performed in R using the functions of the LIMMA library. Probe intensities were background corrected, log2 transformed, log-normalized within arrays, and quantile-normalized between arrays. Finally, replicate spots were averaged. A linear model was fitted to each gene and empirical Bayes methods were used to obtain the statistics. Genes were considered statistically significant if their *p* value was less than 0.001. This stringent significance threshold was used to limit the number of false-positive findings.

### Array design and experiments

The MDACC Expression Bioarrays are transcriptional profiling products designed to monitor the expression of miRNAs and other ncRNAs. The arrays utilize nucleic acid hybridization of a 52 nt biotin-labeled cDNA target with DNA oligonucleotide probes attached to a gel matrix. The biotin-labeled cDNA targets are prepared by a simple reverse transcription into first strand cDNA. Total RNA is primed for reverse transcription by a random octamer conjugated with two biotins and a 52 nt long poly-A tail. This procedure results in an equal copy number of biotin cDNA targets to the ncRNA templates. The chip MDACCv5 array version (Array Express Accession Number A-MEXP-1738) includes 2354 probes for pyknon sequences (each in duplicate).

### Pyknon array data analysis

The MDACCv5 arrays were processed according to a previously optimized processing pipeline for Agilent miRNA arrays [50]. Raw image files were imported into Matlab and not-annotated probes were removed. The median foreground signal from each array was normalized using robust multichip averaging (RMA). Background correction was done with the Limma package in R. Duplicate probes were averaged and the data were standardized before multivariate statistical analysis. Hierarchical clustering, PCA, Partial Least Squares-Discriminant Analysis, and correlation computations were carried out in R.

### Statistical analysis

The relationship between non-dichotomized expression of pyknon-containing regions and cancer (cancer versus normal) was assessed using the independent sample t-test. We also used the Mann–Whitney U-test to compare values between groups of samples. For multiple analyses, a multivariate logistic regression model was used to assess the effect of pyknon-containing region expression on CRC stage and metastasis. We identified pyknon-containing regions whose dichotomized expression was significantly related to the cancer stage and metastasis. We defined correlation and significance levels for pyknon-containing region expressions and clinical factors based on a univariate Cox proportional hazard regression model. For multivariate analysis, a full Cox proportional hazards model was initially fitted that included variables with a *p* value < 0.25 (first CRC cohort), or all of covariates (second CRC cohort) in the univariate analysis. Statistical analysis was performed using the SPSS software (SPSS for Windows Version 16.0, SPSS Inc., Chicago, IL, USA). All tests were two-sided and an effect was considered to be statistically significant at *p* < 0.05.

## Additional files

Additional file 1: Table S1. Genetic information about the pyknon-containing regions. (XLSX 12 kb)

Additional file 2: Table S2. Primer sequences used for qRT-PCR, RACE, siRNAs, and vector construction in this study. (XLSX 11 kb)

Additional file 3: Supplementary Figures S1–21. (DOC 61 mb)

Additional file 4: Table S3. Clinico-pathological characteristics of first CRC patient cohort. (XLSX 9 kb)

Additional file 5: Table S4a. Univariate and multivariate logistic regression of the first CRC patient cohort. (XLSX 9 kb)

Additional file 6: Table S4b. Univariate and multivariate analysis of the disease-specific survival for the first CRC patient cohort. (XLSX 9 kb)

Additional file 7: Table S5. Clinico-pathological features of the second CRC patient cohort. (XLSX 10 kb)

Additional file 8: Table S6a. Univariate and multivariate logistic regression of the second CRC patient cohort. (XLSX 9 kb)

Additional file 9: Table S6b. Univariate and multivariate analysis of the overall survival for the second CRC patient cohort. (XLSX 10 kb)

Additional file 10: Table S7. Clinico-pathological characteristics of the metastatic colorectal carcinoma from the third CRC patient cohort. (XLSX 9 kb)

Additional file 11: Table S8. Clinico-pathological features of the fourth CRC patient cohort. (XLSX 10 kb)

Additional file 12: Table S9. The multivariate Cox proportional hazards model of the fourth CRC patient cohort. (XLSX 19 kb)

## Abbreviations

CRC: Colorectal cancer
EMT: Epithelial to-mesenchymal transition
ISH: In situ hybridization
lncRNA: Long non-coding RNA
LOH: Loss of heterozygosity
miRNA: miR: MicroRNA
mRNA: Messenger RNA
MSI-H: Microsatellite instable high
MSS: Microsatellite stable
qRT-PCR: Quantitative (real-time) reverse transcription-polymerase chain reaction
RACE: Rapid amplification of cDNA ends
TSS: Transcription start site

## References

[1] Carninci P, Kasukawa T, Katayama S, Gough J, Frith MC, Maeda N (2005). The transcriptional landscape of the mammalian genome. Science..

[2] Cheng J, Kapranov P, Drenkow J, Dike S, Brubaker S, Patel S (2005). Transcriptional maps of 10 human chromosomes at 5-nucleotide resolution. Science..

[3] Shigematsu M, Honda S, Kirino Y (2014). Transfer RNA as a source of small functional RNA. J Mol Biol Mol Imaging..

[4] Honda S, Loher P, Shigematsu M, Palazzo JP, Suzuki R, Imoto I (2015). Sex hormone-dependent tRNA halves enhance cell proliferation in breast and prostate cancers. Proc Natl Acad Sci U S A..

[5] Telonis AG, Loher P, Honda S, Jing Y, Palazzo J, Kirino Y (2015). Dissecting tRNA-derived fragment complexities using personalized transcriptomes reveals novel fragment classes and unexpected dependencies. Oncotarget..

[6] Cech TR, Steitz JA (2014). The noncoding RNA revolution-trashing old rules to forge new ones. Cell..

[7] Ling H, Vincent K, Pichler M, Fodde R, Berindan-Neagoe I, Slack FJ (2015). Junk DNA and the long non-coding RNA twist in cancer genetics. Oncogene..

[8] Rigoutsos I (2009). New tricks for animal microRNAS: targeting of amino acid coding regions at conserved and nonconserved sites. Cancer Res..

[9] Bartel DP (2009). MicroRNAs: target recognition and regulatory functions. Cell..

[10] Berindan-Neagoe I, Monroig Pdel C, Pasculli B, Calin GA (2014). MicroRNAome genome: a treasure for cancer diagnosis and therapy. CA Cancer J Clin..

[11] Tay Y, Rinn J, Pandolfi PP (2014). The multilayered complexity of ceRNA crosstalk and competition. Nature..

[12] Djebali S, Davis CA, Merkel A, Dobin A, Lassmann T, Mortazavi A (2012). Landscape of transcription in human cells. Nature..

[13] Gupta RA, Shah N, Wang KC, Kim J, Horlings HM, Wong DJ (2010). Long non-coding RNA HOTAIR reprograms chromatin state to promote cancer metastasis. Nature..

[14] Kogo R, Shimamura T, Mimori K, Kawahara K, Imoto S, Sudo T (2011). Long noncoding RNA HOTAIR regulates polycomb-dependent chromatin modification and is associated with poor prognosis in colorectal cancers. Cancer Res..

[15] Lee JT, Lu N (1999). Targeted mutagenesis of Tsix leads to nonrandom X inactivation. Cell..

[16] Ogawa Y, Sun BK, Lee JT (2008). Intersection of the RNA interference and X-inactivation pathways. Science (New York, NY).

[17] Tian D, Sun S, Lee JT (2010). The long noncoding RNA, Jpx, is a molecular switch for X chromosome inactivation. Semin Fetal Neonatal Med..

[18] Pandey RR, Mondal T, Mohammad F, Enroth S, Redrup L, Komorowski J (2008). Kcnq1ot1 antisense noncoding RNA mediates lineage-specific transcriptional silencing through chromatin-level regulation. Mol Cell..

[19] Chen L-L, Carmichael GG (2009). Altered nuclear retention of mRNAs containing inverted repeats in human embryonic stem cells: functional role of a nuclear noncoding RNA. Mol Cell..

[20] Yang L, Lin C, Liu W, Zhang J, Ohgi KA, Grinstein JD (2011). ncRNA- and Pc2 methylation-dependent gene relocation between nuclear structures mediates gene activation programs. Cell.

[21] Shah N, Sukumar S (2010). The Hox genes and their roles in oncogenesis. Nat Rev Cancer..

[22] Wang KC, Yang YW, Liu B, Sanyal A, Corces-Zimmerman R, Chen Y (2011). A long noncoding RNA maintains active chromatin to coordinate homeotic gene expression. Nature..

[23] Maass PG, Rump A, Schulz H, Stricker S, Schulze L, Platzer K (2012). A misplaced lncRNA causes brachydactyly in humans. J Clin Investig..

[24] Bischof JM, Stewart CL, Wevrick R (2007). Inactivation of the mouse Magel2 gene results in growth abnormalities similar to Prader-Willi syndrome. Hum Mol Genet..

[25] Khaitan D, Dinger ME, Mazar J, Crawford J, Smith MA, Mattick JS (2011). The melanoma-upregulated long noncoding RNA SPRY4-IT1 modulates apoptosis and invasion. Cancer Res..

[26] Ling H, Spizzo R, Atlasi Y, Nicoloso M, Shimizu M, Redis RS (2013). CCAT2, a novel noncoding RNA mapping to 8q24, underlies metastatic progression and chromosomal instability in colon cancer. Genome Res..

[27] Ferdin J, Nishida N, Wu X, Nicoloso MS, Shah MY, Devlin C (2013). HINCUTs in cancer: hypoxia-induced noncoding ultraconserved transcripts. Cell Death Differ..

[28] Prensner JR, Iyer MK, Balbin OA, Dhanasekaran SM, Cao Q, Brenner JC (2011). Transcriptome sequencing across a prostate cancer cohort identifies PCAT-1, an unannotated lincRNA implicated in disease progression. Nat Biotechnol..

[29] Rigoutsos I, Floratos A (1998). Combinatorial pattern discovery in biological sequences: The TEIRESIAS algorithm. Bioinformatics..

[30] Rigoutsos I, Huynh T, Miranda K, Tsirigos A, McHardy A, Platt D (2006). Short blocks from the noncoding parts of the human genome have instances within nearly all known genes and relate to biological processes. Proc Natl Acad Sci U S A..

[31] Rigoutsos I, Furnari F (2010). Gene-expression forum: Decoy for microRNAs. Nature..

[32] Tsirigos A, Rigoutsos I (2008). Human and mouse introns are linked to the same processes and functions through each genome’s most frequent non-conserved motifs. Nucleic Acids Res..

[33] Robine N, Lau NC, Balla S, Jin Z, Okamura K, Kuramochi-Miyagawa S (2009). A broadly conserved pathway generates 3′UTR-directed primary piRNAs. Curr Biol..

[34] Saito K, Inagaki S, Mituyama T, Kawamura Y, Ono Y, Sakota E (2009). A regulatory circuit for piwi by the large Maf gene traffic jam in Drosophila. Nature..

[35] Feng J, Naiman DQ, Cooper B (2009). Coding DNA repeated throughout intergenic regions of the Arabidopsis thaliana genome: evolutionary footprints of RNA silencing. Mol Biosyst..

[36] Di Ruscio A, Ebralidze AK, Benoukraf T, Amabile G, Goff LA, Terragni J (2013). DNMT1-interacting RNAs block gene-specific DNA methylation. Nature..

[37] Calin GA, Sevignani C, Dumitru CD, Hyslop T, Noch E, Yendamuri S (2004). Human microRNA genes are frequently located at fragile sites and genomic regions involved in cancers. Proc Natl Acad Sci U S A..

[38] Clemson CM, Hutchinson JN, Sara SA, Ensminger AW, Fox AH, Chess A (2009). An architectural role for a nuclear noncoding RNA: NEAT1 RNA is essential for the structure of paraspeckles. Mol Cell..

[39] Orom UA, Derrien T, Beringer M, Gumireddy K, Gardini A, Bussotti G (2010). Long noncoding RNAs with enhancer-like function in human cells. Cell..

[40] Hamazaki N, Uesaka M, Nakashima K, Agata K, Imamura T (2015). Gene activation-associated long noncoding RNAs function in mouse preimplantation development. Development..

[41] Marin-Bejar O, Marchese FP, Athie A, Sanchez Y, Gonzalez J, Segura V (2013). Pint lincRNA connects the p53 pathway with epigenetic silencing by the Polycomb repressive complex 2. Genome Biol..

[42] Satow R, Hirano T, Batori R, Nakamura T, Murayama Y, Fukami K (2014). Phospholipase Cdelta1 induces E-cadherin expression and suppresses malignancy in colorectal cancer cells. Proc Natl Acad Sci U S A..

[43] Puisieux A, Brabletz T, Caramel J (2014). Oncogenic roles of EMT-inducing transcription factors. Nat Cell Biol..

[44] Gregory PA, Bert AG, Paterson EL, Barry SC, Tsykin A, Farshid G (2008). The miR-200 family and miR-205 regulate epithelial to mesenchymal transition by targeting ZEB1 and SIP1. Nat Cell Biol..

[45] Miranda KC, Huynh T, Tay Y, Ang Y-S, Tam W-L, Thomson AM (2006). A pattern-based method for the identification of MicroRNA binding sites and their corresponding heteroduplexes. Cell..

[46] Belgardt BF, Ahmed K, Spranger M, Latreille M, Denzler R, Kondratiuk N (2015). The microRNA-200 family regulates pancreatic beta cell survival in type 2 diabetes. Nat Med..

[47] Zhang Y, Talmon G, Wang J (2015). MicroRNA-587 antagonizes 5-FU-induced apoptosis and confers drug resistance by regulating PPP2R1B expression in colorectal cancer. Cell Death Dis..

[48] Ciccone M, Ferrajoli A, Keating MJ, Calin GA (2014). SnapShot: chronic lymphocytic leukemia. Cancer Cell..

[49] Hur K, Toiyama Y, Takahashi M, Balaguer F, Nagasaka T, Koike J (2013). MicroRNA-200c modulates epithelial-to-mesenchymal transition (EMT) in human colorectal cancer metastasis. Gut..

[50] Yuan JH, Yang F, Wang F, Ma JZ, Guo YJ, et al. A long noncoding RNA activated by TGF-β promotes the invasion-metastasis cascade in hepatocellular carcinoma. Cancer Cell. 2014;25:666-81. 10.1016/j.ccr.2014.03.010 24768205

[51] Rigoutsos I (2010). Short RNAs: how big is this iceberg?. Curr Biol..

[52] Tay SK, Blythe J, Lipovich L (2009). Global discovery of primate-specific genes in the human genome. Proc Natl Acad Sci U S A..

[53] Mestdagh P, Fredlund E, Pattyn F, Rihani A, Van Maerken T, Vermeulen J (2010). An integrative genomics screen uncovers ncRNA T-UCR functions in neuroblastoma tumours. Oncogene..

[54] Ling H, Girnita L, Buda O, Calin GA. Non-coding RNAs: the cancer genome dark matter that matters! Clin Chem Lab Med. 2017;55:705-714. 10.1515/cclm-2016-0740 27988500

[55] Van Dyke T, Jacks T (2002). Cancer modeling in the modern era: progress and challenges. Cell..

[56] Bullock MD, Silva AM, Kanlikilicer-Unaldi P, Filant J, Rashed MH, Sood AK (2015). Exosomal non-coding RNAs: diagnostic, prognostic and therapeutic applications in Cancer. Non-Coding RNA..

[57] Calin GA, Liu C-G, Ferracin M, Hyslop T, Spizzo R, Sevignani C (2007). Ultraconserved regions encoding ncRNAs are altered in human leukemias and carcinomas. Cancer Cell..

[58] Almeida MI, Nicoloso MS, Zeng L, Ivan C, Spizzo R, Gafa R (2012). Strand-specific miR-28-5p and miR-28-3p have distinct effects in colorectal cancer cells. Gastroenterology.

[59] Mani SA, Guo W, Liao MJ, Eaton EN, Ayyanan A, Zhou AY (2008). The epithelial-mesenchymal transition generates cells with properties of stem cells. Cell..

[60] Ohtsuka M, Ling H, Ivan C, Pichler M, Matsushita D, Goblirsch M (2016). H19 noncoding RNA, an independent prognostic factor, regulates essential Rb-E2F and CDK8-beta-Catenin signaling in colorectal cancer. EBioMedicine..

[61] Liu S, Anfossi S, Qiu B, Zheng Y, Cai M, Fu J (2017). Prognostic factors for locoregional recurrence in patients with thoracic esophageal squamous cell carcinoma treated with radical two-field lymph node dissection: results from long-term follow-up. Ann Surg Oncol..

[62] Huang W, Hennrick K, Drew S (2013). A colorful future of quantitative pathology: validation of Vectra technology using chromogenic multiplexed immunohistochemistry and prostate tissue microarrays. Hum Pathol..

