# Supplementary material for: N-BLR, a primate-specific non-coding transcript leads to colorectal cancer invasion and migration
Source: Genome Biol. 2017 May 24;18:98. doi: 10.1186/s13059-017-1224-0 (PMC5442648; doi:10.1186/s13059-017-1224-0)
Supplement: Supplementary file 3 — Supplementary Figures S1–21. (DOC 61 mb) [file 13059_2017_1224_MOESM3_ESM.doc]

**SUPPLEMENTARY FIGURES**

**N-BLR, a primate-specific non-coding transcript leads to colorectal cancer invasion and migration**

Isidore Rigoutsos, Sang Kil Lee, Su Youn Nam, Simone Anfossi, Barbara Pasculli, Martin Pichler, Yi Jing, Cristian Rodriguez-Aguayo, Aristeidis G. Telonis, Simona Rossi, Cristina Ivan, Tina Catela Ivkovic, Linda Fabris, Peter M. Clark, Hui Ling, Masayoshi Shimizu, Roxana S. Redis, Maitri Y. Shah, Xinna Zhang, Yoshinaga Okugawa, Eun Jung Jung, Aristotelis Tsirigos, Li Huang, Jana Ferdin, Roberta Gafà, Riccardo Spizzo, Milena S. Nicoloso, Anurag N. Paranjape, Maryam Shariati, Aida Tiron, Jen Jen Yeh, Raul Teruel-Montoya, Lianchun Xiao, Sonia A. Melo, David Menter, Zhiqin C. Jiang, Elsa R Flores, Massimo Negrini, Ajay Goel, Menashe Bar-Eli, Sendurai A. Mani, Chang Gong Liu, Gabriel Lopez-Berestein, Ioana Berindan-Neagoe, Manel Esteller, Scott Kopetz, Giovanni Lanza, George A. Calin

**CONTENT**

**Additional file 3: Supplementary Figures S1 to S21, pages 2 to 28**

**Additional file 3: Fig. S1**. **Colorectal cancer cell lines exhibit variable expression of pyknon-regions**. Levels for 11 pyknon-regions were measured in 7 colon cancer cell lines (Colo320, SW480, HCT116, LS174, HT-29, Colo205, and SW620). Average over 5 normal colon tissues is included for comparison. Pyk-reg-26 and pyk-reg-27 were not detectable in normal colon tissue. Y-axis values represent ratio of each pyknon-region to U6: ratios were calculated with the 2^-ΔCt^ method using U6 levels for normalization. Data are shown as mean ± stdev and represent experiments performed in duplicate.

**Additional file 3: Fig. S2**. **Expression of pyk-reg-90 (N-BLR) in metastatic CRC samples obtained from Dr. Jen-Jen** **Yeh, University of North Carolina at Chapel Hill**. Solid bold arrow indicates the N-BLR band constantly present in all samples that express N-BLR; dashed arrow indicates a lower band present in some of the samples expressing N-BLR that did not represent a splicing variant according to the Sanger sequencing data, but a conformational variant that is running at different speed.

| 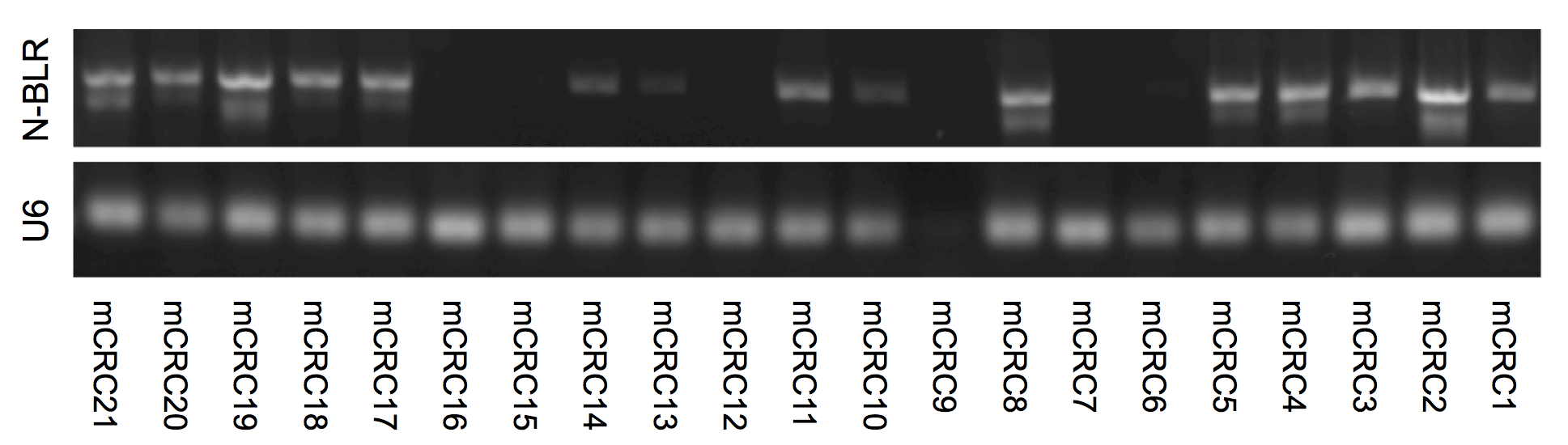 |
| --- |

**Additional file 3: Fig. S3. Genomic characterization of N-BLR locus. (A)** Sequence and annotation of cloned N-BLR. **(B)** UCSC genomic browser presentation of BLAT of cloned N-BLR. Black arrow-tip = the strand polarity of the genes. The black rectangle inside the red rectangle = N-BLR. **(C)** On the left we show the single band amplification of N-BLR in a normal colon sample (C-42), and the Colo320 (320) and HCT116 (116) cancer cells lines. On the right, we show the lack of protein coding potential of N-BLR. We performed *in vitro* transcription-translation assay of the 844-nt mono-exonic transcript and no specific bands were identified. **(D)** The potential coding ability of N-BLR was assessed using two computational tools that evaluate protein-coding potential (http://cpc.cbi.pku.edu.cn/ and http://lilab.research.bcm.edu/cpat/index.php). Both tools confirmed the lack of coding potential ability of N-BLR. We also assessed the KRAS proto-oncogene and HOTAIR lncRNA sequences as positive and negative controls, respectively. **(E)** BLAST results for the N-BLR conservation performed in NCI webpage.

**A**

**N-BLR sequence**

**844 nts / chr3 + 52155868 52156712**

N-BLR clone is in **boldface** upper case

**pyknon-90 is in BOLD BLUE upper case**

**FWD qRTPCR primer REV qRTPCR primer**

**FWD RACE primer REV RACE primer**

**FWD nested RACE primer REV nested RACE primer**

purple means red + cyan

**Sequence flanked by 300 nts on each side *in italics* (i.e. 52155568– 52157012)**

*tatatataaaataaaatttgaaggccaccagcaaccatctgaatagactccctgcttggccagggcactcaaatttaacctgaaagagtggttcaggccatgatgggaagtgggggttggacatgcctcattataccctccagcattaacatcaacaaaaaccttaagtctgataagaaacatttacgatctattttctctgaaacctgctacctggaggcttcatctgcatggtaaaaccttggtctccataaccccttatcttaaccgagatatccctttctactgataataattattt***CAACCAATTGCCAATCAGAATATGTTTAAATCTACCAATGACCTGGAAGTTTCCCACTTCGAGTTGTCTTGTCCTTCCAGATCAAACCAATGTAAATCTTACATGTATTGATTGATGTATTTTGTCTCCCTAAAATGTATAAAACCATGCTGTGCCCAGGCCACTTGGGCACATGTTGTCAGGACCTCCTGAGGCTGTGTCATGGGTGTGTCCTTAACCTTAGCAAAATAAATTTTCTAAGTTGACTGAGACCTGTCTCAGGTATTTCGGGTTGACAGTTTGGCAACCACGAAAGGATTCTGAGTGGAGGTGCCCCTGACCTTTGACAGATCTCCTATCGGTGCTTGGTACCAGCTTGAGCTATCTTTATGGCTCAAATCTACAGGACAATTTGCTAAGGCCTGGGAGCCCCCCTCCACAAAATTCCTGATCTTCCCAAATTTGGTTAACATCTAAAGTTTATTTTACTGTACAACTCCTTTTTCTGGAGTTTTACTTGTTTCCAACAAGGAAGGCAGGTTTTCCTGCTTCCCTGACGATGGAAGGCAGGTAACTCCTTTCTGGAGTTTGAGCTCGCTTCCAACAGAGAAGGAAAGTTTGAGTTTTCCCTACTTCTGGGATGGTAGAGAGCAGTCTTTAGCCTAAGACCTGTTCTTAGGTAAGTAGCCGAATTGGGGATTTTCTTGGCTGAAGTTAAGGTTAACAACCAGCTGGTCTTAATTTTCCTTACCATTAGAGTGCTCAGTAATCATATAAATTGTACGATCAAGCCCGGCA**TGGTGGCTCACGCCTGTAAT**CCCAGCACTTTGGGAAGCCAAGGCGGGTGGATCACGAGGTCAGGACAT*cgcagaccatcctggctaacacggtgaaaccccgtctctac****taaaaatacaaaaaattacagggcgtggtggctggcgcctgtagtcccagctactcgggaggctgaggcaggagaatggcatgaacccgggaggcggagcttgcagtgagcctagatcaagcccctgcactccagcctgggcgacagggcgagactccgtctcaaaaaaaaataaaataaataaaataaataaacaaattgtgcgatcactttttttttttttgcttaactgttttttgttgttgtttgtttctgtttt*

**
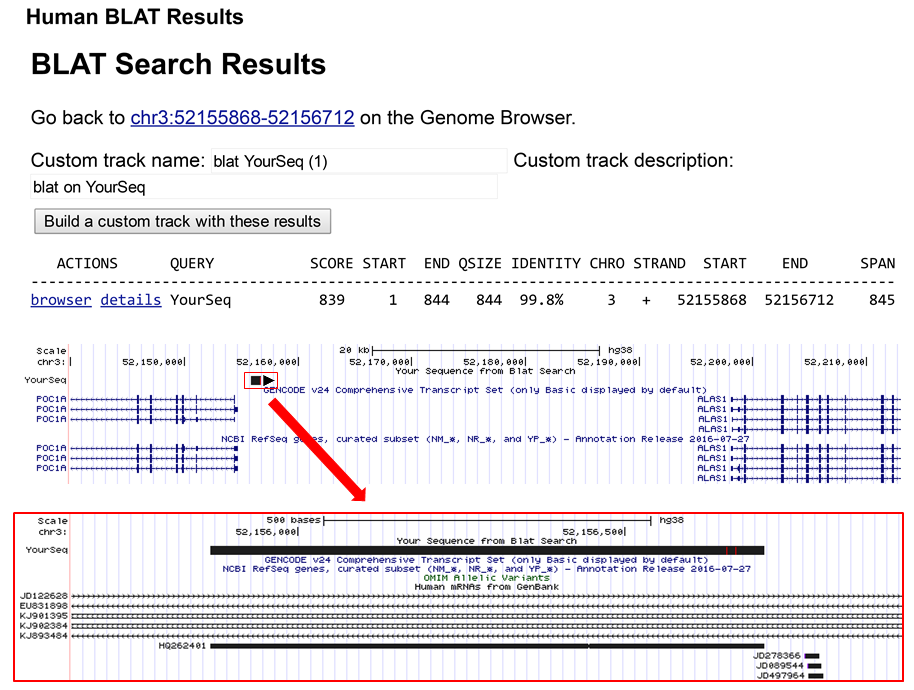
**

**B**

**Additional file 3: Fig. S3 (Continued).**


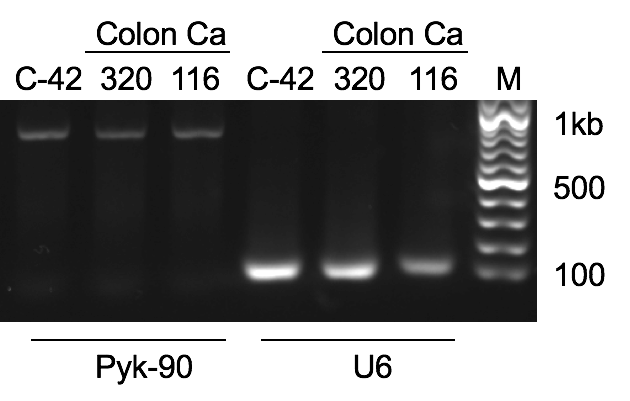


N-BLR

225

150

102

76

52

38

31

24

17

12

Luciferase (61 kD)

Globin (10-15 kD)


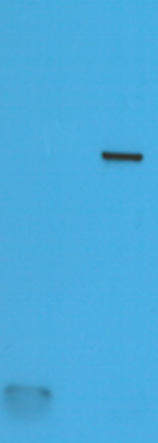


Luciferase

**C**


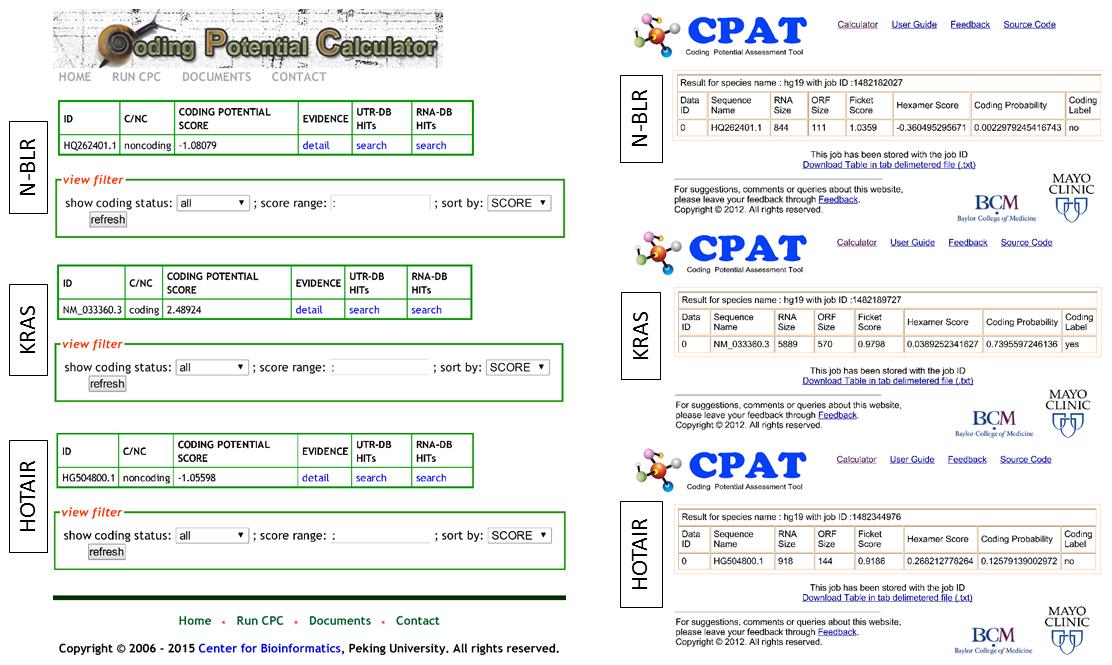
**D**

**Additional file 3: Fig. S3 (Continued).**

**E**

**Additional file 3: Fig. S4**. **Preferential sense transcription to N-BLR**. **(A)** We used sense/antisense specific qRT-PCR and determined that expression sense to the pyk-reg-90 segment of N-BLR (shown by the black bar/arrow in the graph and illustration) is much more abundant than transcription antisense to it (white bar in graph and dot-arrow in illustration). Data are shown as mean ± stdev and represent experiments performed in duplicate. **(B)** Expression of sense (black bar/arrow in graph and illustration) and antisense strand (white bar in graph and dot-arrow in illustration) in the vicinity of pyk-reg-90 (N-BLR) was measured by sense/antisense specific qRT-PCR. The thick arrow indicates the strand sense to pyk-reg-90.

**B**


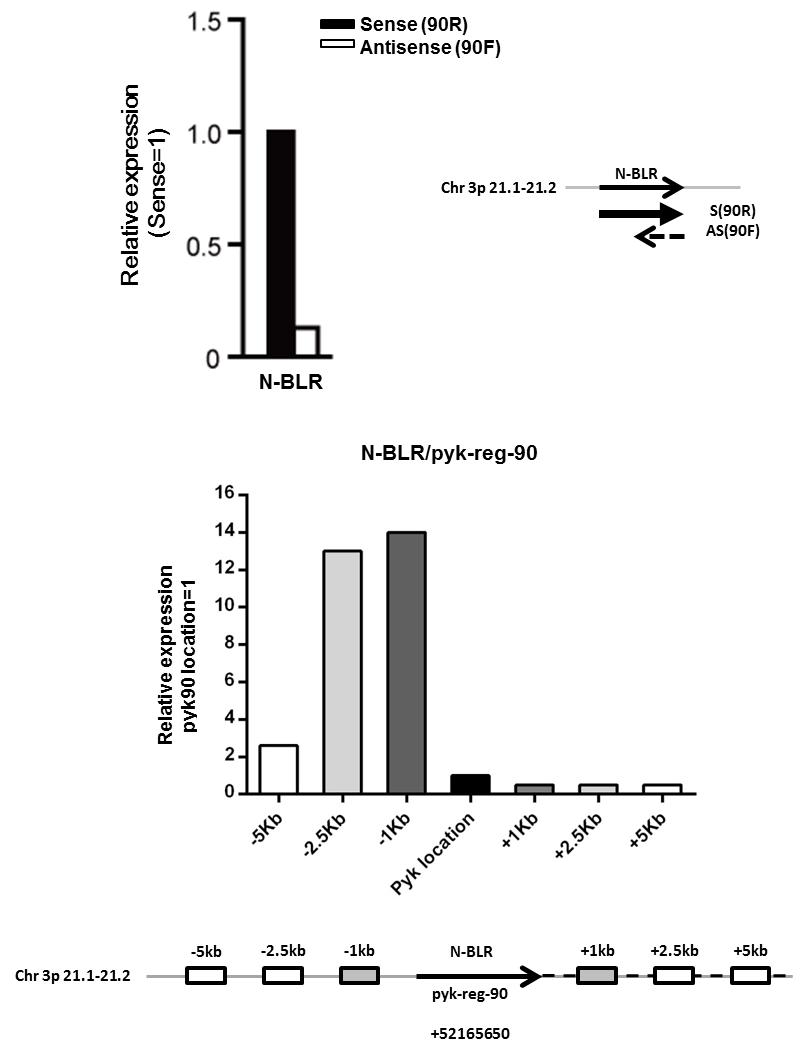

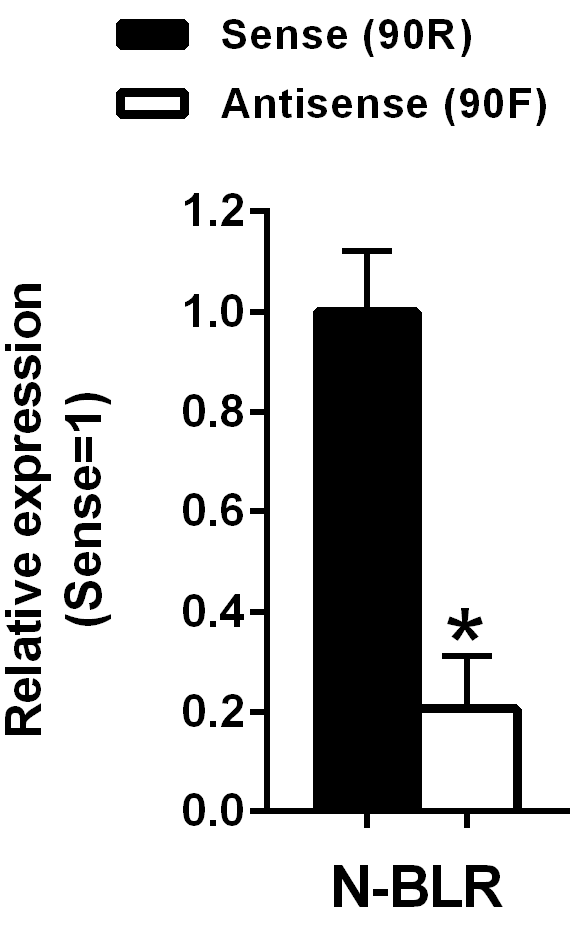


**A**

**pyk-reg-90 (N-BLR)**

**Additional file 3: Fig. S5. *In Situ Hybridization* (ISH) of tissue microarray of colon and N-BLR intracellular localization. (A)** Details of the tissue microarray are presented. **(B)** The expression levels of N-BLR in benign and colitis tissues are shown at increasing magnification (5X, 20X, and 60X). H&E staining was preformed to display the morphology of the tissue. Each H&E image corresponds to the same area of the ISH images for comparison. **(C)** Predominant cytoplasmic location of N-BLR by ISH in TMA samples. The H&E staining and ISH for N-BLR were done on serial sections, therefore perfect overlapping of tissue morphology might not occur between the two images that show the same tissue area. **(D)** Cytoplasmic and nuclear extracts from two cell lines, HCT116 and SW480 were used to quantify N-BLR and the two marker transcripts, U6 (nuclear compartment) and GAPDH (cytoplasmic compartment) by qRT-PCR. Data are shown as mean ± stdev and represent experiments performed in duplicate.

**B**

**A**


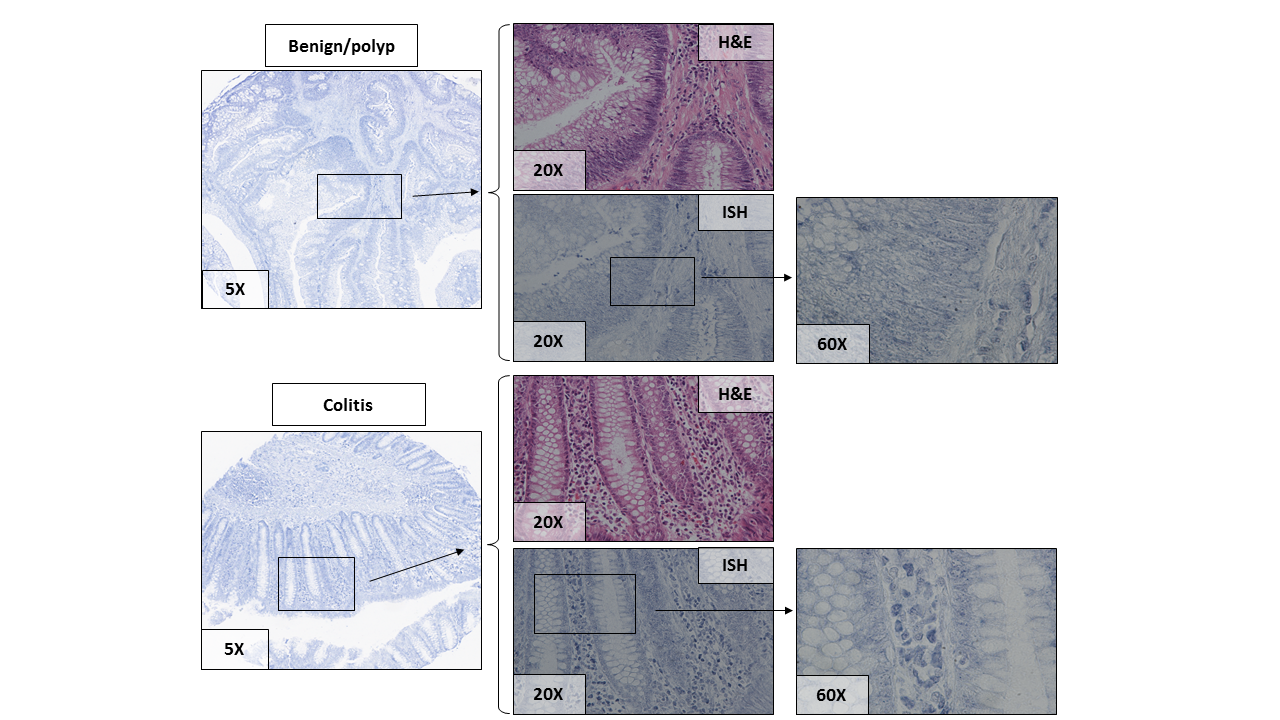

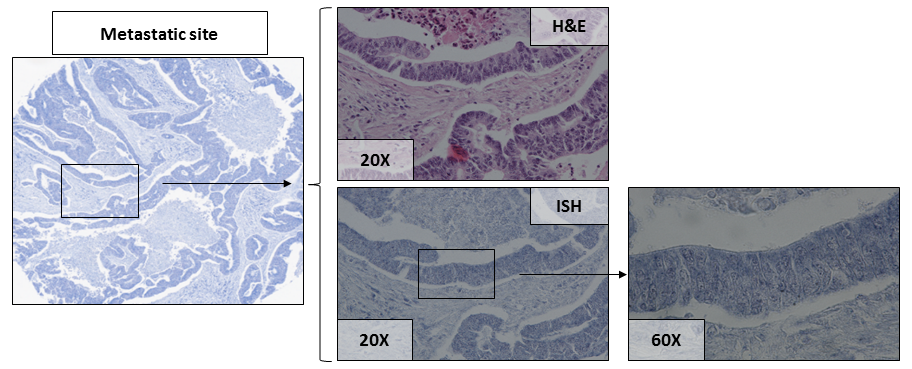

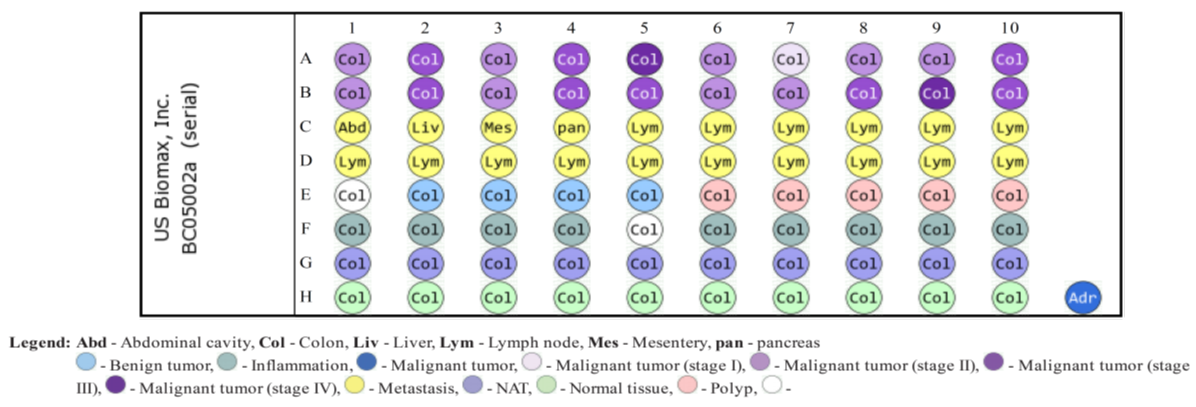


**Additional file 3: Fig. S5 (Continued).**

**C**

**
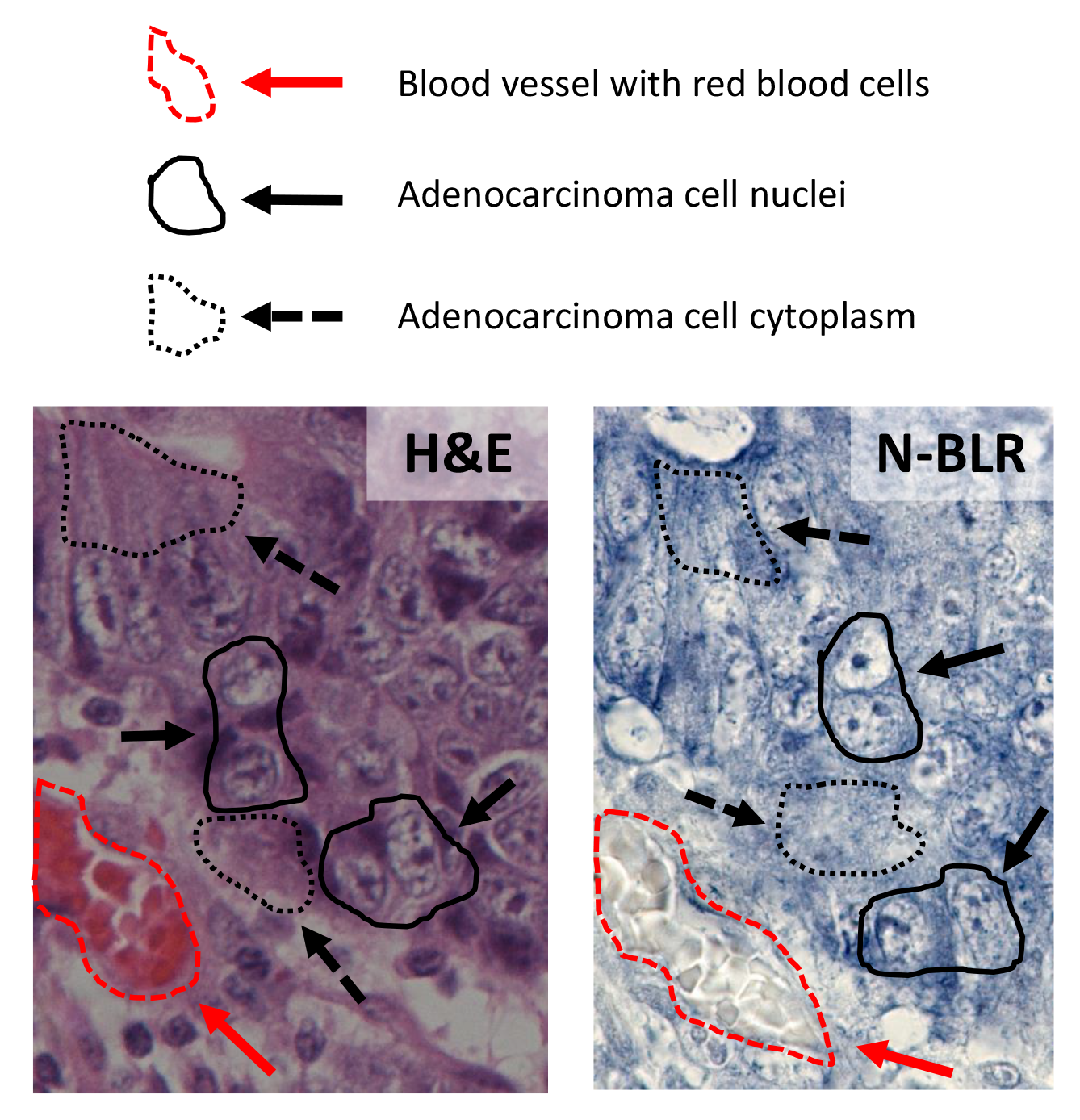
**

**D**


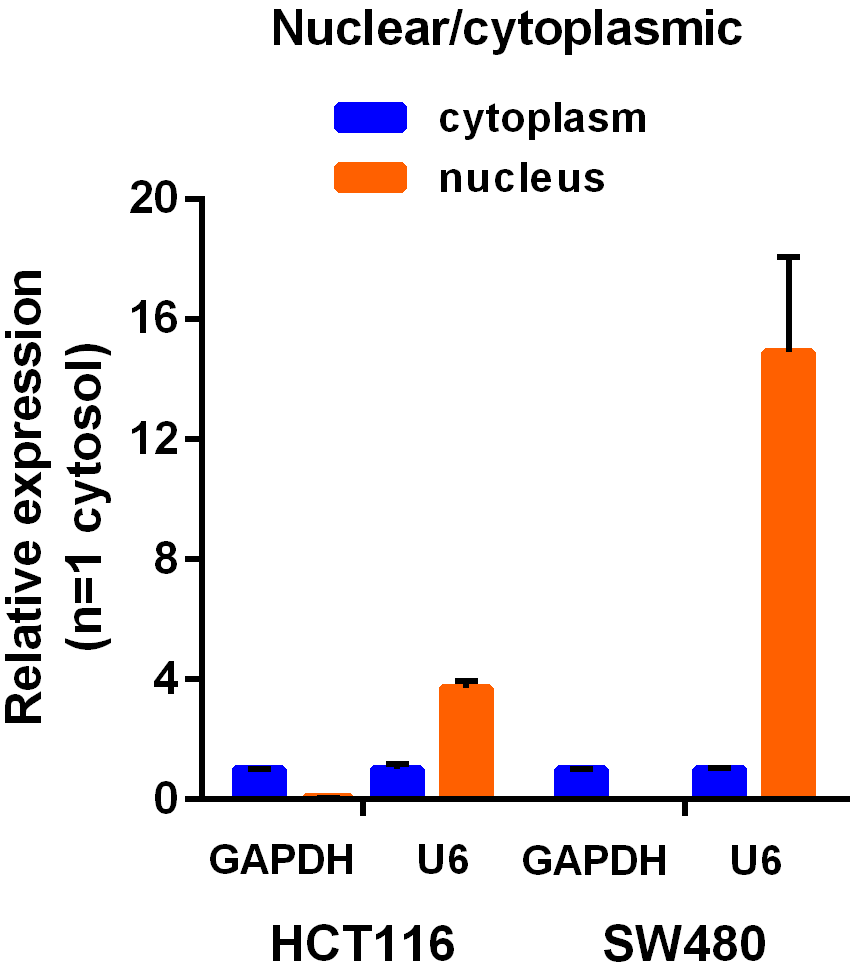

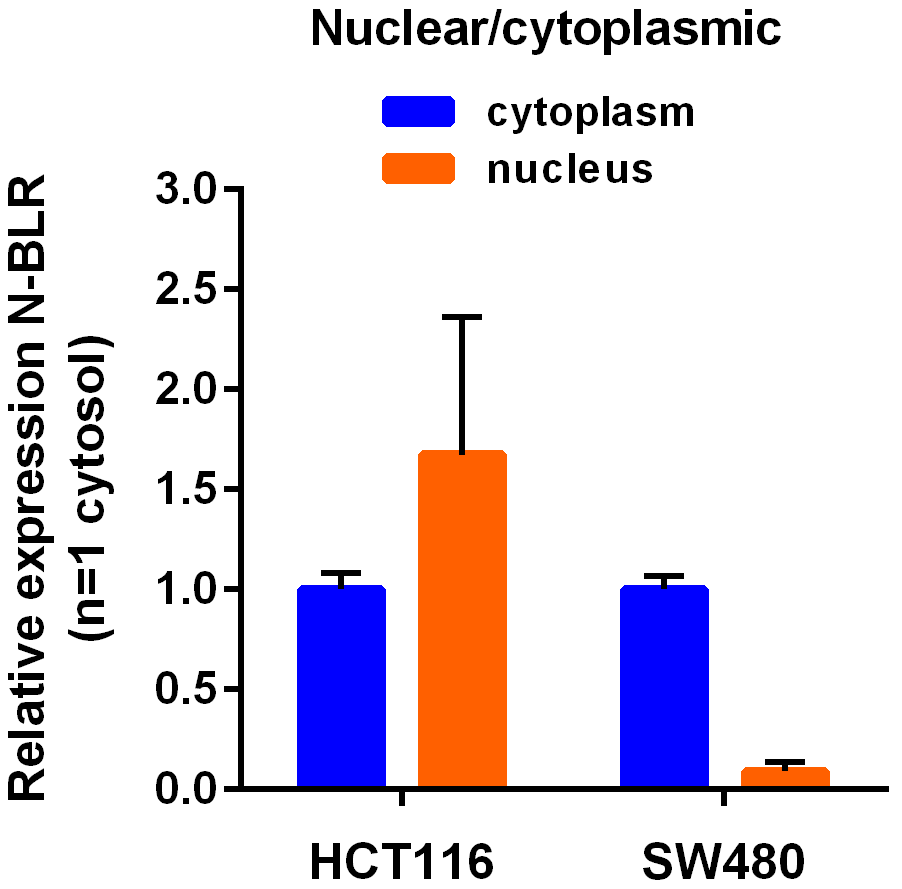


**Additional file 3: Fig. S6. SiRNA-induced inhibition of N-BLR in Colo320 cells. (A)** We designed and experimented with 4 different siRNA sequences at a concentration of 100 nM. After 48 h, expression of N-BLR was determined by qRT-PCR. After testing the silencing effects, a siRNA pool was formed using the two most effective siRNAs. As reference value we used a siRNA control from Dharmacon (scrambled) and cells treated with only lipofectamine (Null). **(B)** We tested dose-dependency on siRNA pool with concentrations ranging from 50 to 300 nM for 48 h. Y-axis labels represent relative expression of N-BLR to scrambled transfection in Colo320. The figures show the average and standard deviation of two independent experiments, each performed in triplicate. **(C)** Expression levels of N-BLR at 96 h after transfection with 100 nM siRNA pool. **(D)** N-BLR´s effect on cell number was measured after transfection with specific N-BLR siRNA1 and N-BLR siRNA3 as well as with the pool (consisting of N-BLR siRNA1 and siRNA3, 100 nM) and scrambled RNA (Ctr, 100 nM) in Colo320 cells. **(E)** Expression levels of miR-200c-3p and XIAP mRNA after 96 h transfection with N-BLR siRNA1+3. **(F)** and **(G)** Colo320 and SW620 cells were synchronized by serum starvation (0.1% FBS) for 48 h at 37°C. Then, cells were transfected and cell cycle was analyzed 48 and 96 h after transfection. Y‑axis: percentage of the total cells that were treated with 100 nM of siRNA pool against N-BLR (black columns) and 100 nM of scrambled control (white columns) in the four cell cycle phases (sub-G1, G1, S, and G2). (Student´s t-test; **p*<0.05; ***p*<0.01; ****p*<0.001; *****p*<0.0001). Data shown represent the mean and standard deviation of two independent experiments performed each time in triplicate.


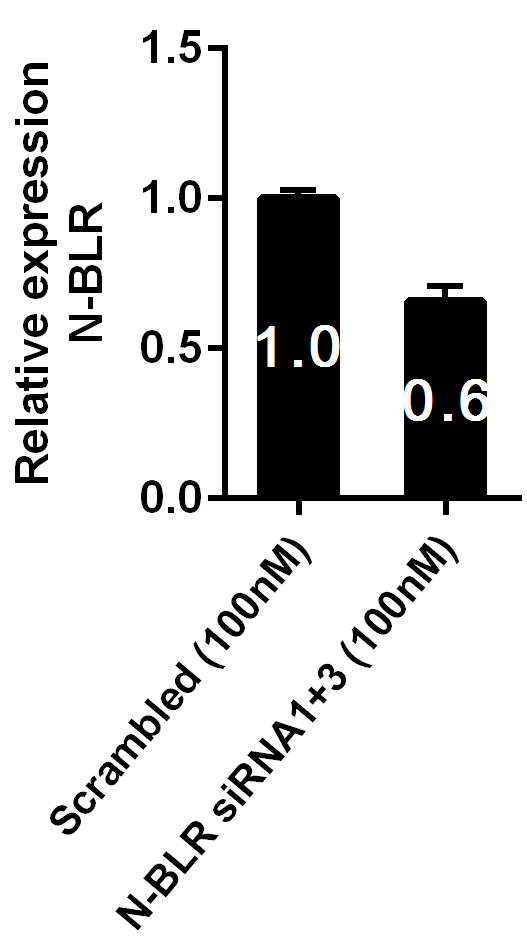

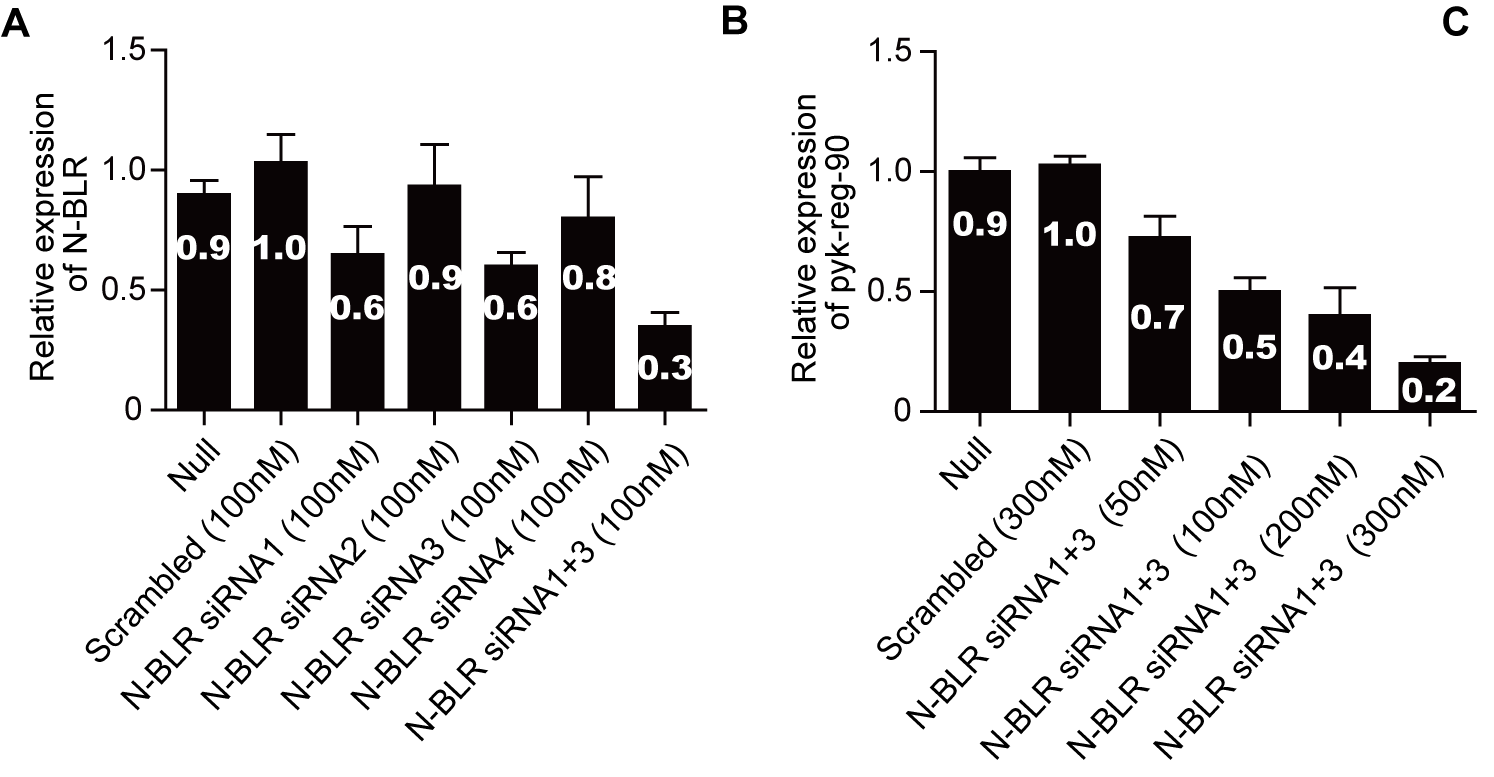

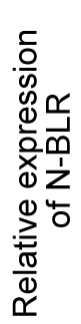


**A**

**B**

**C**

**
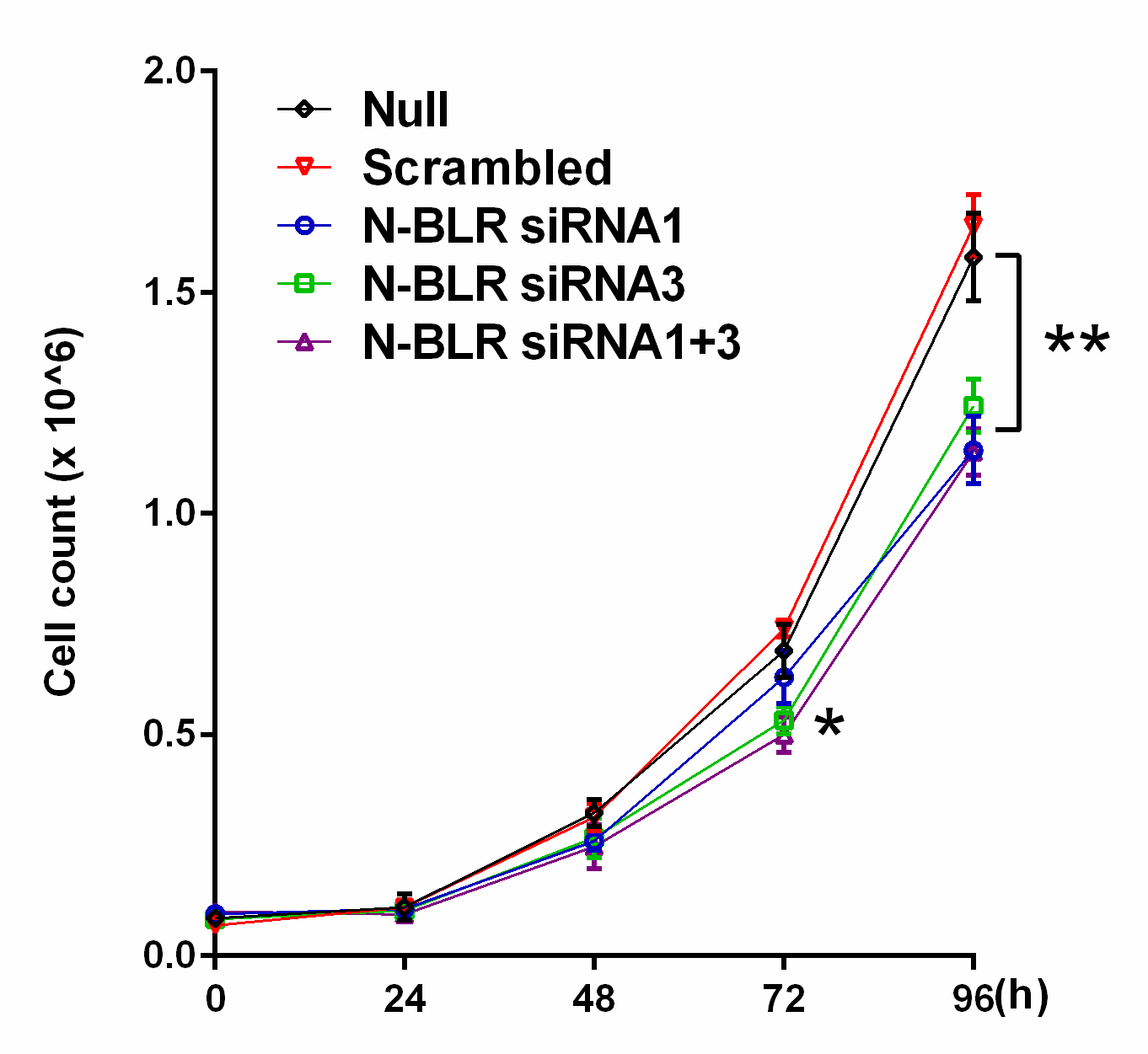
**

**D**

**Additional file 3: Fig. S6 (Continued).**

**E**


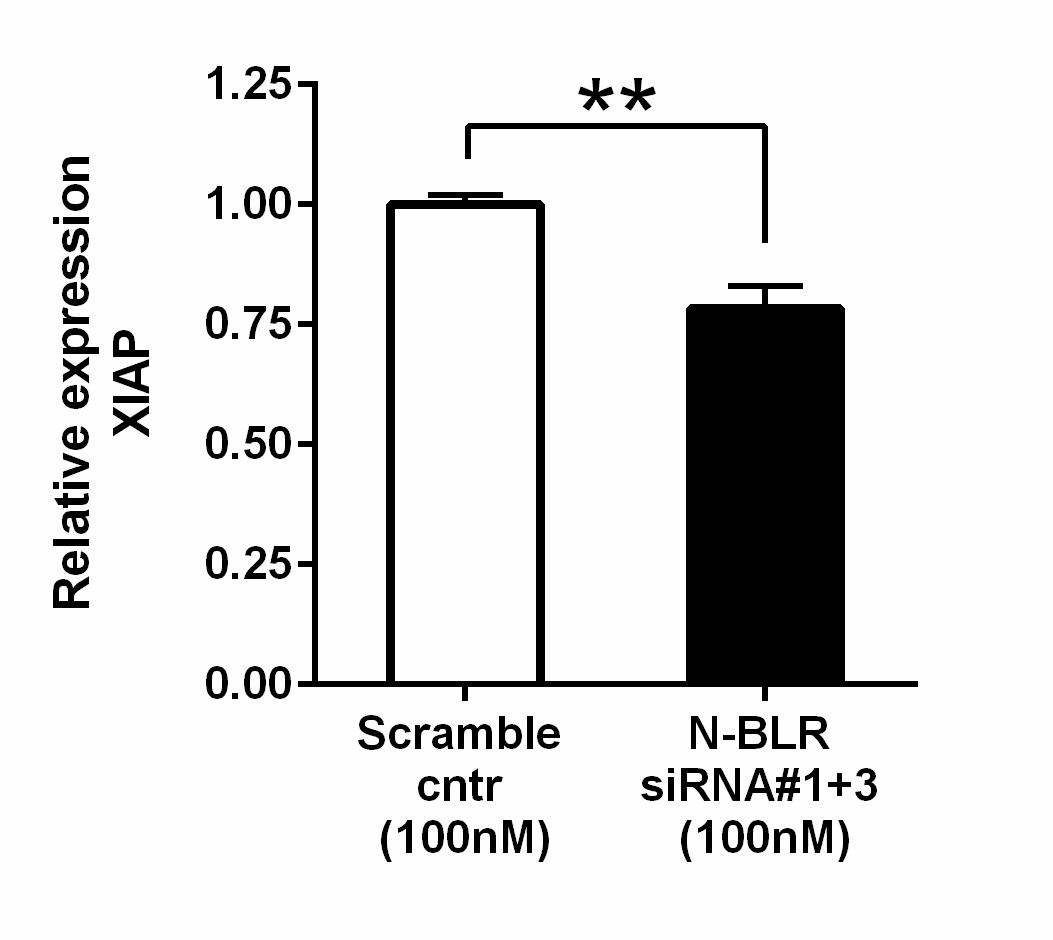

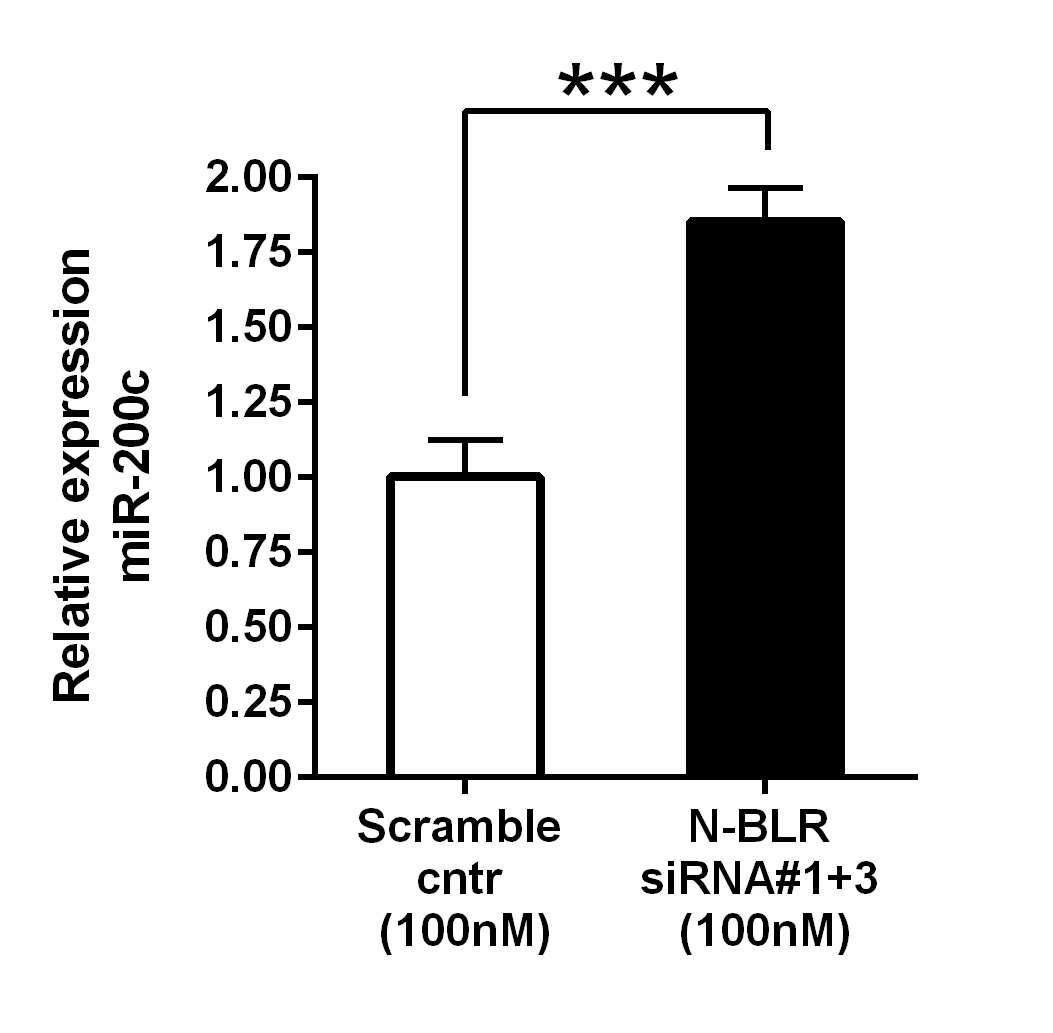


**F**

**G**


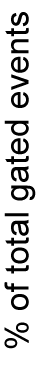

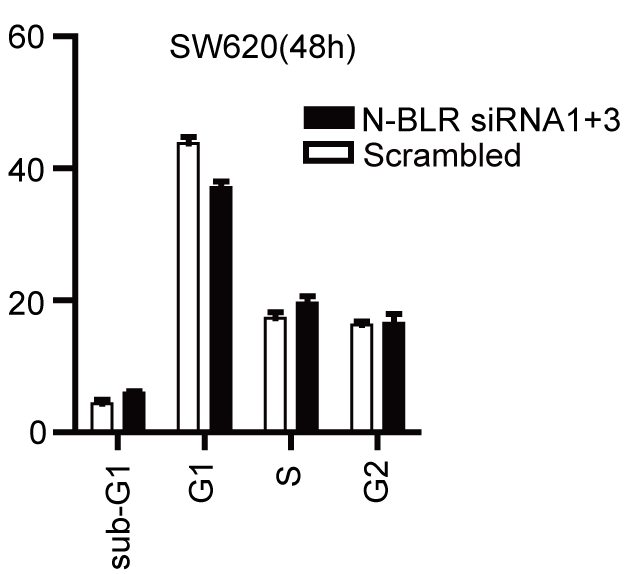

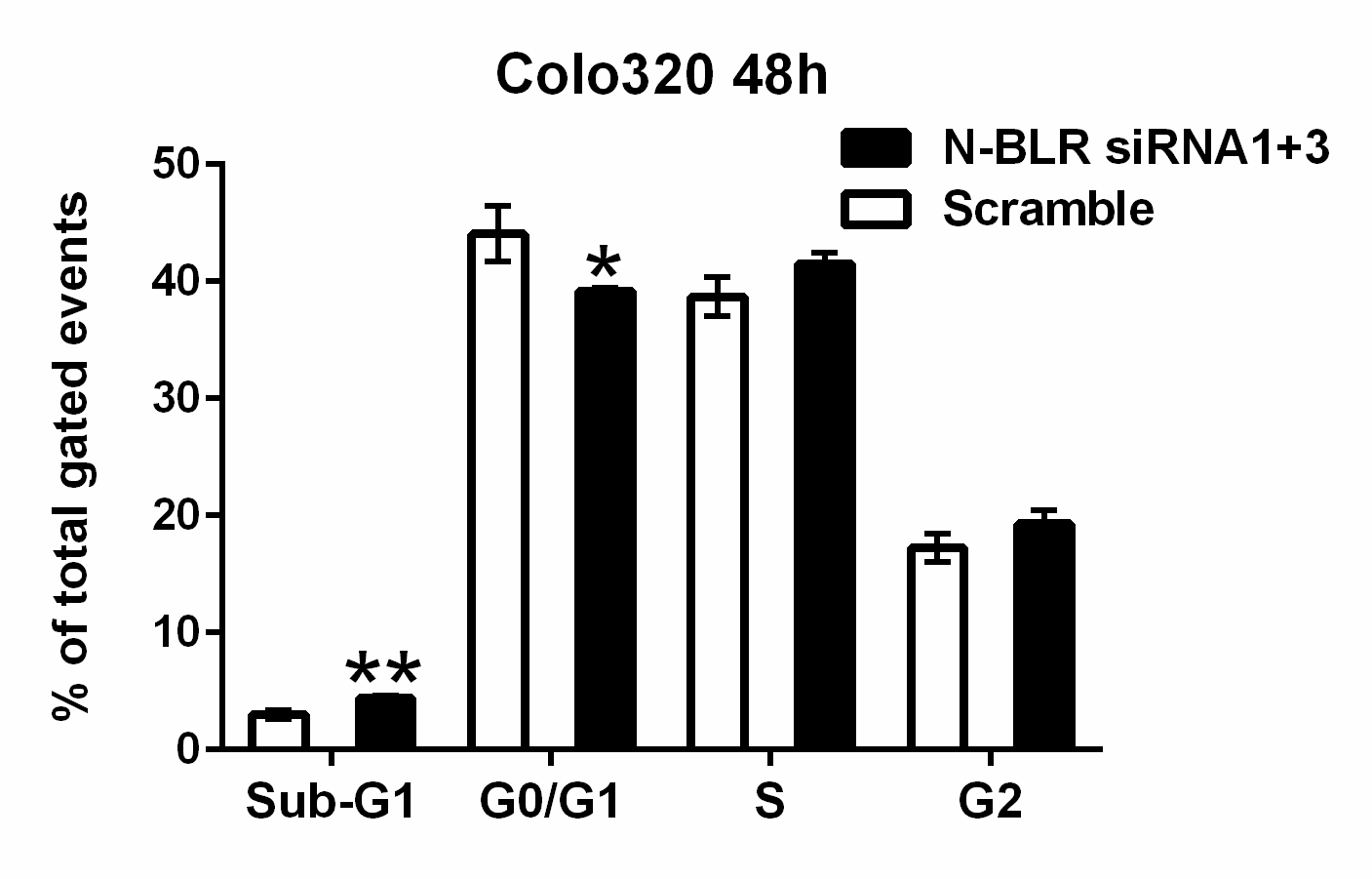

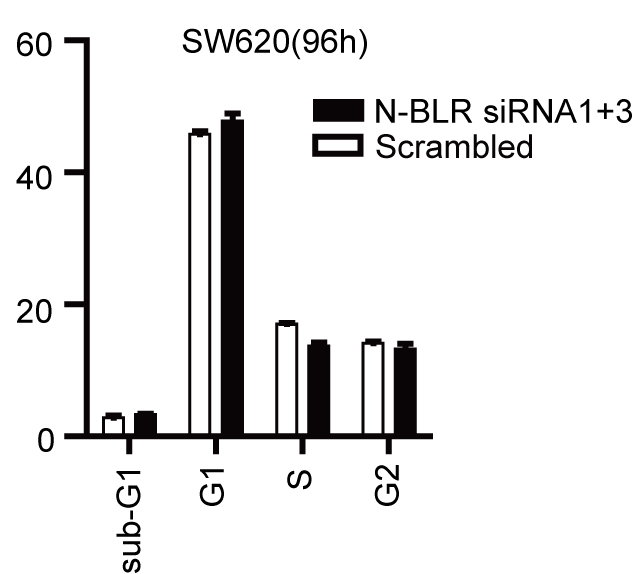

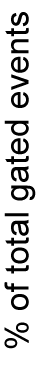

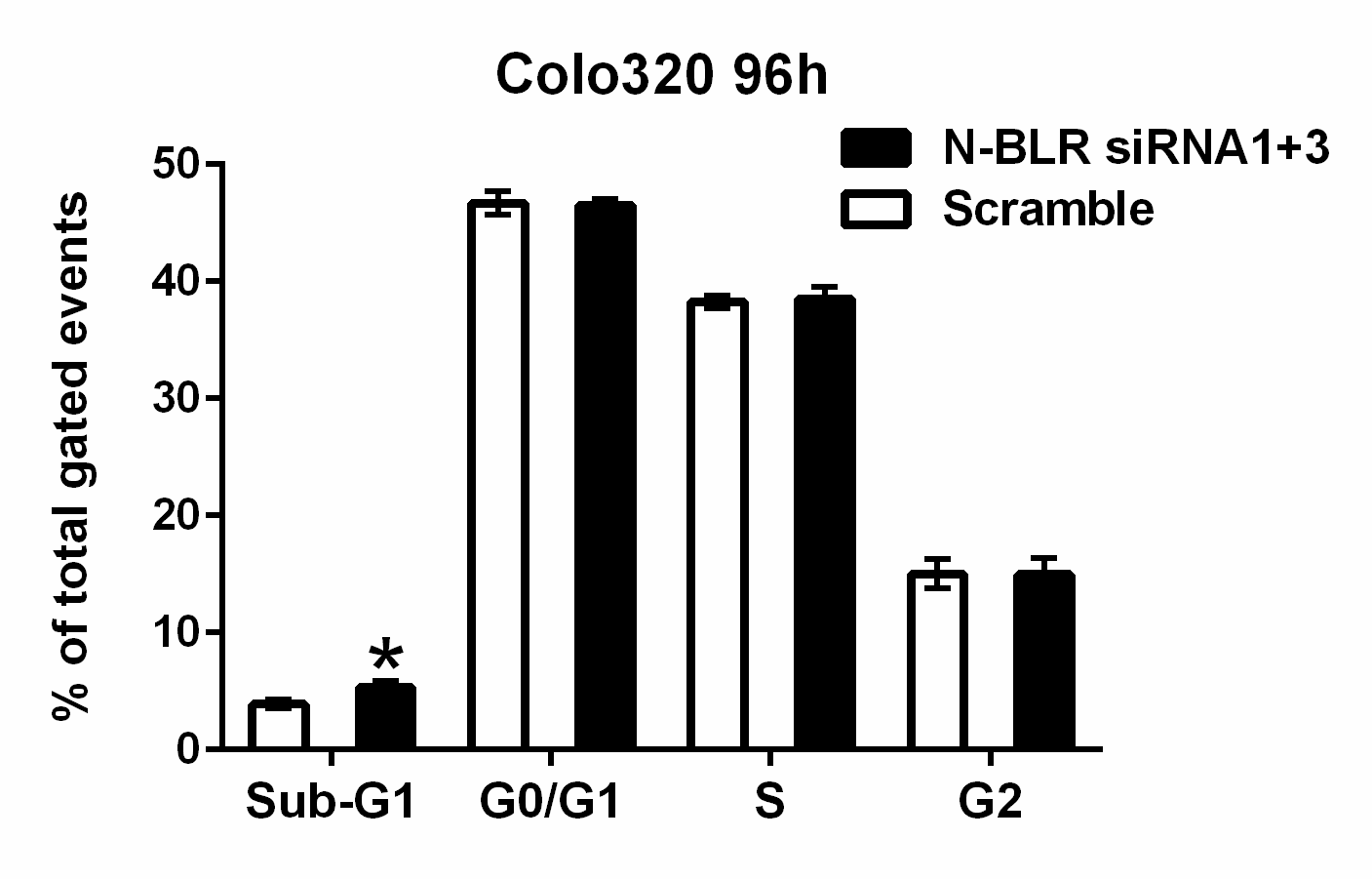


**Additional file 3: Fig. S7. N-BLR downregulation induces apoptosis in multiple cell models of colon cancer. (A)** Validation and extended expression analysis of N-BLR levels in colon cancer cell lines. **(B)** Assessing the effect of siRNA-mediated N-BLR downregulation in two additional cell lines, SW480 and RKO. We measured the levels of the downstream Caspases 3/7, observing a significant increase in their activity already upon 96 h and 120 h after siRNA transfection. **(C)** Conversely, stable overexpression of N-BLR WT in HCT116 and SW620 cells led to a reduction of Caspases-3/7 activity in both stable clones and, thus, apoptosis. Data are shown as mean ± SEM. (n=3; Student´s t-test; **p*<0.05; ***p*<0.01; ****p*<0.001).


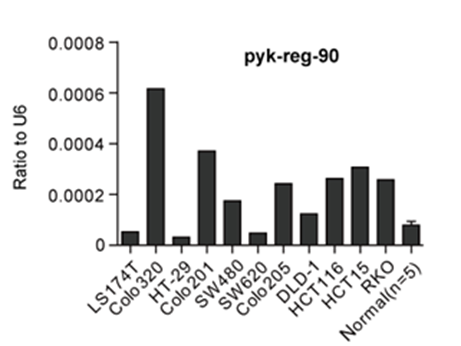


**pyk-reg-90 (N-BLR)**

**A**

**B**


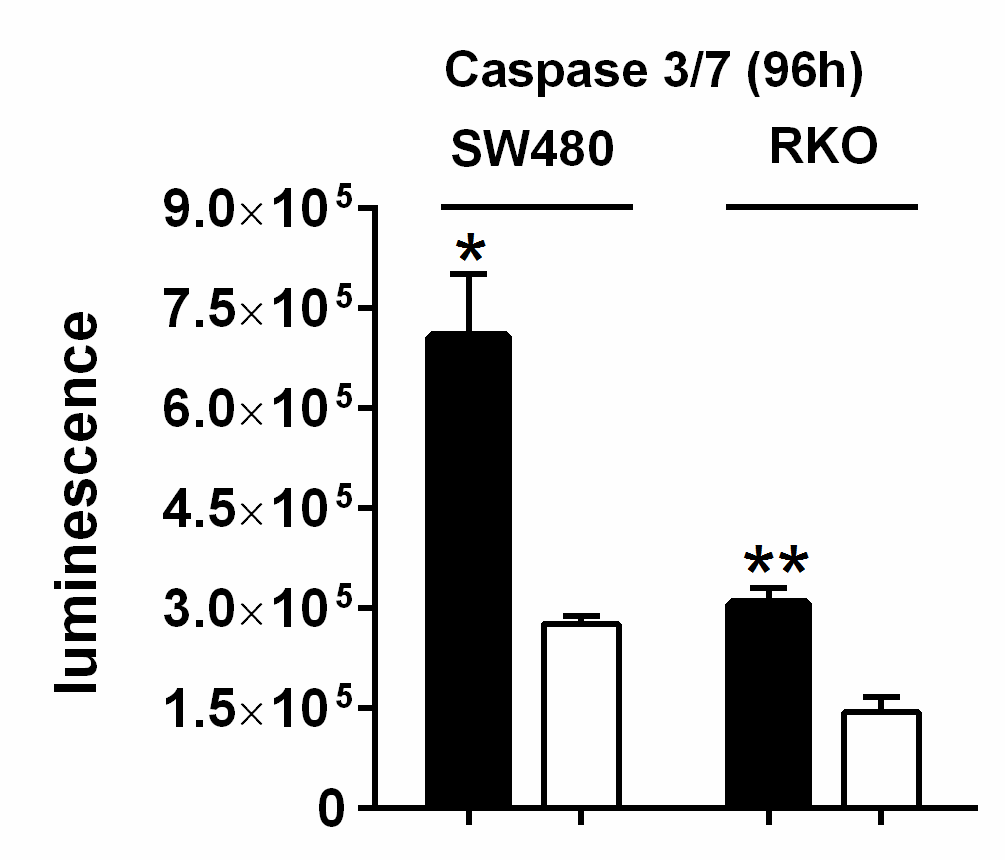

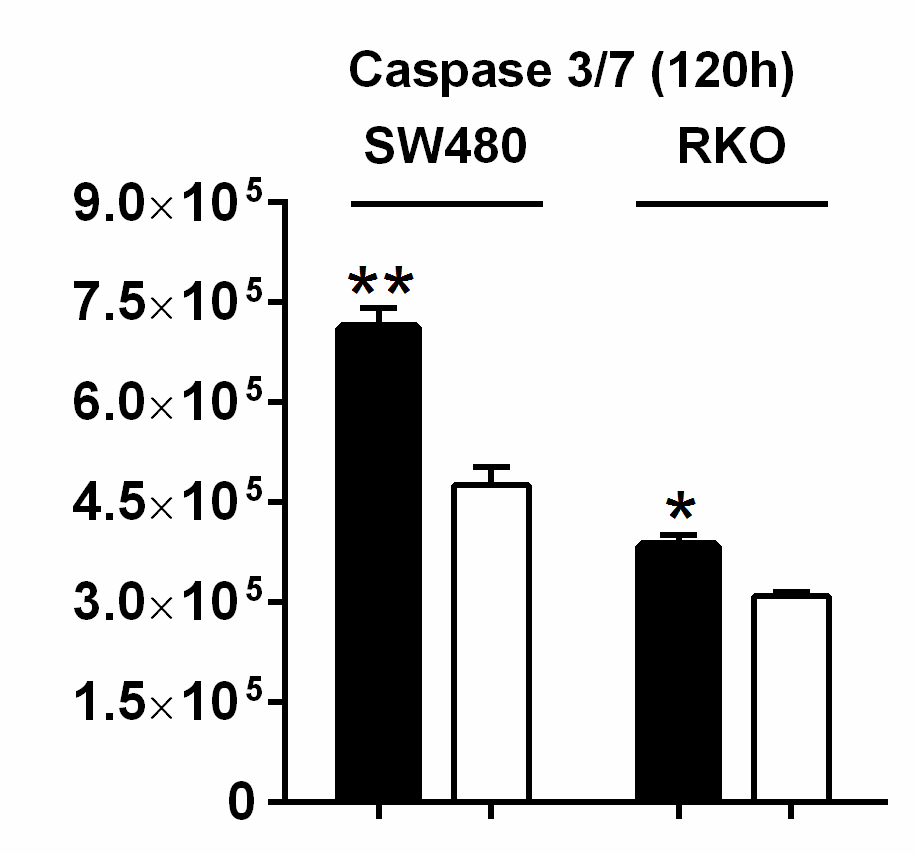


**N-BLR siRNA1+3**

**Scrambled**


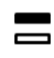


**Empty vector**

**N-BLR WT**


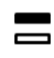

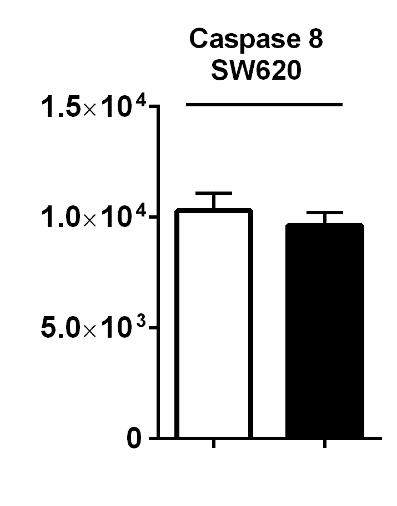

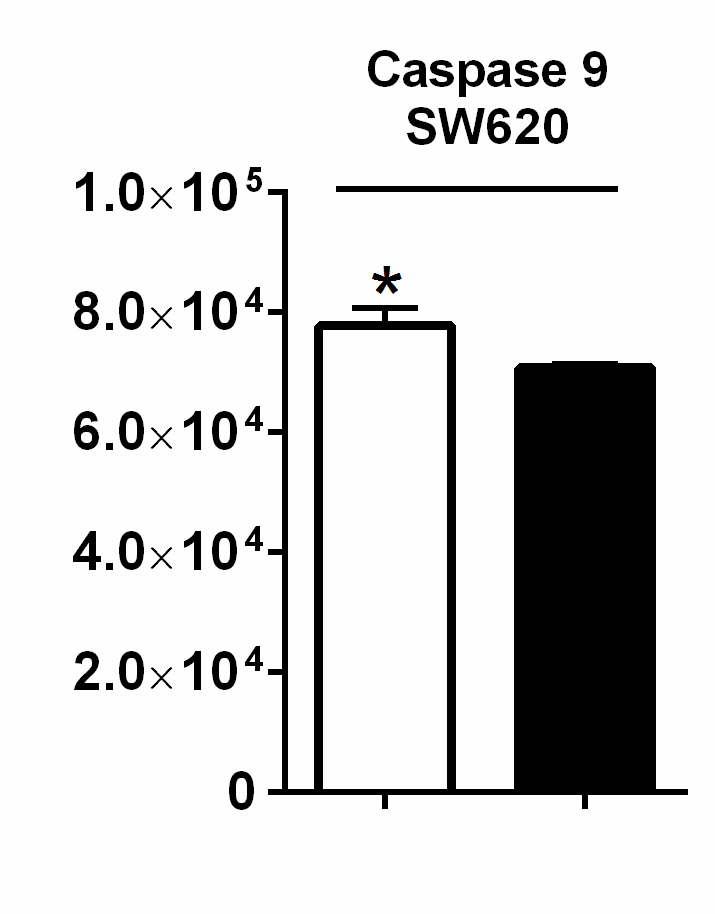

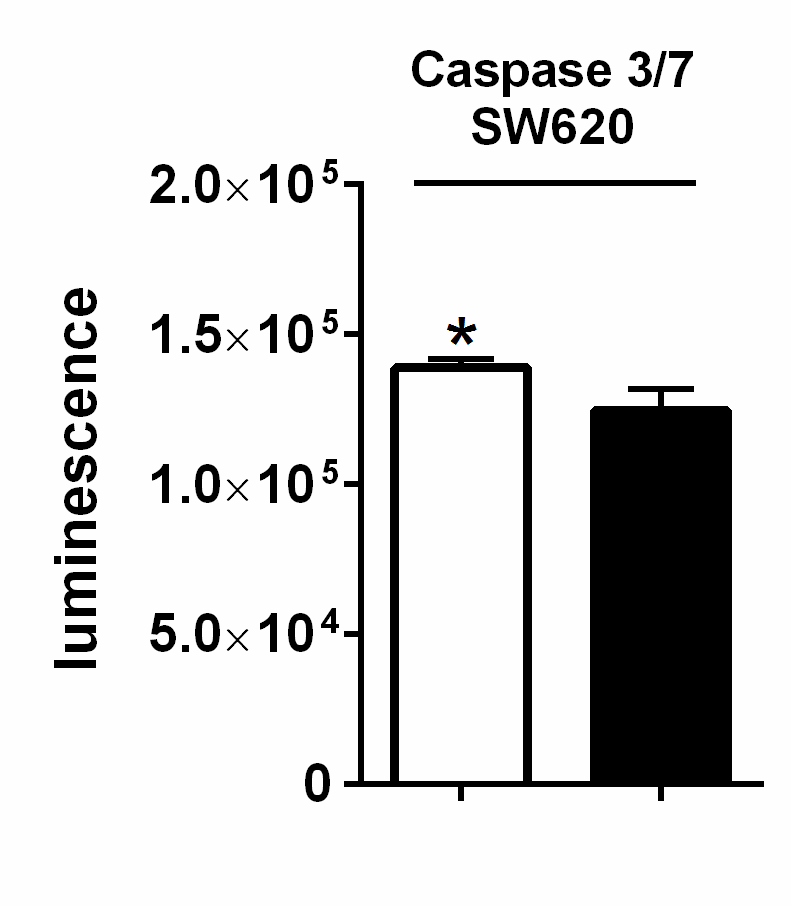

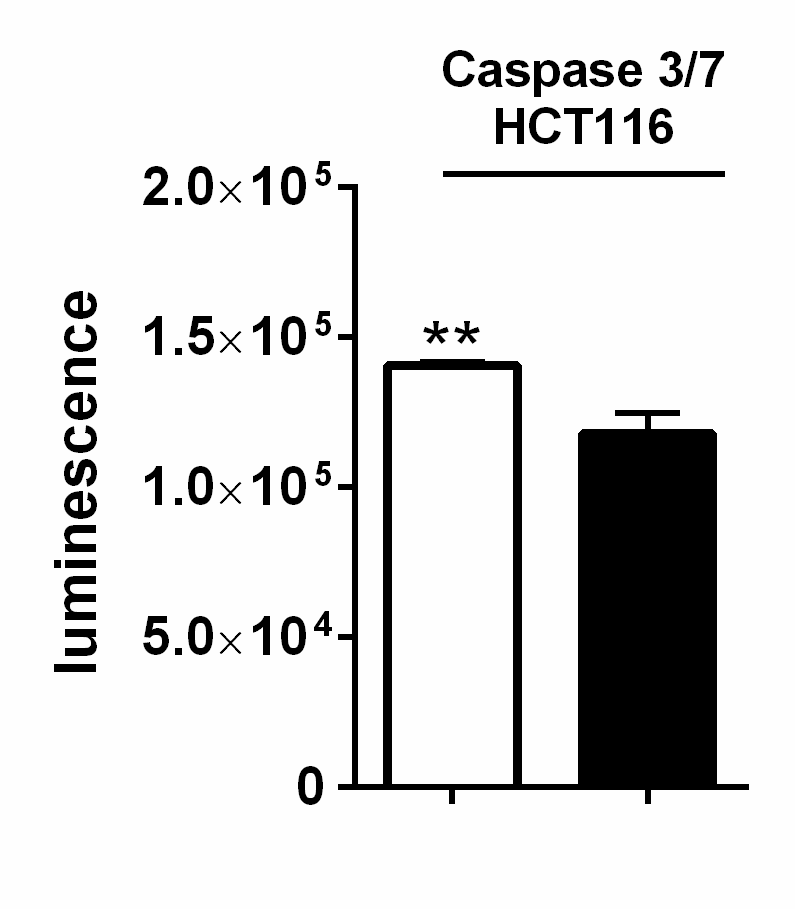

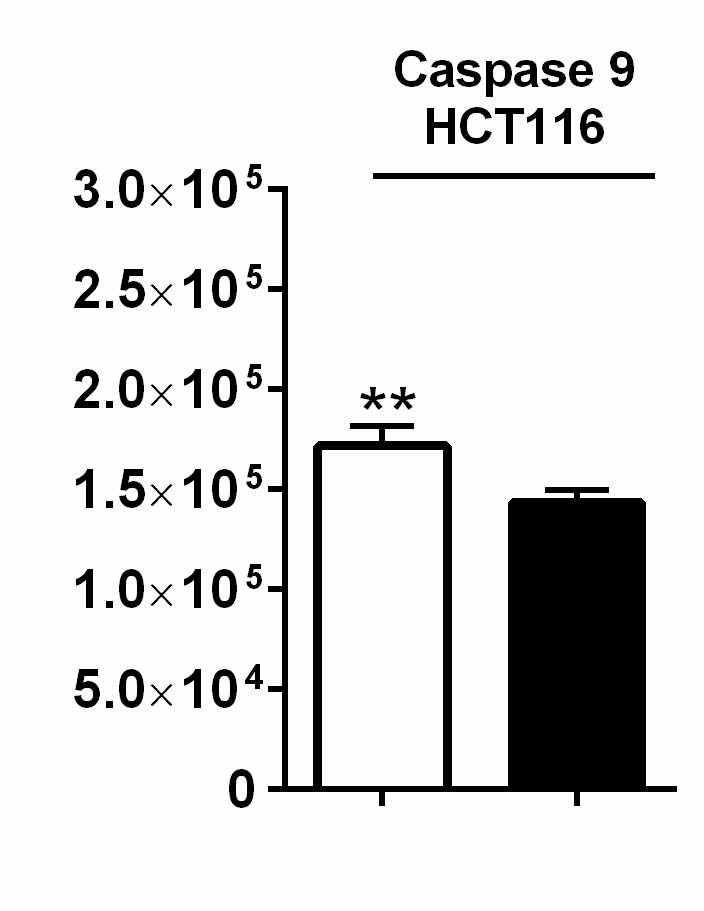

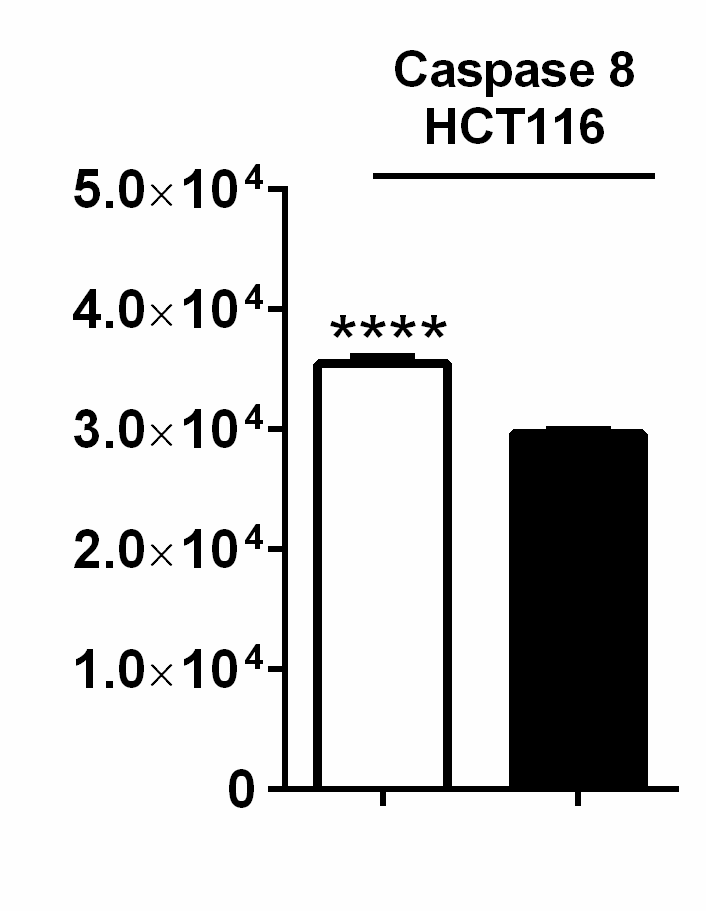


**C**

**Additional file 3: Fig. S8. Transient transfection of RKO cells with WT N-BLR regulates migration and invasion ability. (A)** RKO cells were transiently transfected with WT N-BLR vector and after 48 h cells were evaluated for N-BLR expression. **(B-C)** Transfected RKO were tested in a migration and invasion assay. Migration and invasion experiments were conducted in triplicate and results were normalized by proliferation rate of each cell type. Data are shown as mean ± stdev. (Student´s t-test; **p*<0.05; ***p*<0.01; ****p*<0.001; *****p*<0.0001). All experiments were performed in triplicate.

**A**

**B**

**C**


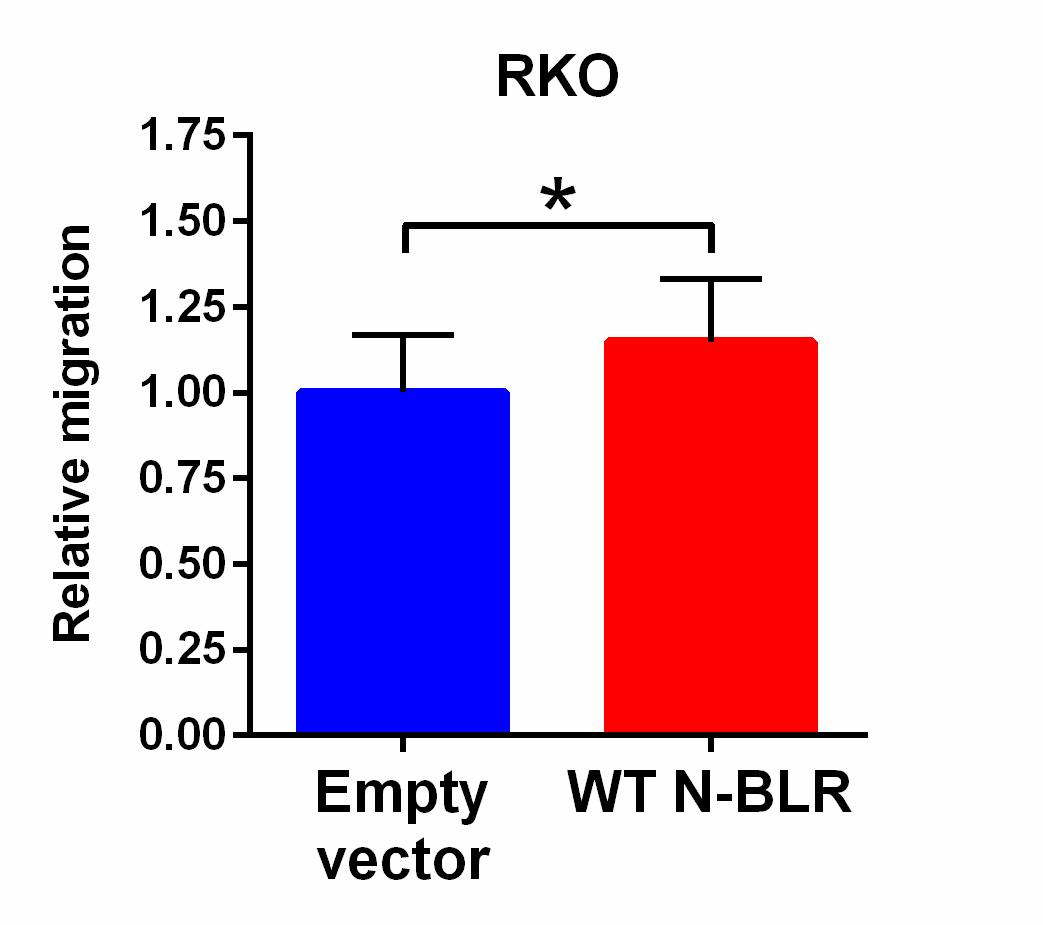

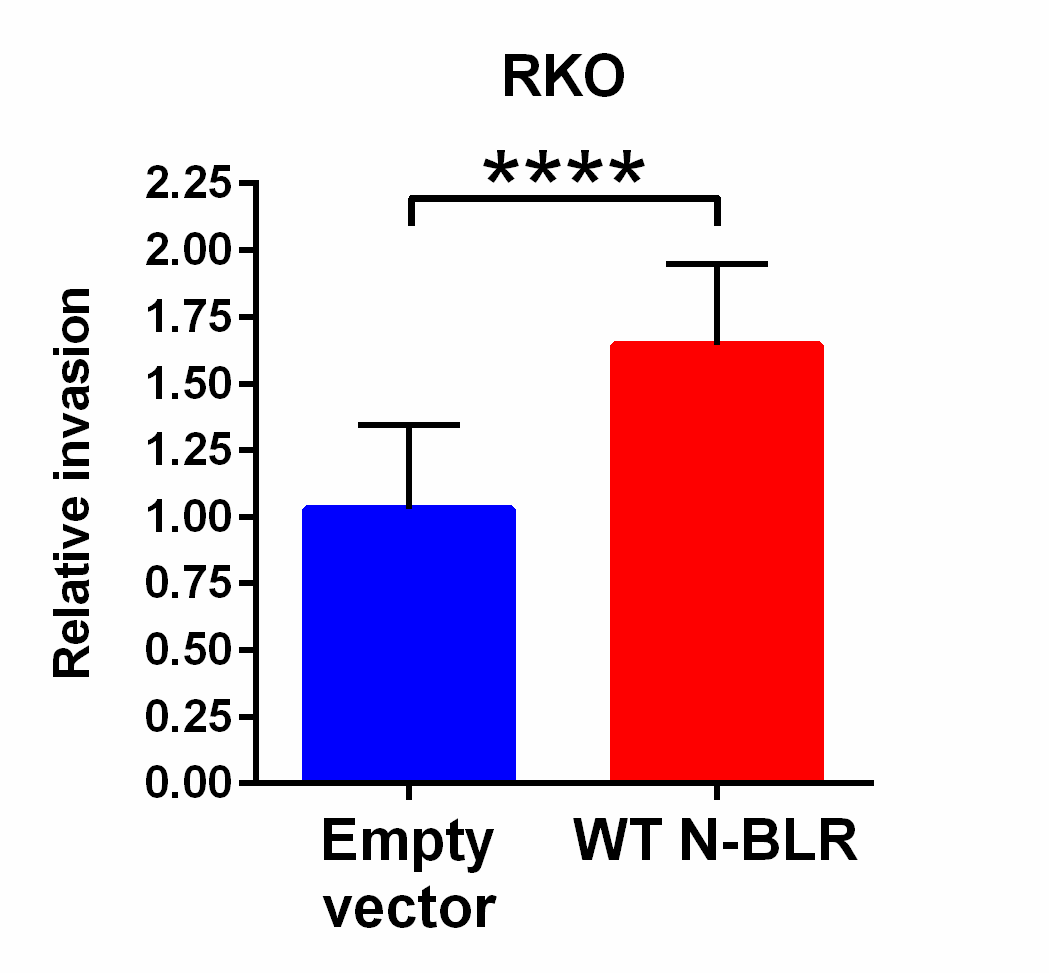

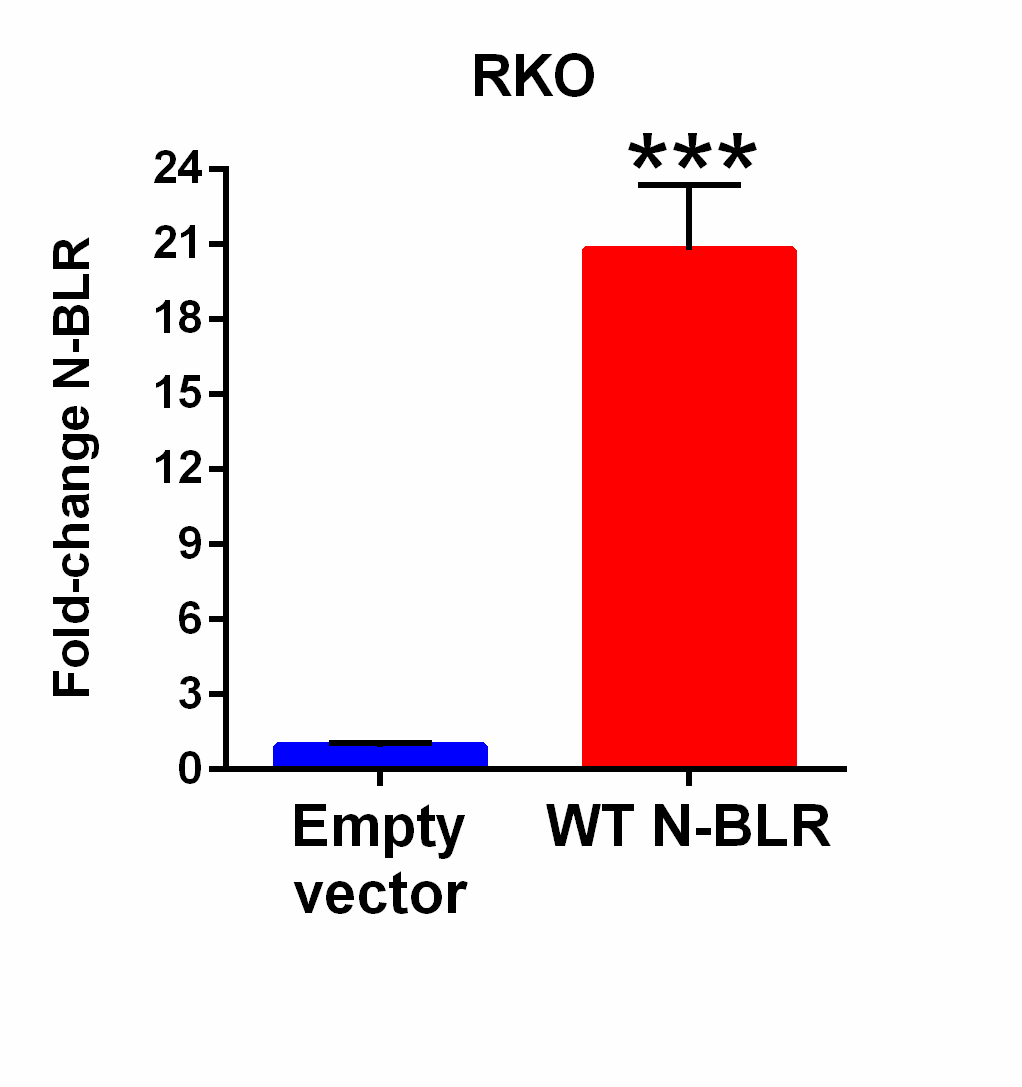


**Additional file 3: Fig. S9. Immunofluorescence for E-cadherin, Vimentin, and ZEB1.** **(A)** E-cadherin and Vimentin were identified by immunofluorescence with specific antibodies. Immunofluorescence signal of E-cadherin (Texas red, in red color) was markedly increased in both clones. The Vimentin signal was present in cells with Empty vector (red color) but not in clones #3-1 and #4-7. Clone control represents the HCT116 stable clone transfected with control shRNA. Blue color dots (DAPI) highlight the nuclei. This represents an independent experiment of the one in Figure. 3F. **(B** and **C)** ZEB1 expression levels in HCT116 stable shRNA N-BLR clones (#3-1 and #4-7) and shRNA control and in HCT116 overexpressing WT N-BLR and Pyk90-DEL N-BLR (These images are the single green and blue channels from Figure 3F and Figure 5F, respectively).

**
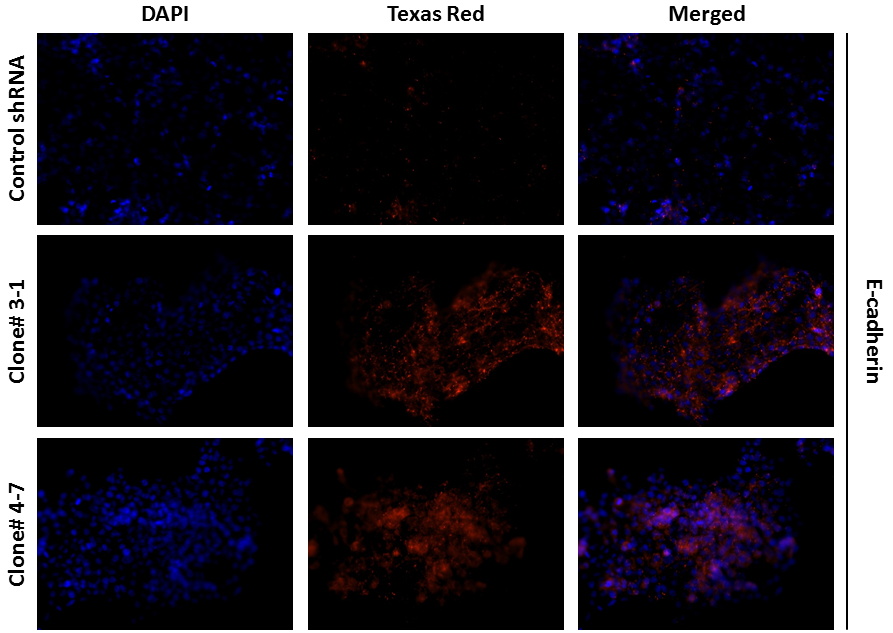
**

**A**

**
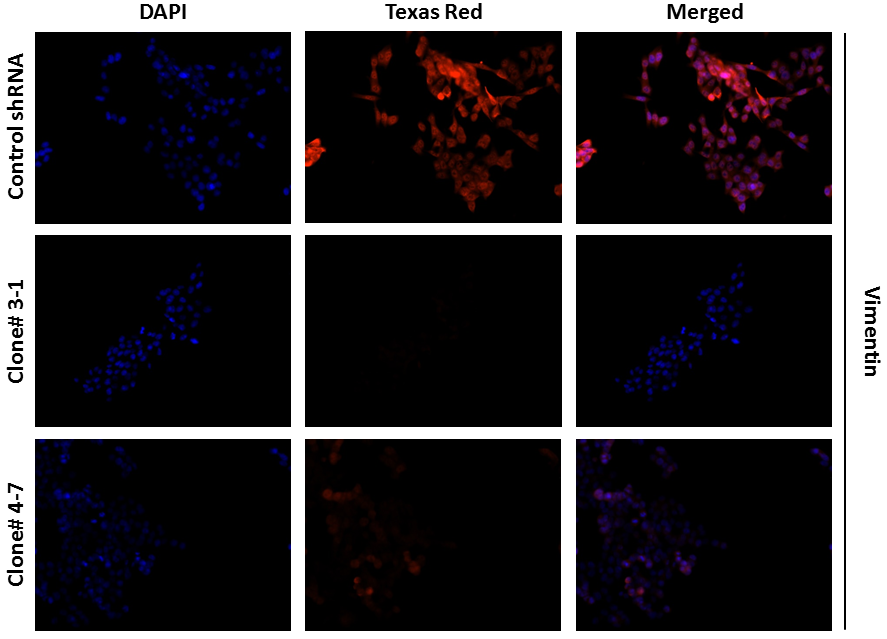
**

**Additional file 3: Fig. S9 (Continued).**

**B**


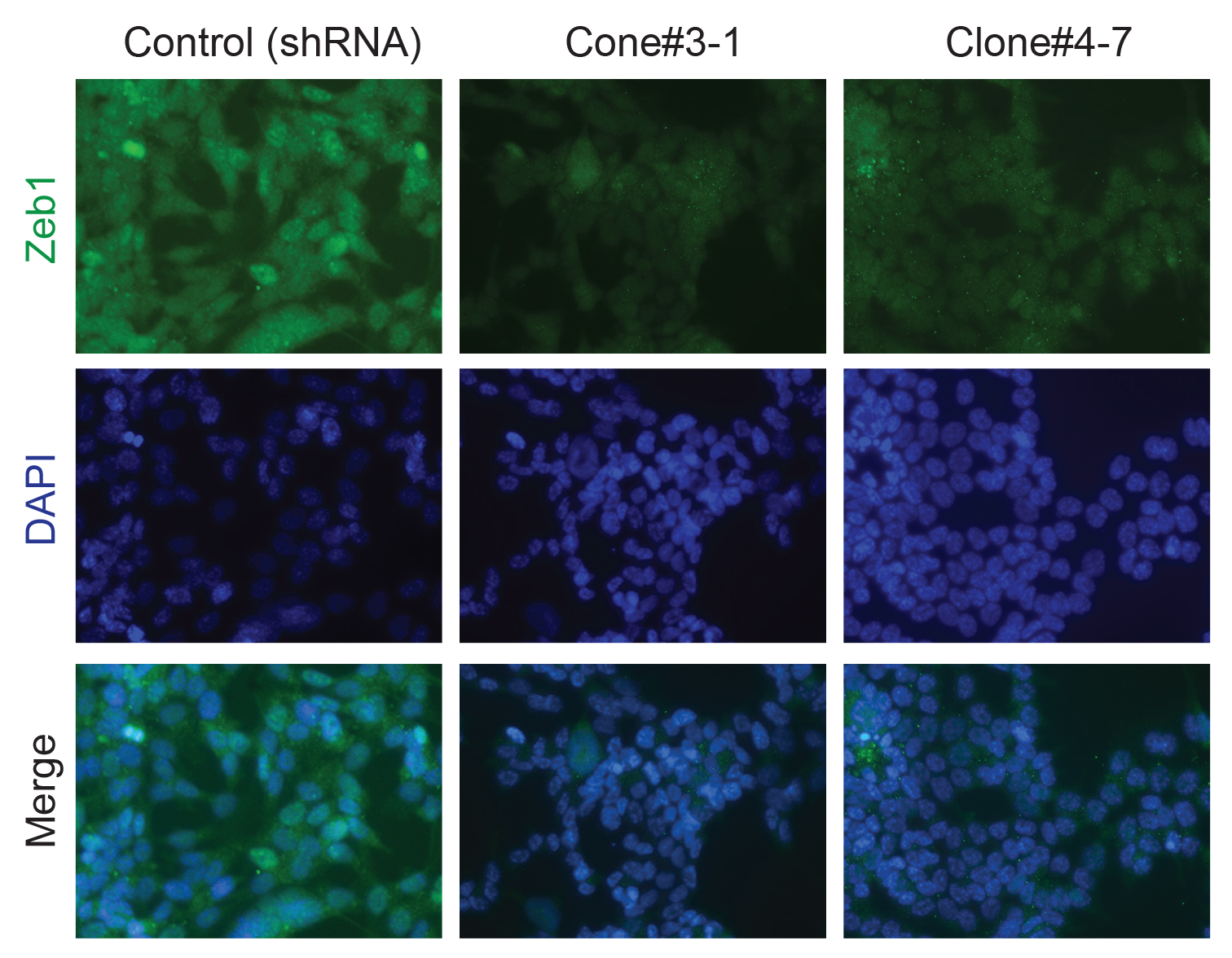


Control (shRNA) (shRNA)

Clone#3-1

Clone#4-7


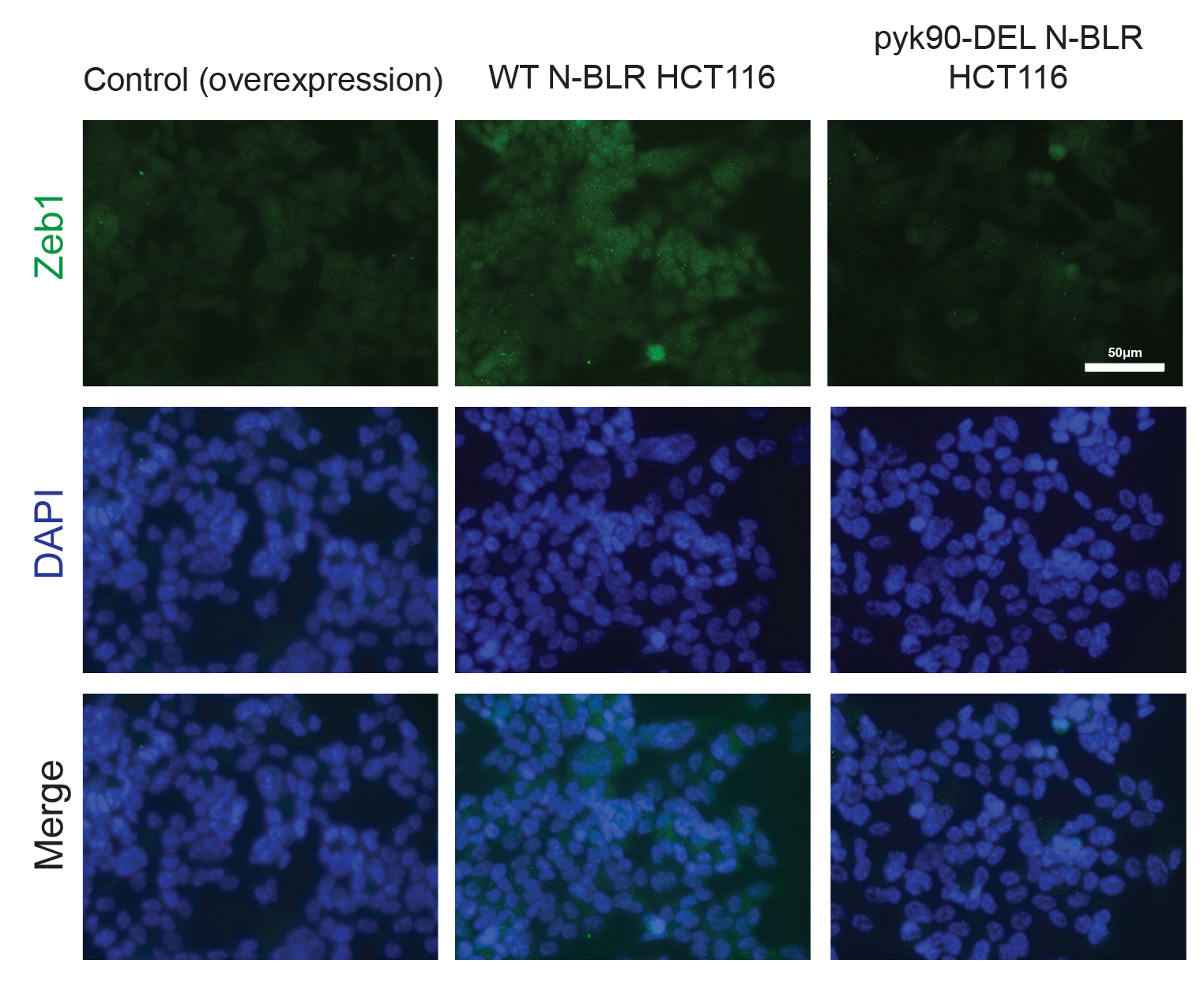
 **C**

**Additional file 3: Fig. S10. Evidence of direct targeting of N-BLR by miR-200c/miR-141.** **(A)** Schematic of the *in silico* prediction for miR-200c-3p and miR-141 within the N-BLR sequence by the RNA22 algorithm. **(B)** miRNA expression in HCT116 shRNA stable clones. MiR-141 and miR-200c have increased levels in both clones (#3-1 and #4-7). **(C)** RKO cells were transfected with 100 nM siRNA pool for 48 h to knockdown N-BLR. Then, the levels of N-BLR, miR-200c-3p, and miR-141-3p were evaluated by qRT-PCR. **(D)** RKO cells were transiently transfected with vectors containing wild type N-BLR and empty vector, as control. The expression levels of N-BLR, miR-141-3p, and miR-200c-3p were measured by qRT-PCR after 48 h of transfection. Data are shown as mean ± stdev. (n=3; Student´s t-test; **p*<0.05; ***p*<0.01; ****p*<0.001; *****p*<0.0001).


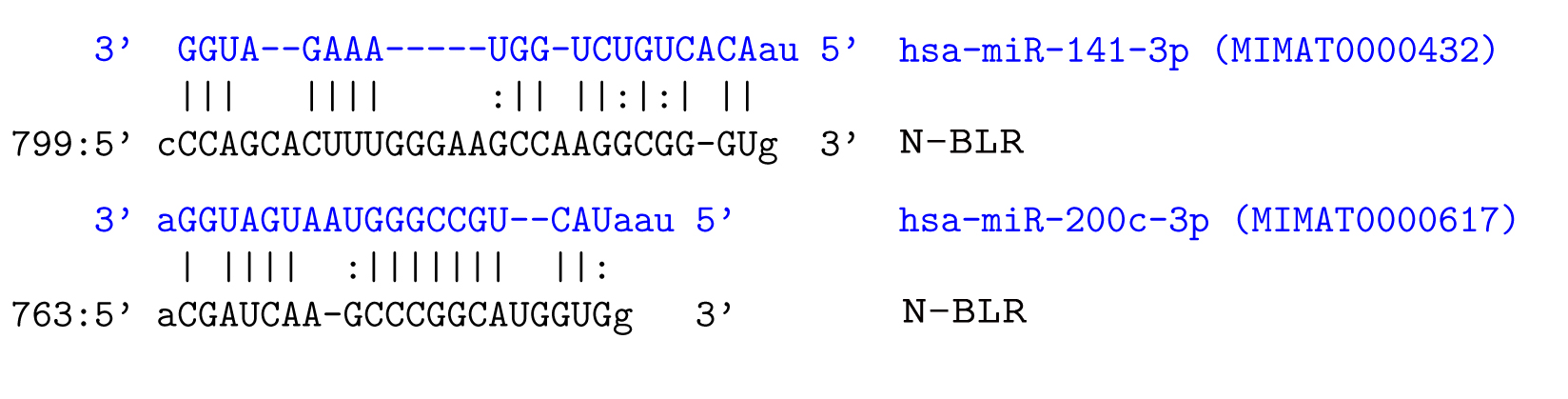


**A**

**C**


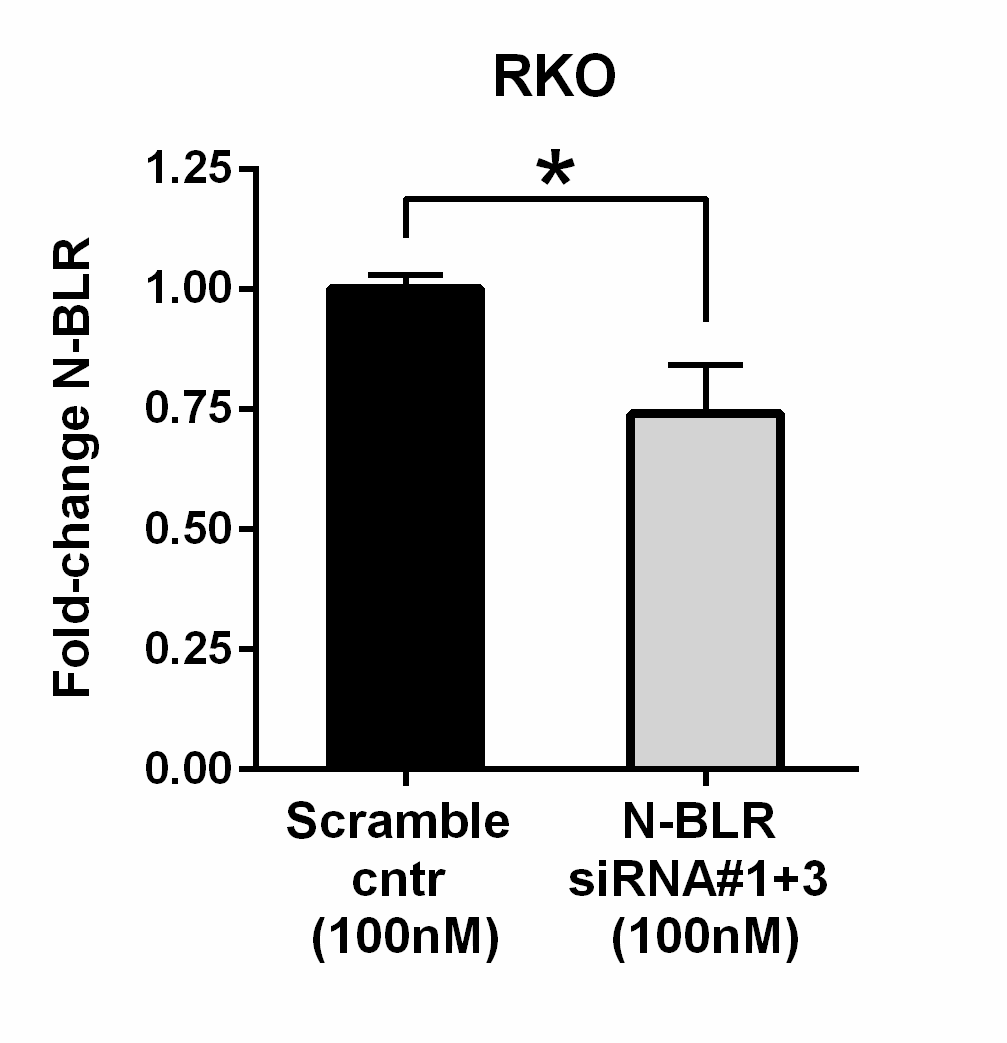

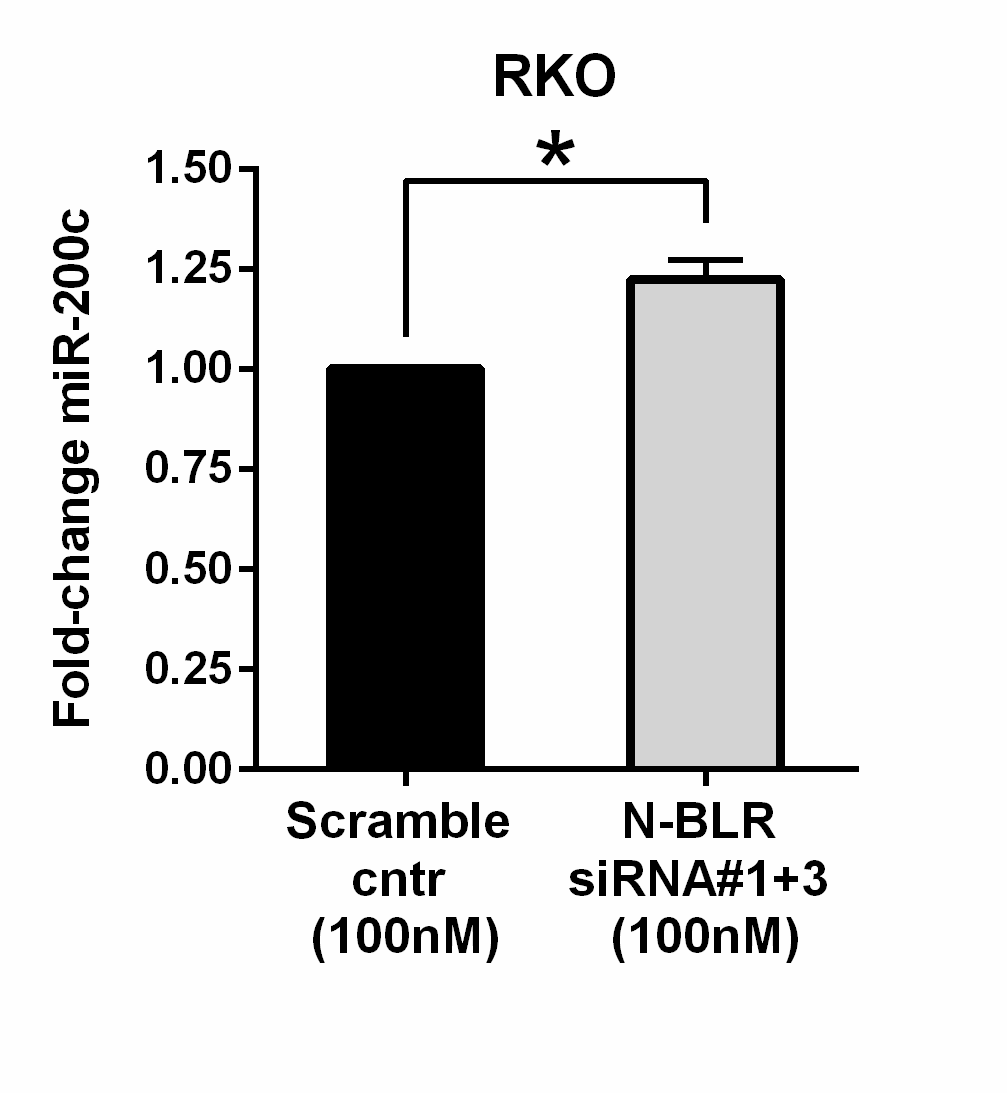

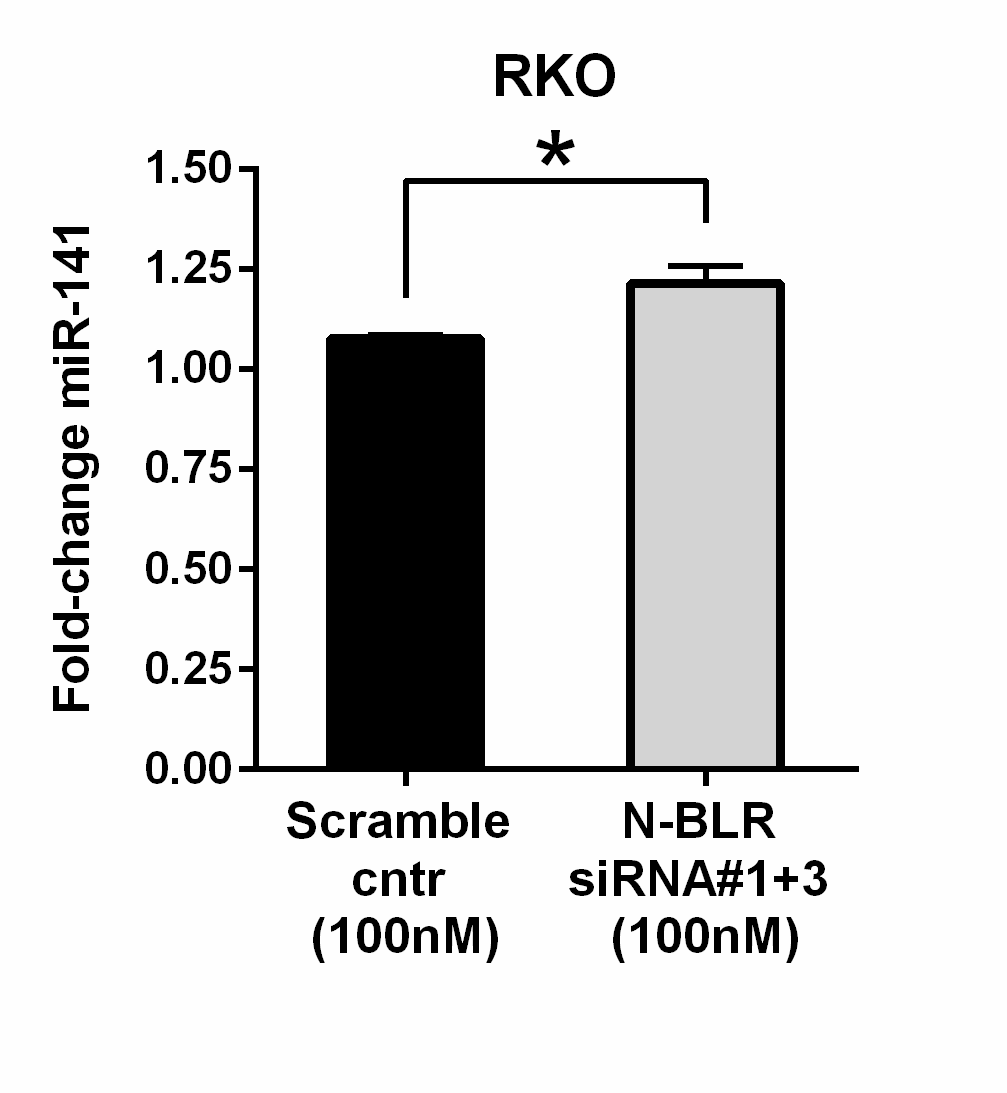

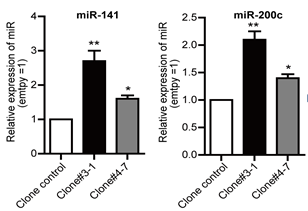


**HCT116**

**HCT116**


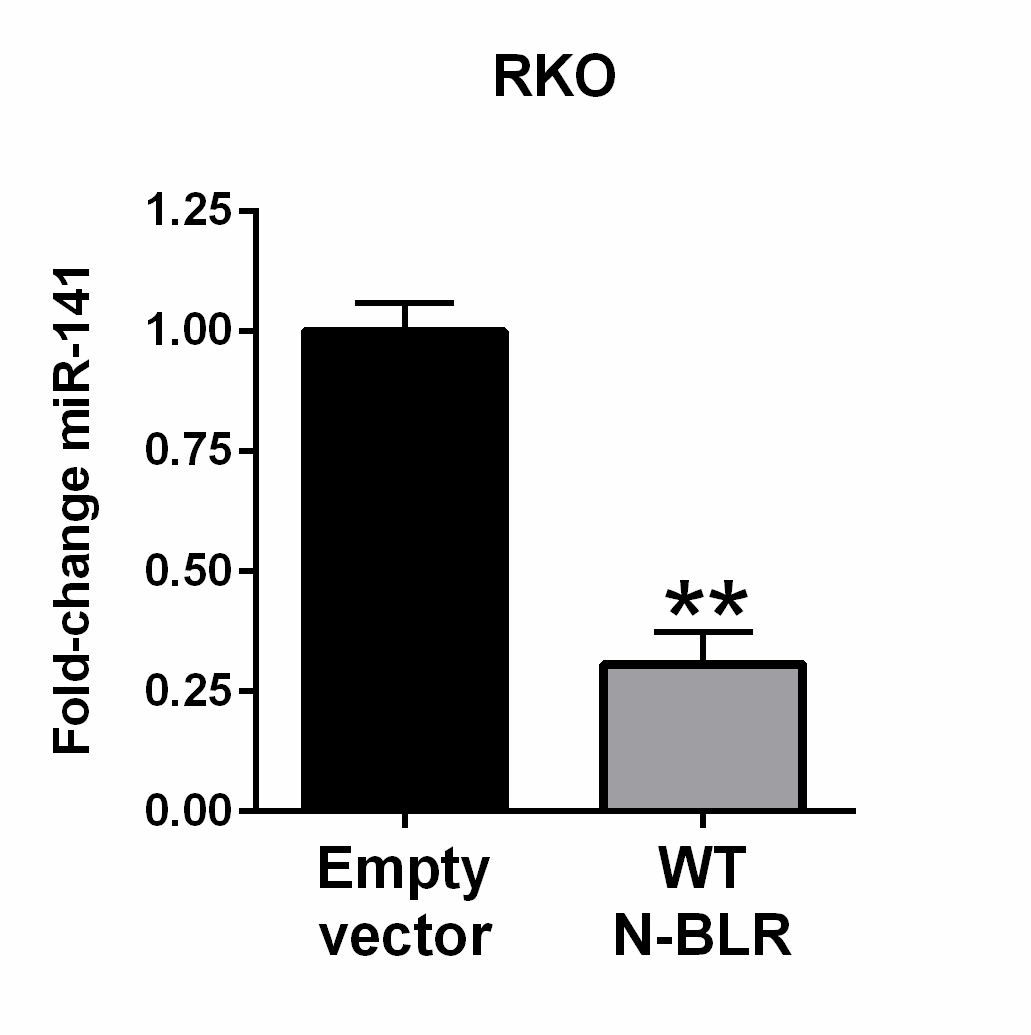

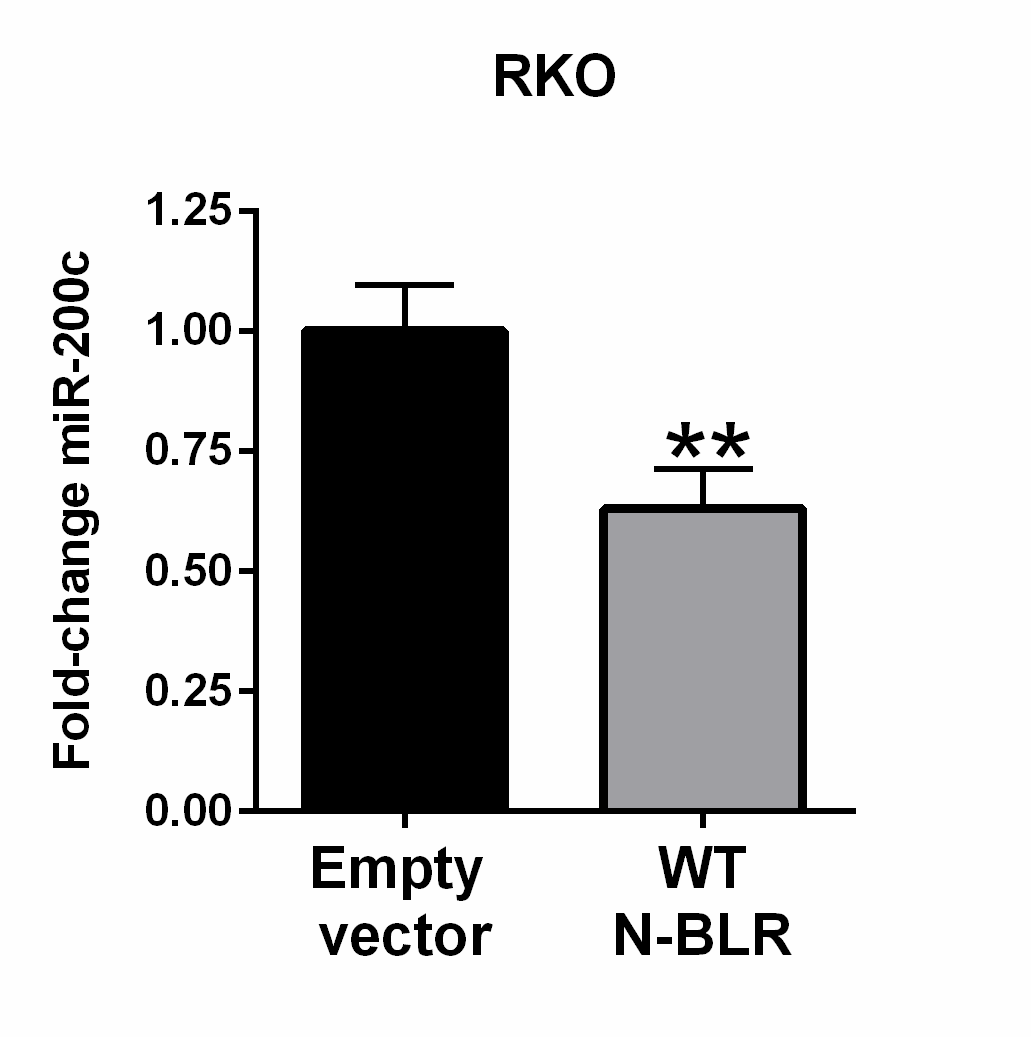

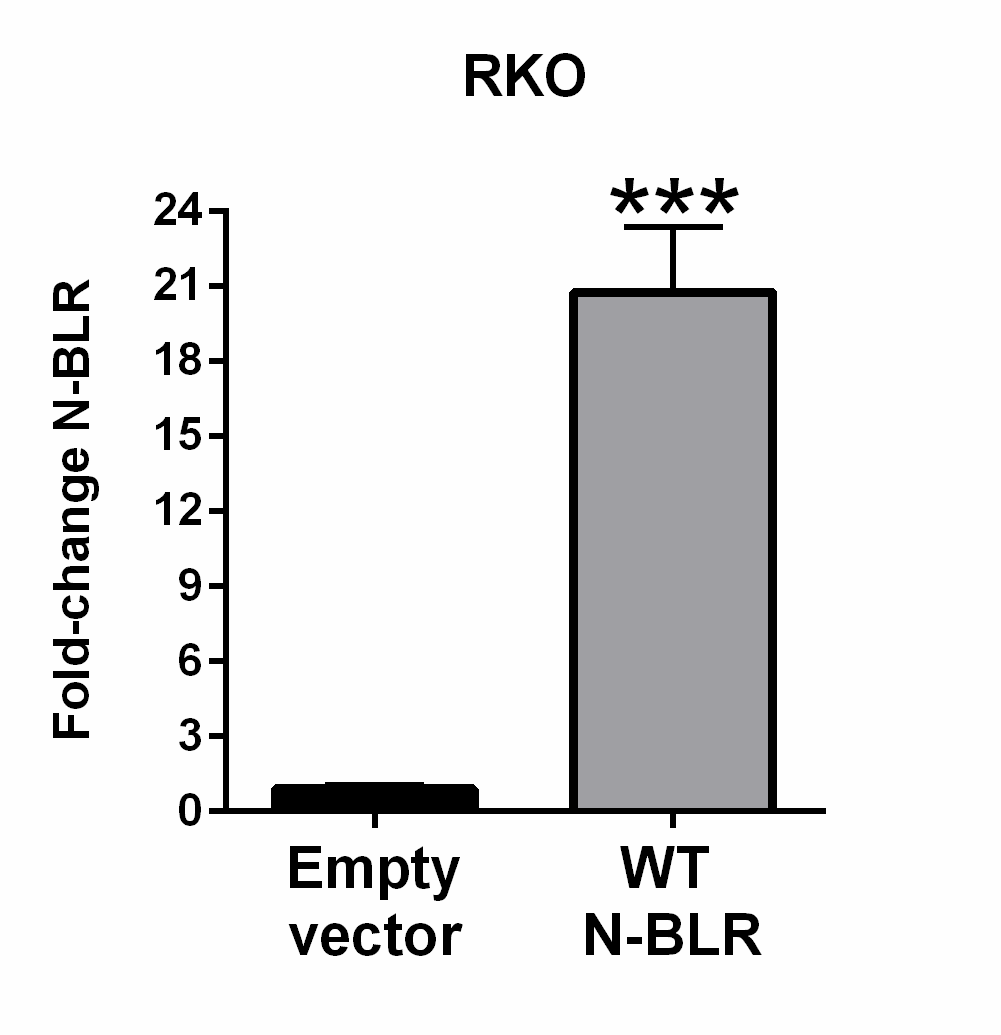


**B**

**D**

**Additional file 3: Fig. S11. The levels of N-BLR in RKO cells at 48 hours after miR-141-3p or miR-200c-3p transfection.** RKO cells were transfected with 10 nM of miR-141-3p, miR-200c-3p mimic, and mimic control (Cntr). Data were generated in three independent wells for each condition. Data are shown as mean ± stdev. (n=3; Student´s t-test; **p*<0.05; ***p*<0.01; ****p*<0.001; *****p*<0.0001).


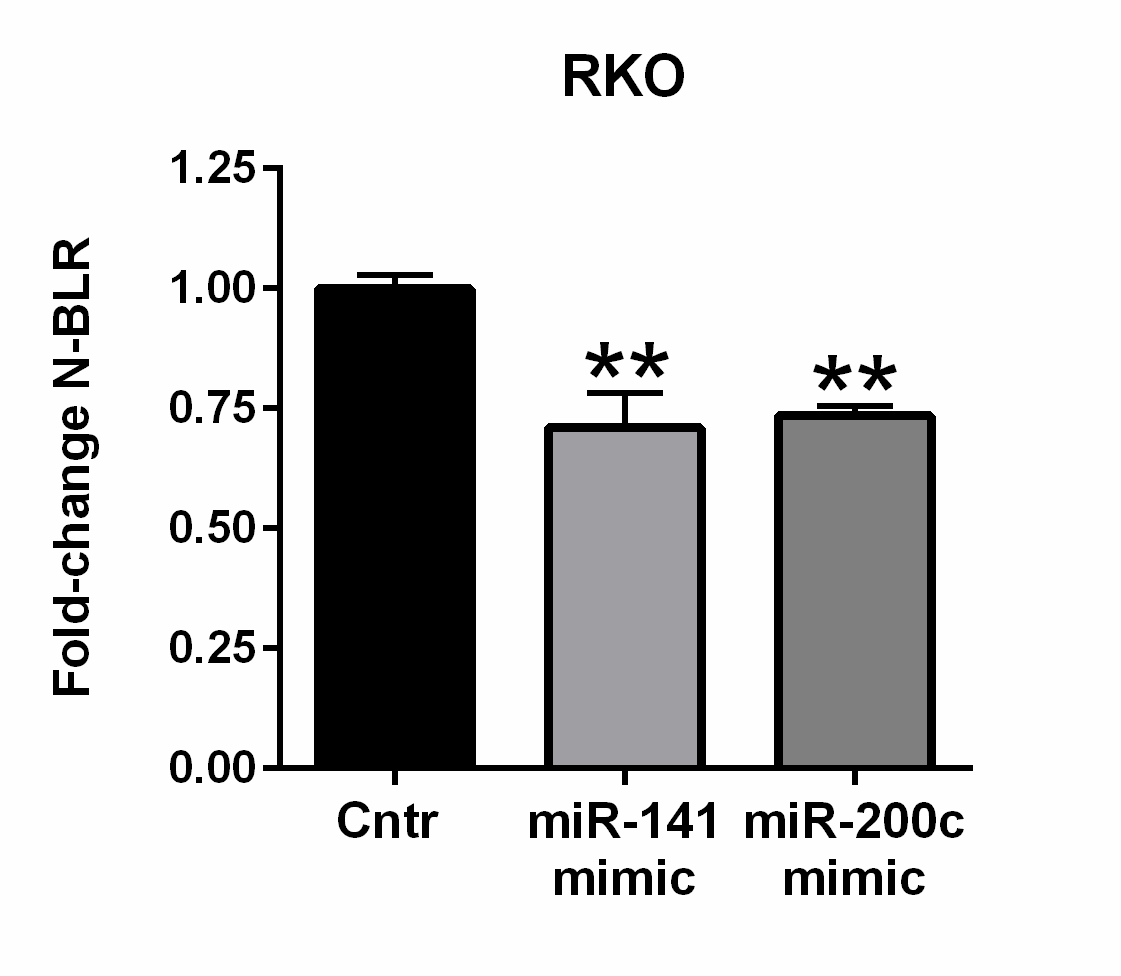


**Additional file 3: Fig. S12. Abundance of miR-141 and miR-200c by qRT-PCR measurements in CRC and normal colon samples. (A)** The abundance of miR-141 was lower in CRC samples with respect to normal controls as measured by qRT-PCR (Left). The TCGA datasets show the correlation between high expression of miR-141 and good 5-year survival. **(B)** Similarly to miR-141, miR-200c abundance was lower in colon cancer in the TCGA patient datasets (n=235) when compared to normal (n=8). The TCGA datasets show the correlation between high expression of miR-200c and good 5-year survival.


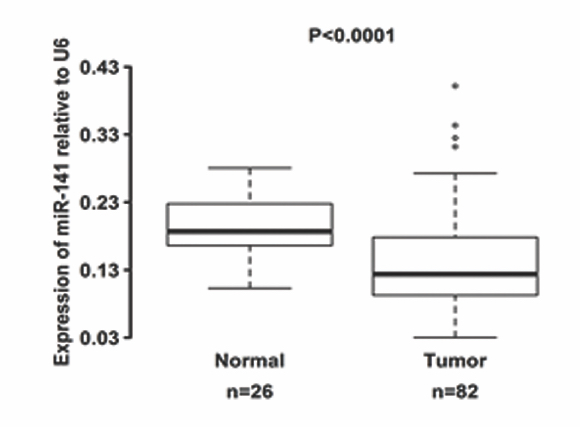
**A**


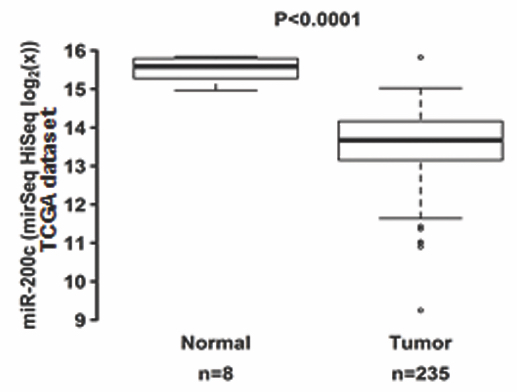
**B**

**Additional file 3: Fig. S13. The regulation of miR-200c-3p levels by N-BLR modulates the response to 5-FU chemotherapy. (A)** Colo320 cells were transiently transfected with miR-200c-3p at a concentration of 10 nM for 96 h and then treated with increasing concentrations of 5-FU for an additional 72h. The ectopic expression of miR-200c-3p was associated with decreased XIAP levels, both mRNA (left) and protein (center), thereby resulting in increased sensitivity to 5-FU-induced apoptosis at different concentrations (right). **(B)** HCT116 and RKO WT N-BLR overexpressing stable clones were treated with 10 μg/ml of 5-FU for 72h (left and right). **(C)** RKO cells were transiently transfected with WT-N-BLR vector and after 48 h levels of miR-200c-3p and XIAP were evaluated. Then, RKO cells were treated with 10 μg/ml of 5-FU for 72h (right). Data are shown as mean ± stdev. (n=3; Student´s t-test; **p*<0.05; ***p*<0.01; ****p*<0.001; *****p*<0.0001). The experiments were performed in triplicate.

**
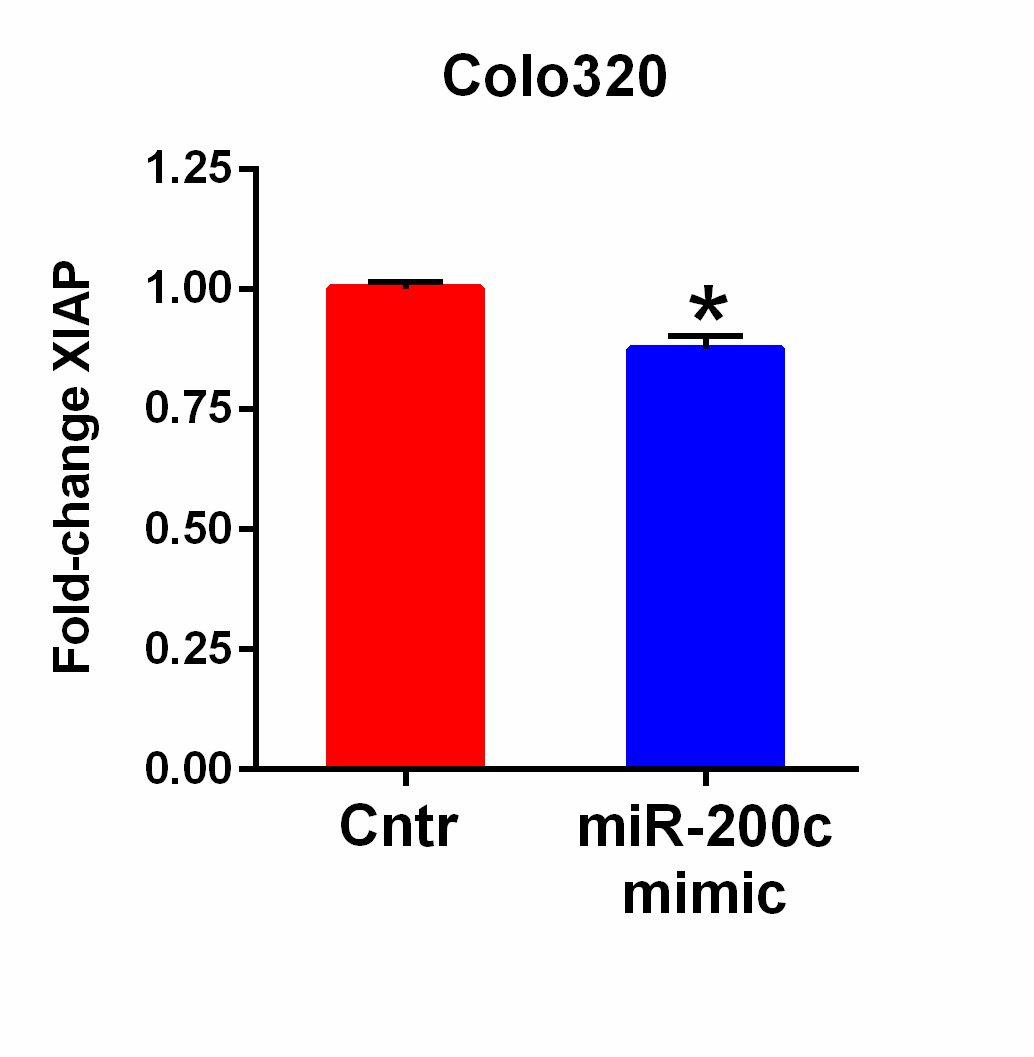

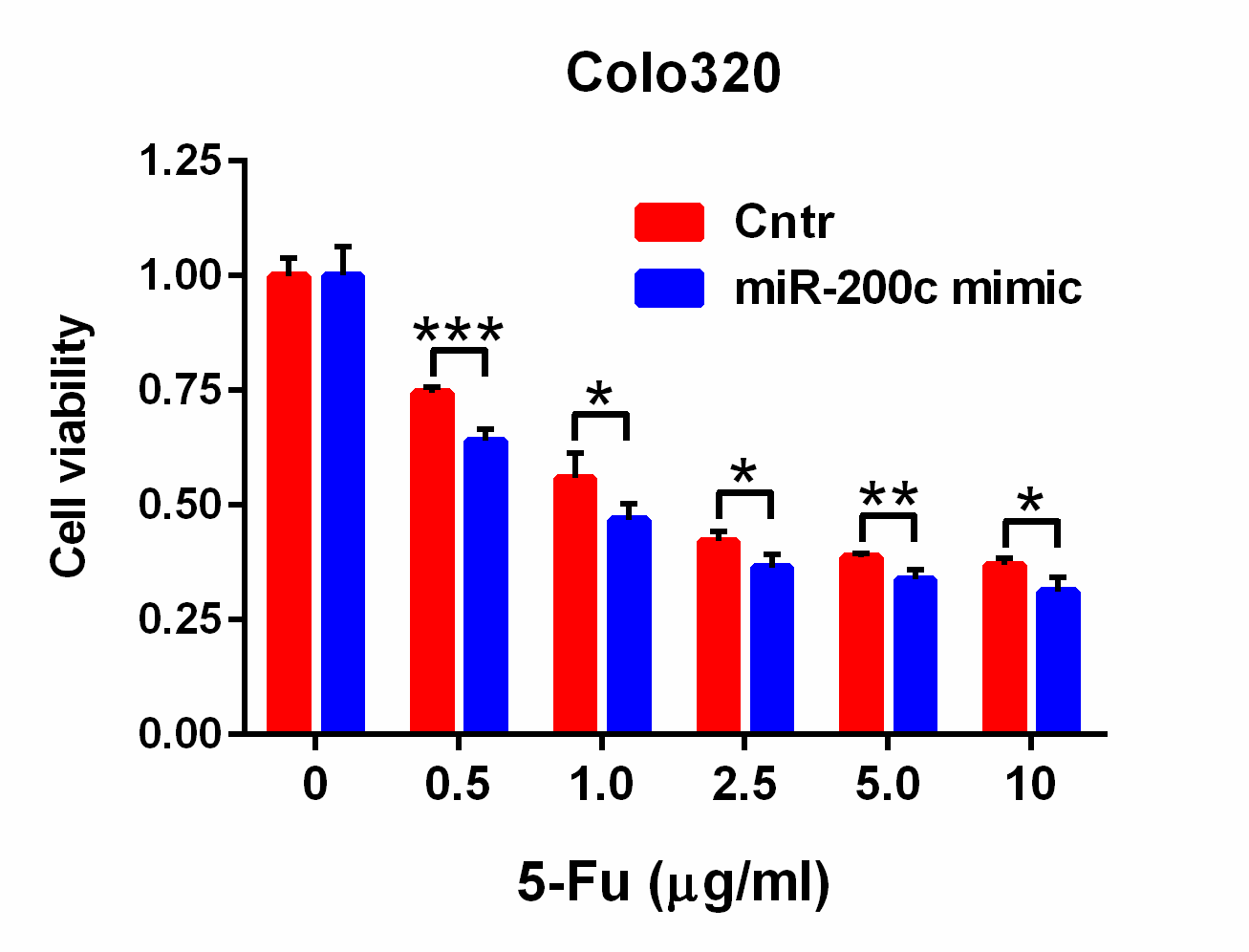

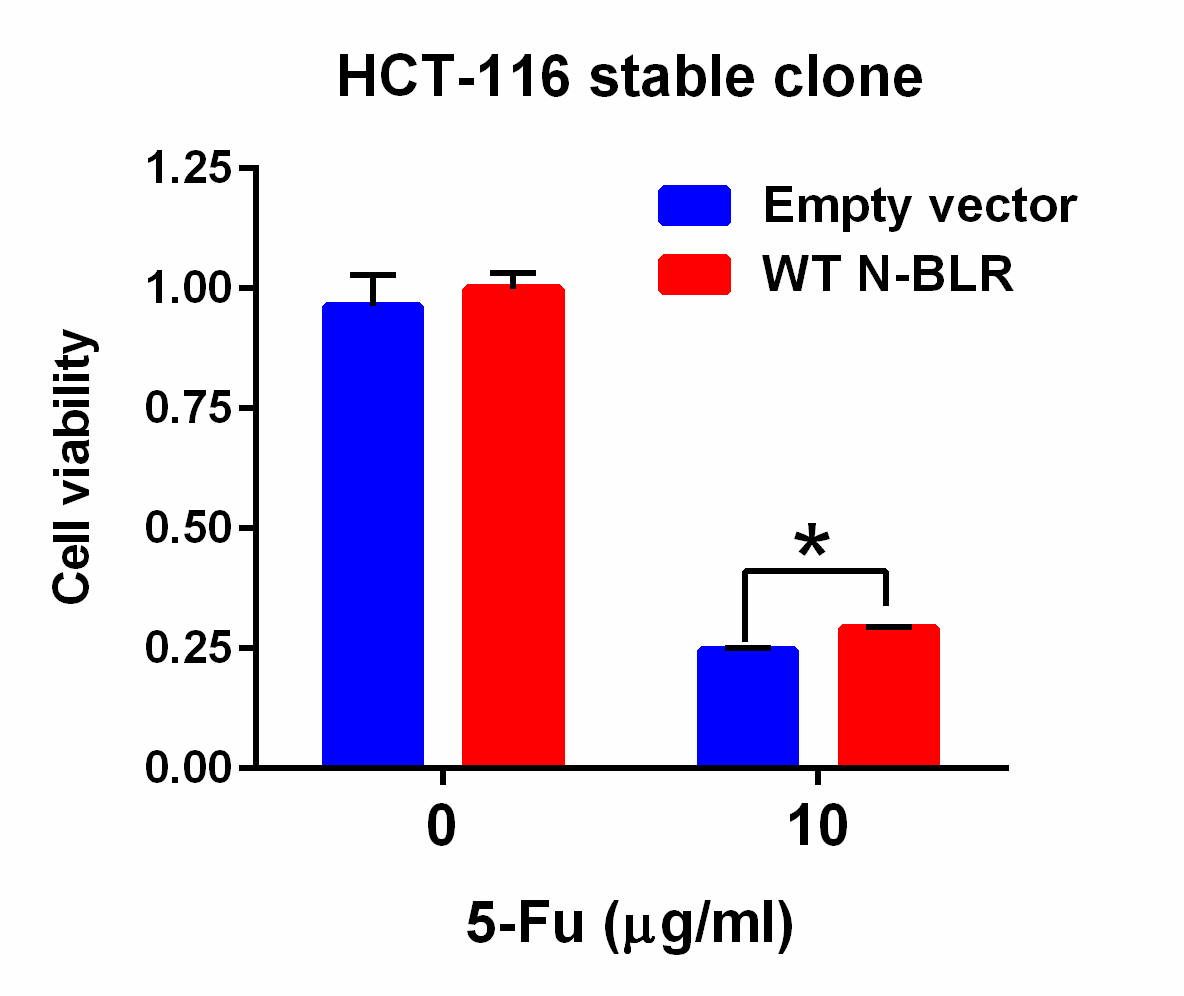

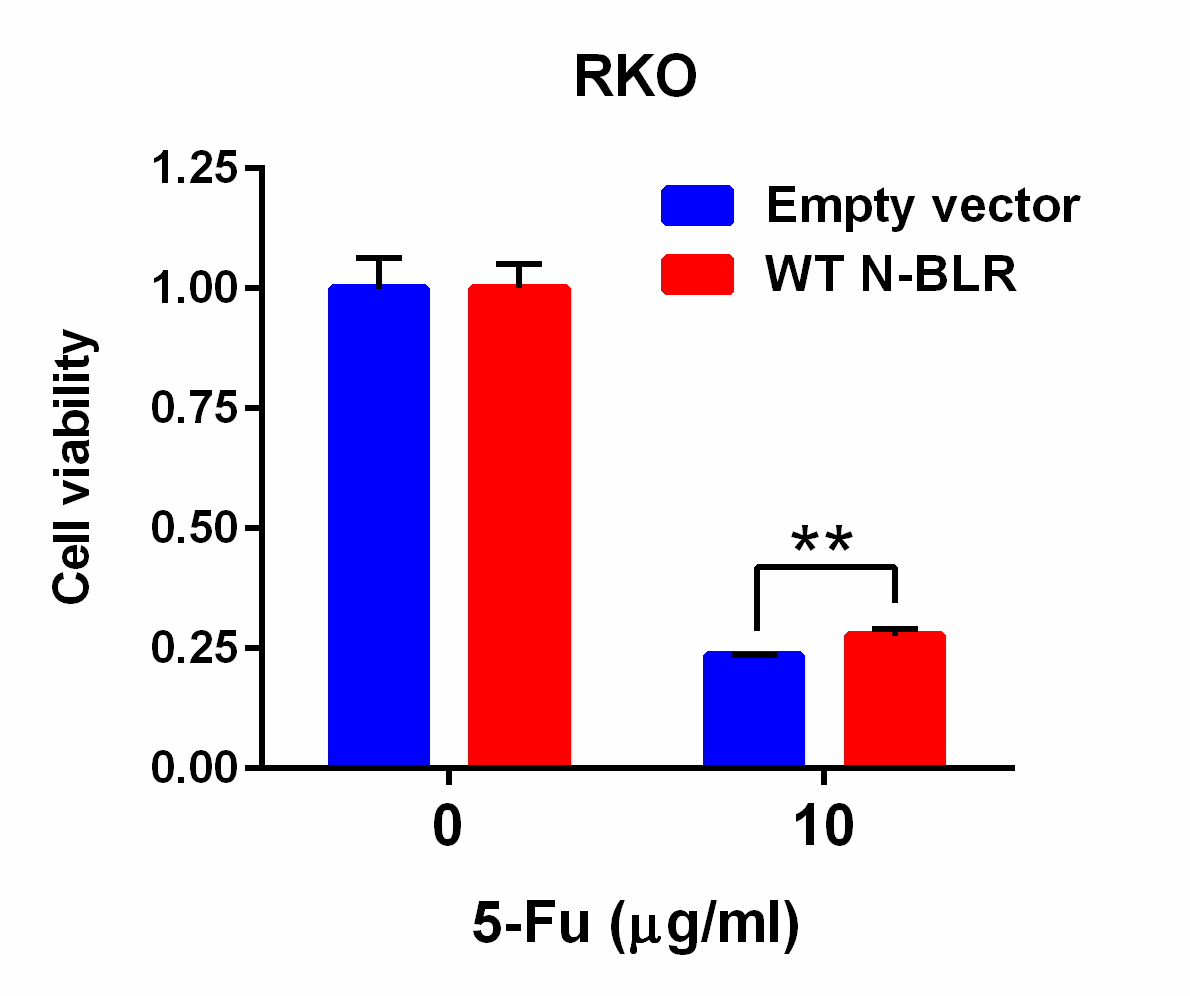

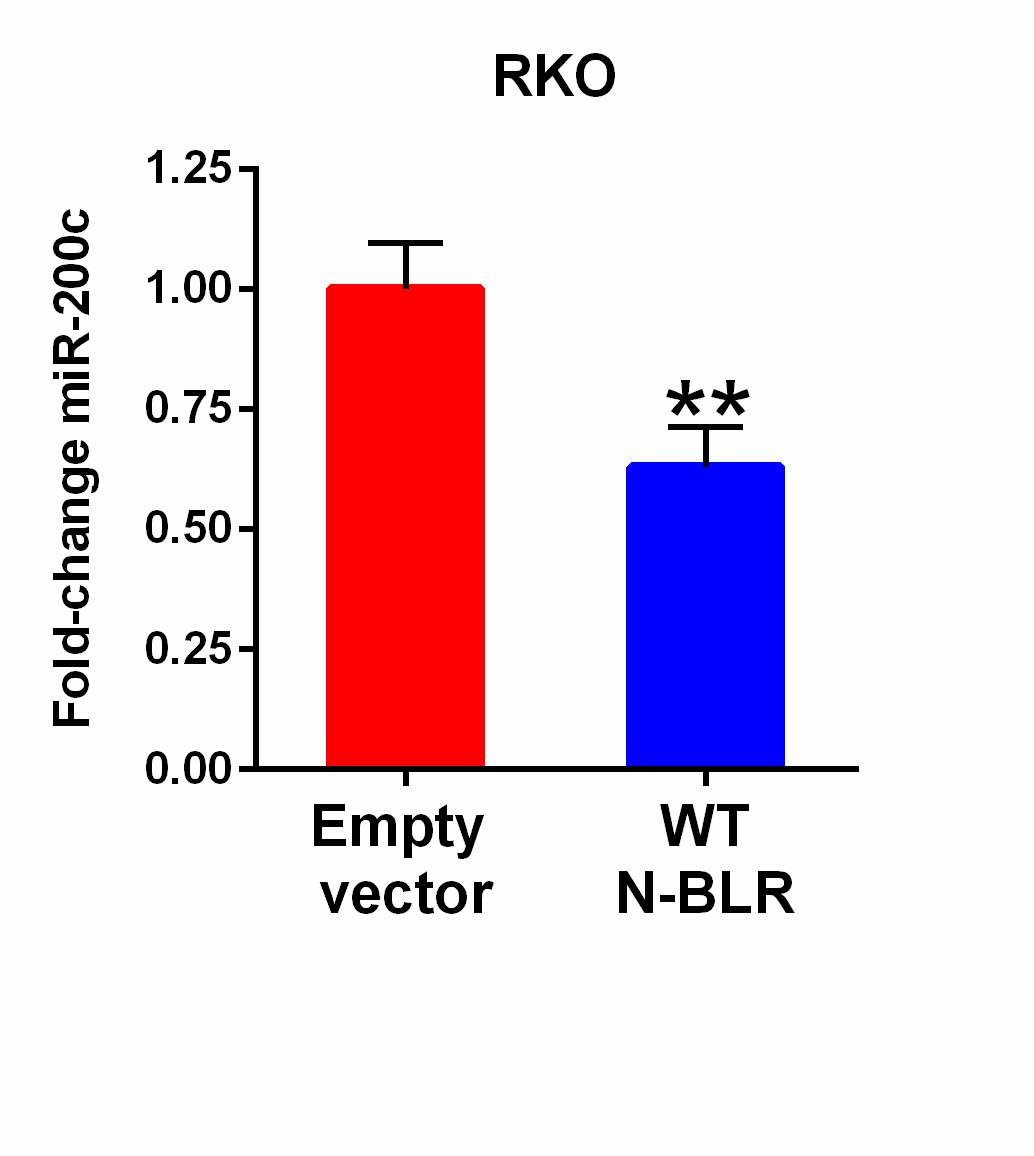
**

**B**

**A**

**
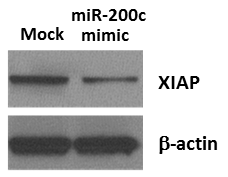
**

**
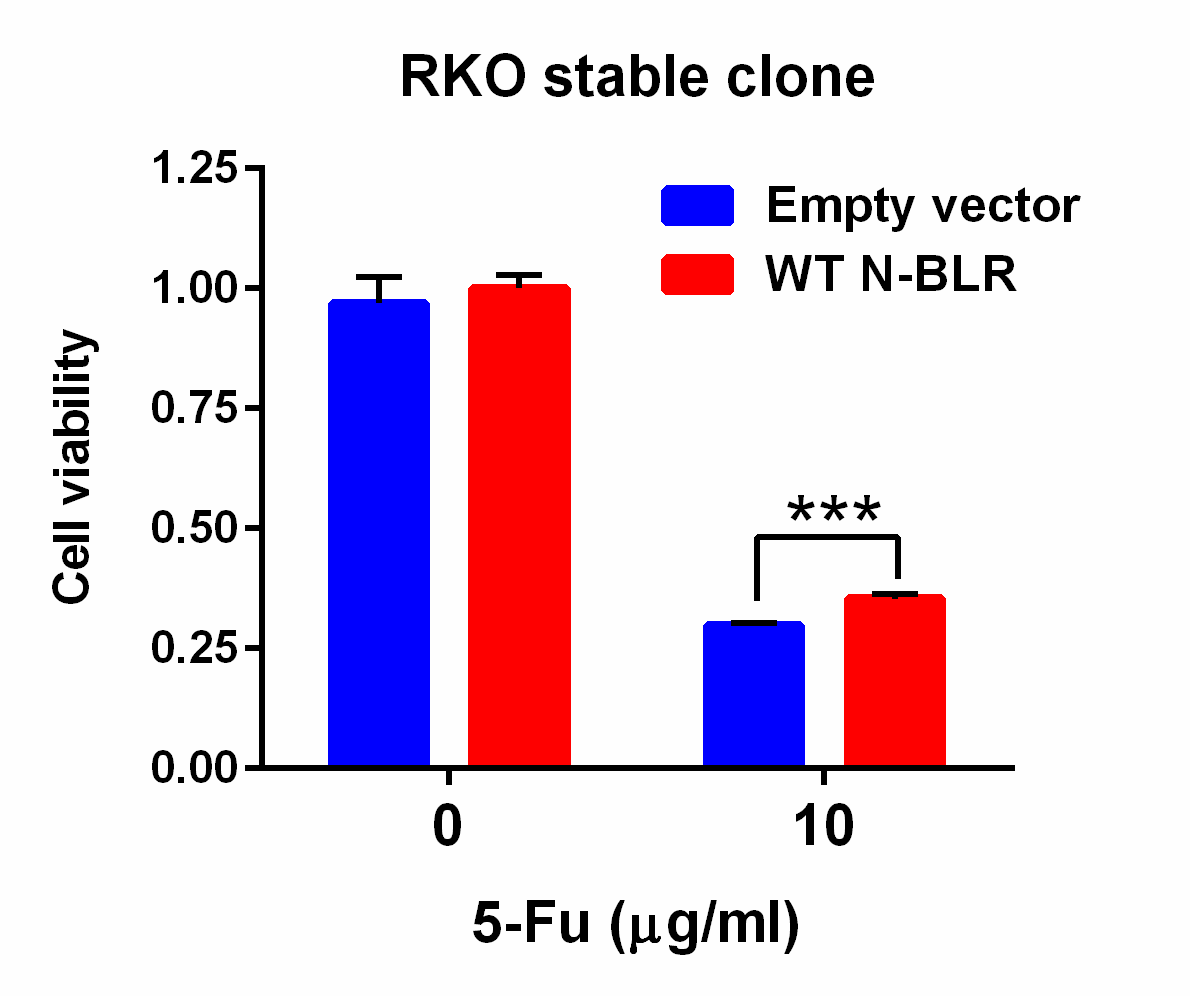
**

**C**

**
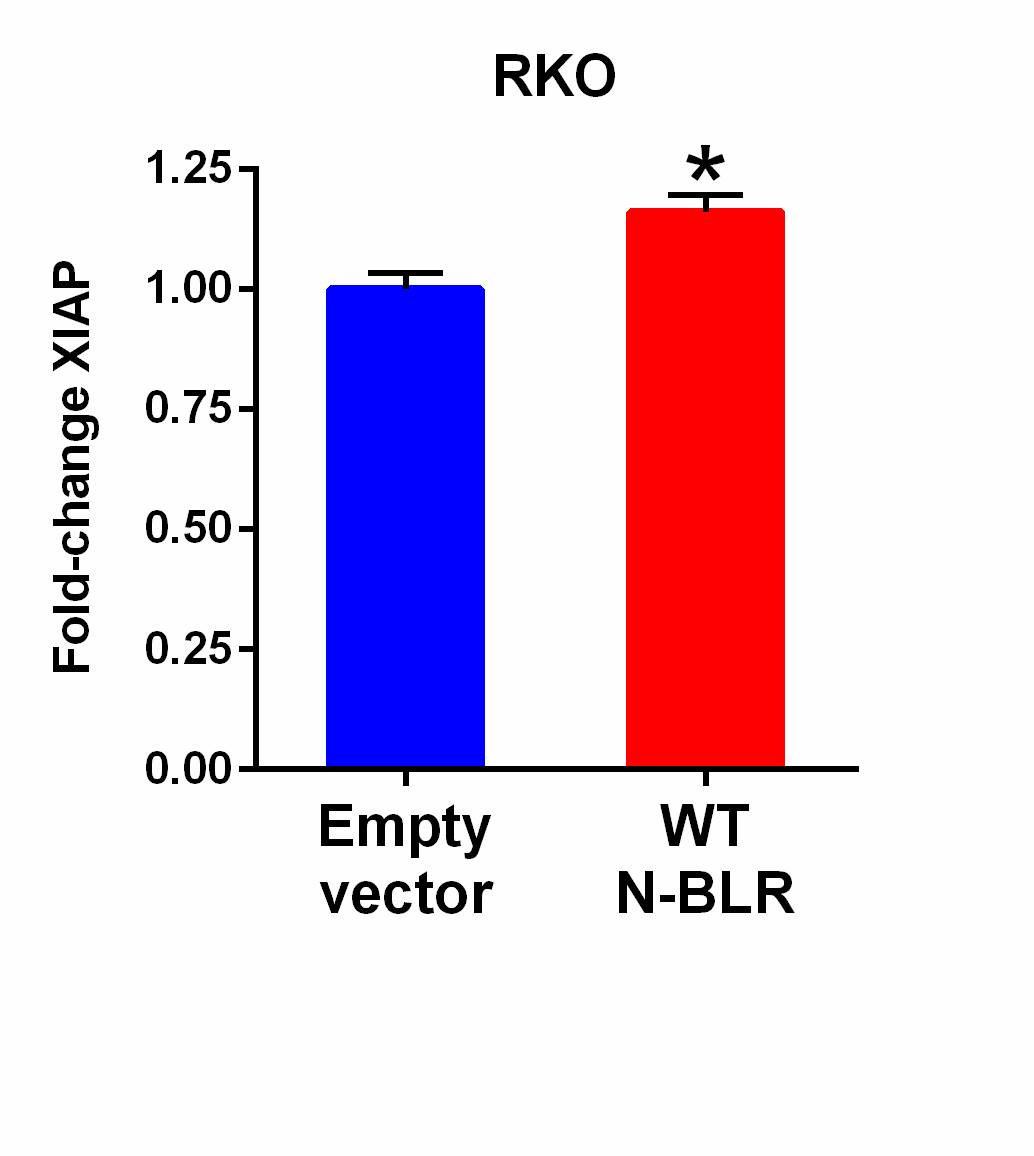
**

**Additional file 3: Fig. S14. The potential effects of 20-nt pyknon motif on N-BLR interaction with miR-200c/miR-141.** **(A)** The upper figure represents the schematic sequence of wild type N-BLR (WT N-BLR) and the location of the pyk-90 motif (red) with respect to the location of the interaction sites for both miRNAs. The first 5 nucleotides (**purple** portion of the shown string) represent the pyk-90 portion overlapping the binding site for miR-200c-3p. The lower figure represents the schematic mutated sequence of N-BLR (pyk90-DEL N-BLR) lacking the whole pyk90 motif, which includes part of the miR-200c-3p binding site. **(B)** Schematic of pcDNA 3.1 (+) vectors including either the full wild type or the pyk-90 deleted N-BLR sequence and deleted vectors at the interaction sites with miR-200c-3p and miR-141-3p were constructed. **(C)** WT and pyk90-DEL N-BLR expression levels were measured in HT-29 cells at 48 h following transient transfection with pcDNA 3.1 overexpressing vectors compared to pcDNA 3.1 empty vector. Y-axis values represent the ratio of N-BLR to U6. Ratios were calculated with the 2^-ΔCt^ method using U6 levels for normalization. **(D)**, **(E)** miR-141-3p expression levels when either WT N-BLR or pyk90-DEL N-BLR, with the correspondent set of deleted vectors, are transfected, respectively. **(F)** Comparison of miR-141-3p expression levels between WT N-BLR and pyk90-DEL N-BLR cells: the binding of miR-141-3p to N-BLR is independent from the presence of the pyk90 motif. Y-axis values represent the ratio of miR-141-3p to U6. Ratios were calculated with the 2^-ΔCt^ method using U6 levels for normalization. For each set of co-transfection experiments, the expression levels of miR-141-3p were corrected by subtracting the values derived from the correspondent miRNA mimic negative control. Data are shown as mean ± SEM. (n=4; Student´s t-test; **p*<0.05; ***p*<0.01; ****p*<0.001).

**F**

**C**


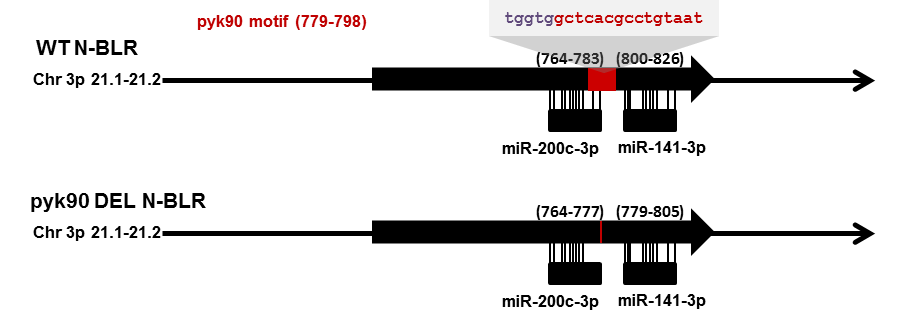


**A**

**B**


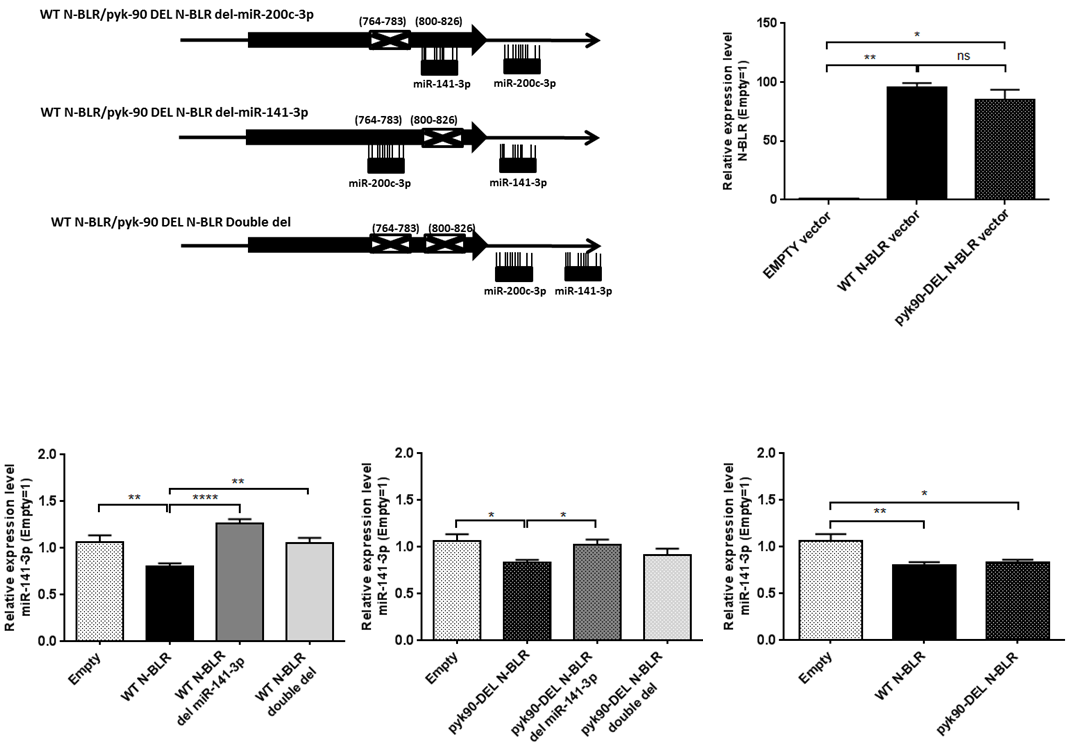


**E**

**D**

**Additional file 3: Fig. S15. N-BLR effects on the post-transcriptional regulation of ZEB1.** **(A)** N-BLR vectors containing WT N-BLR, del miR-200c-3p, del miR-141, and a double deletion (del miR-200c-3p and del miR-141) were transiently transfected in HT-29 cells and compared to cells transfected with the empty vector. At 48 h following transfection, the levels of ZEB1 were assessed by qRT-PCR. Upon N-BLR overexpression the levels of ZEB1 were increased compared with the empty vector control. Data are shown as mean ± SEM. (n=3; Student´s t-test; **p*<0.05). **(B)** RKO cells were transiently transfected with vectors containing wild type N-BLR and empty vector, as control. The expression levels of ZEB1 were measured by qRT-PCR after 48 h of transfection. **(C)** RKO cell were transiently transfected with miR-141-3p and miR-200c-3p mimic (10 nM) for 48 h. The graphic shows the fold-change reduction of ZEB1. Data are shown as mean ± stdev. (n=3; Student´s t-test; **p*<0.05; ***p*<0.01; ****p*<0.001; *****p*<0.0001).


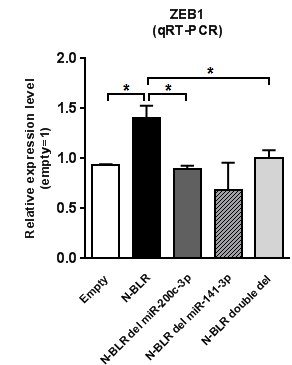


**Relative expression levels ZEB1**

**(Empty vector=1)**

**HT-29**

**A**

**
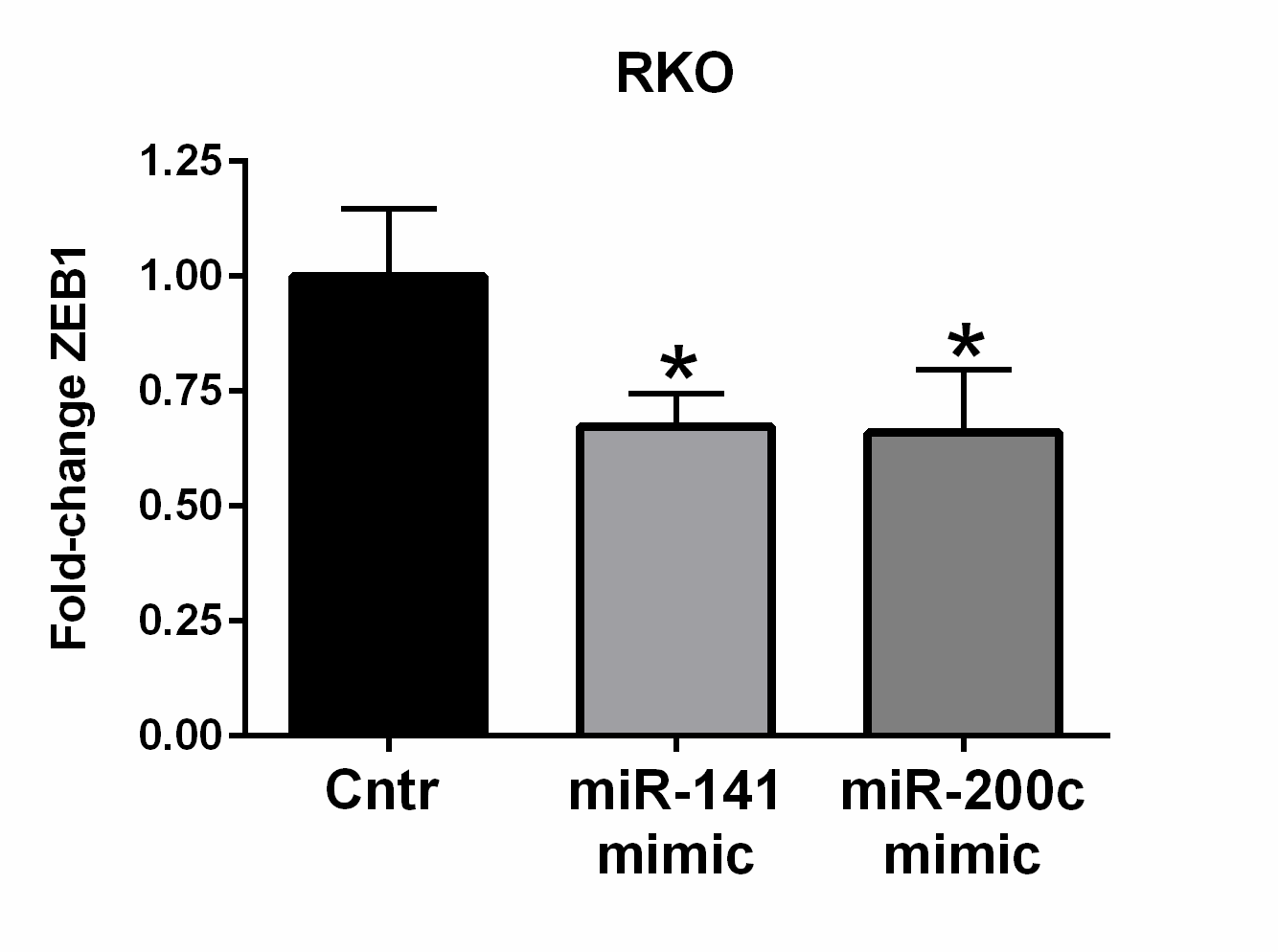

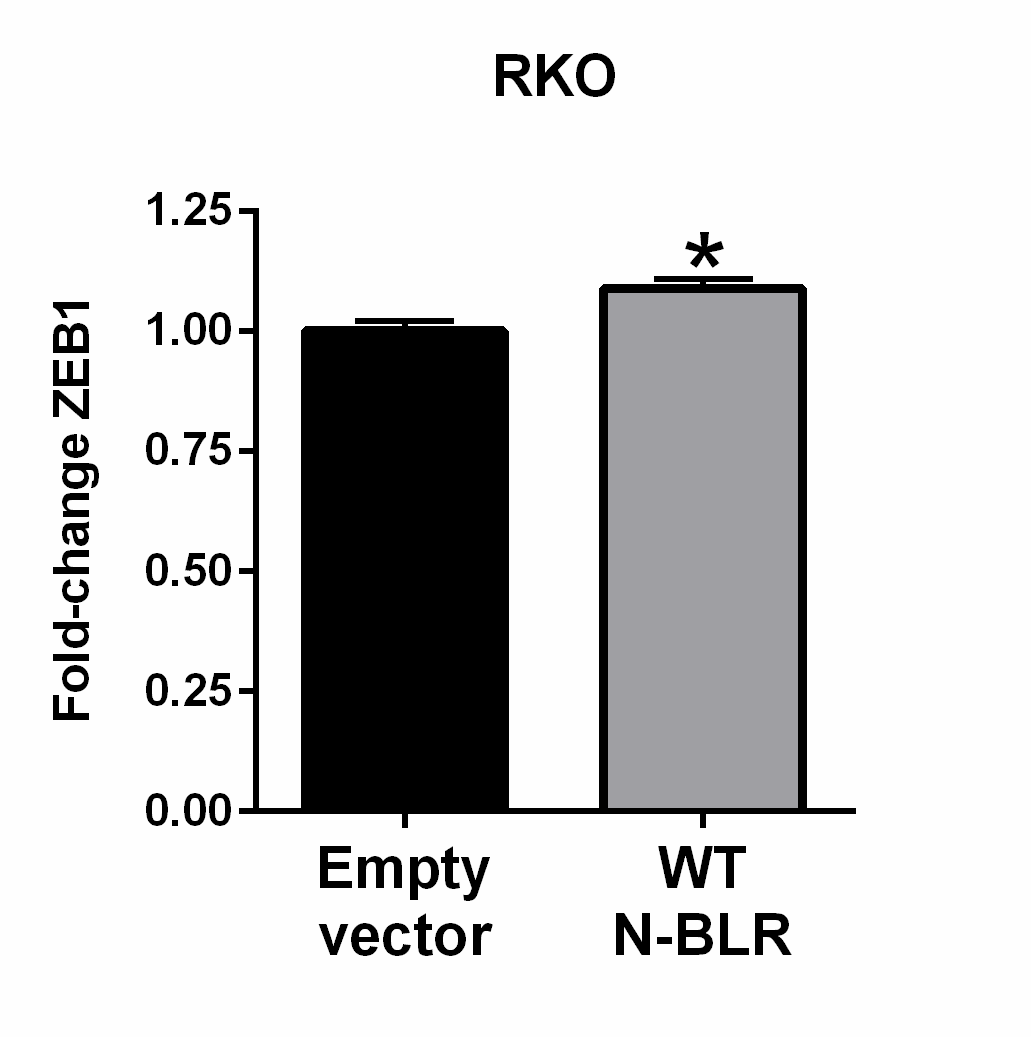
**

**B**

**C**

**Additional file 3: Fig. S16. Rescue of the malignant phenotype by the deletion of the 20-nt pyknon motif from the 845-nt N-BLR clone**. **(A)** HCT116 cells were transiently transfected with WT N-BLR, pyk90-DEL N-BLR and Empty vectors. After 24 h of transfection, 2.5x10^4^ HCT116 cells /insert were seeded (membrane pore size 8mM) in 100ml serum free medium (Left panel-Migration), or onto the inserts pre-coated with a 50ml layer of 300mg/ml matrigel (Right panel-Invasion). The transwell inserts were incubated overnight in a 24-well plate containing 0.6 ml complete medium. The cells that moved to the bottom side of the insert membranes were fixed, stained, and analyzed compared to the controls. The white dots represent the pores of the membranes, while the cells are the violet dots (magnification 200x). **(B)** The transfected HCT116 cells were seeded as 500 cells / 6cm-dish (Left panel), or 500 cells/well in a 6-well plate containing 0.4% soft agar for 2 weeks. The cell colonies were analyzed compared to the controls. Data are shown as mean ± SEM. (n=3; Student´s t-test; **p*<0.05; ***p*<0.01). Solid arrow indicates cells; dashed arrow indicates insert membrane pores.


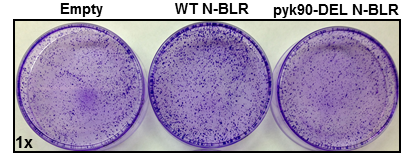

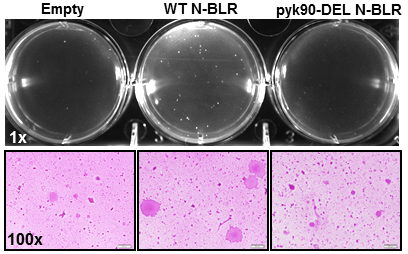

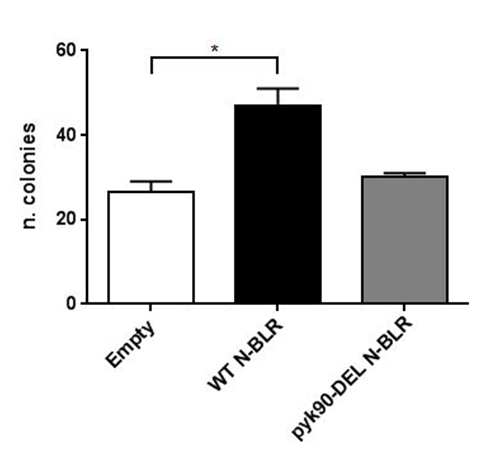

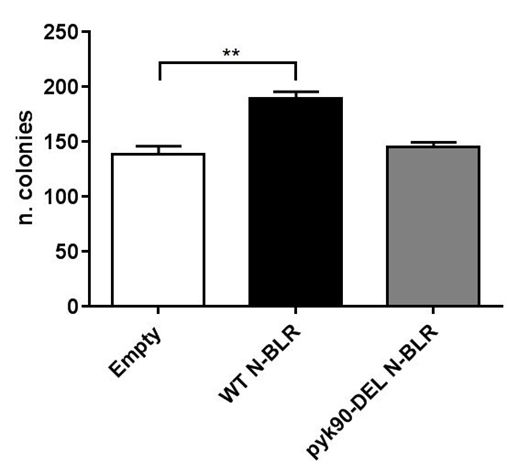

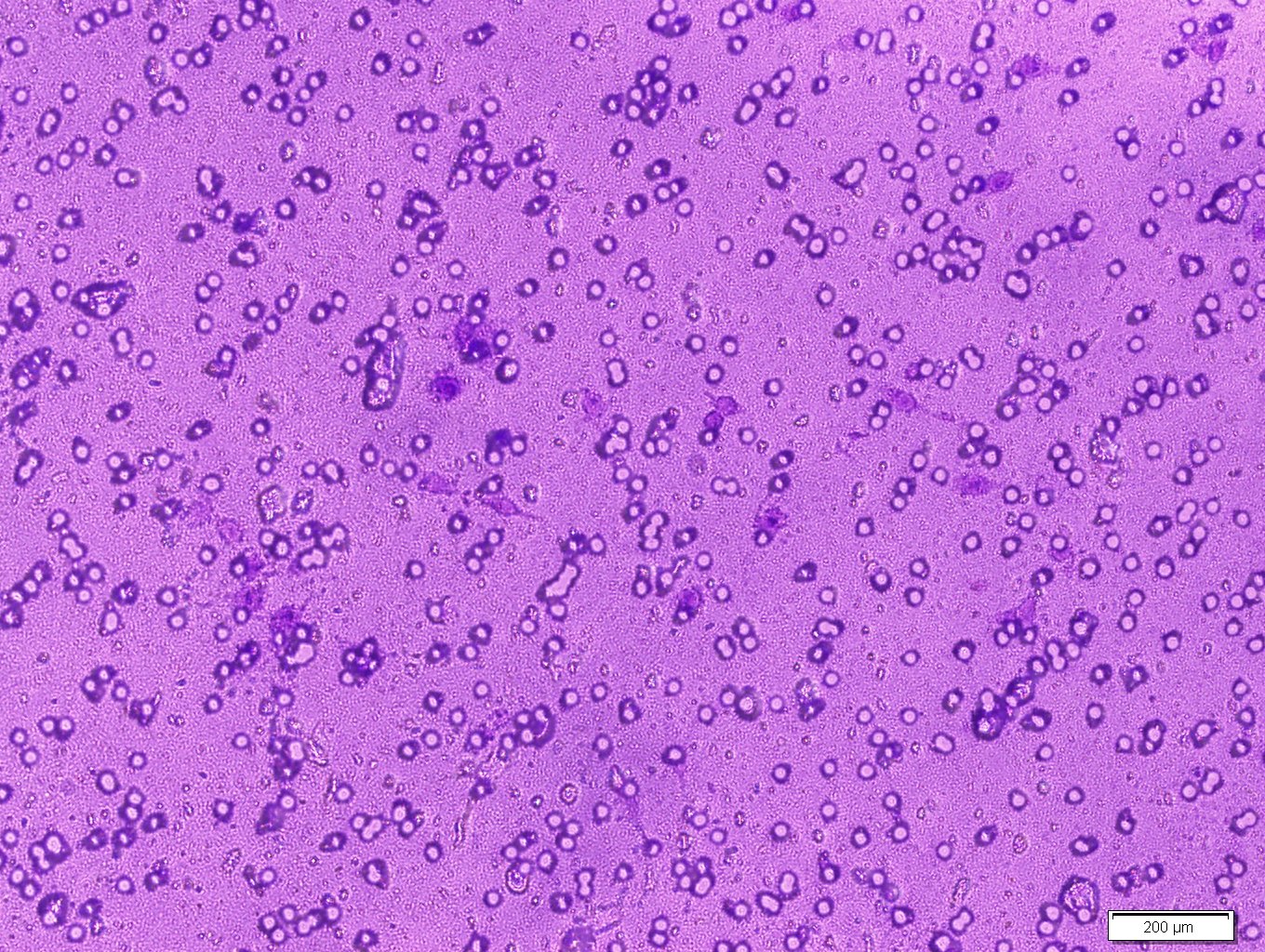

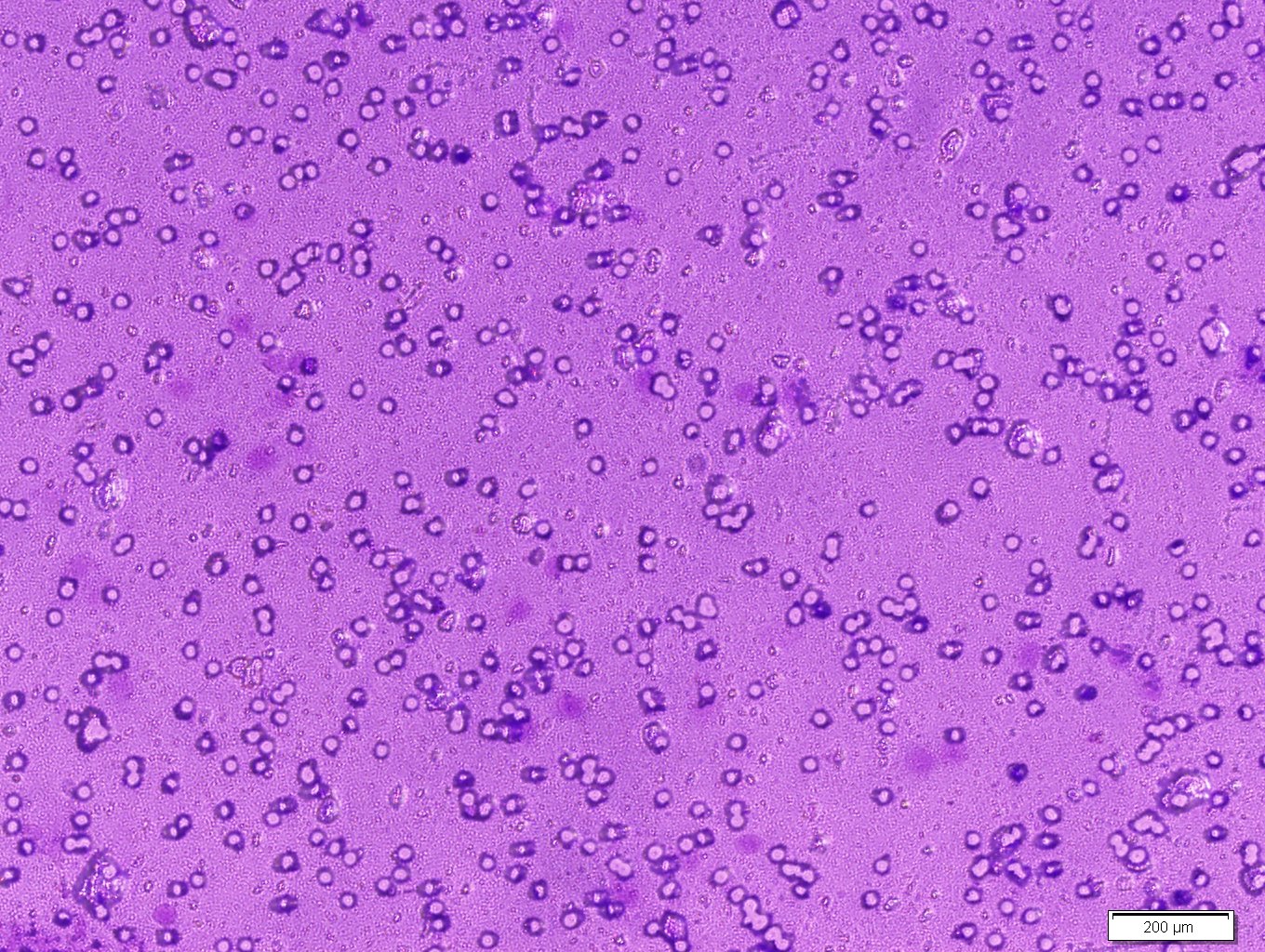

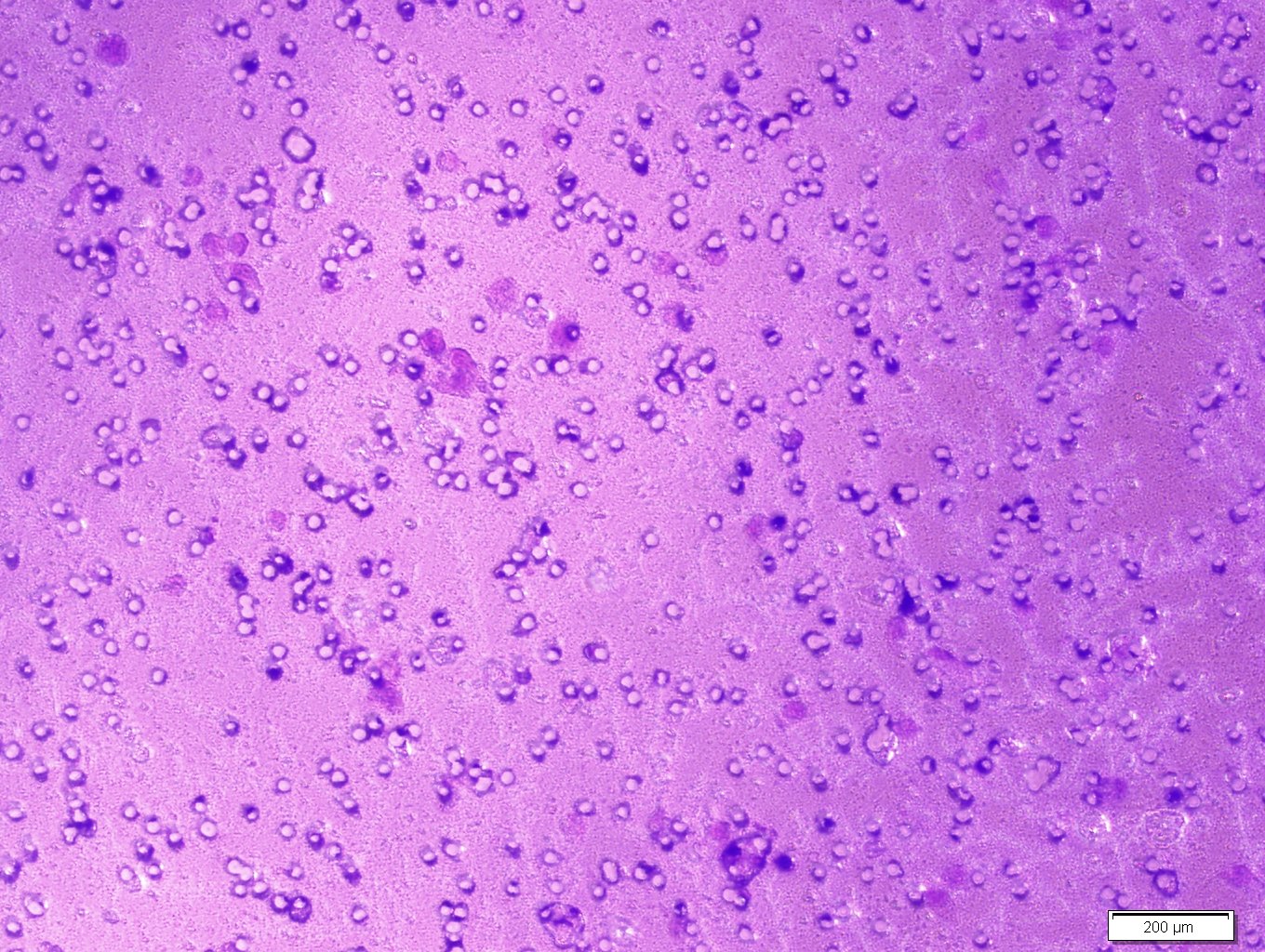

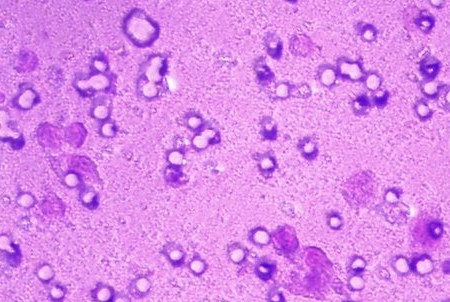

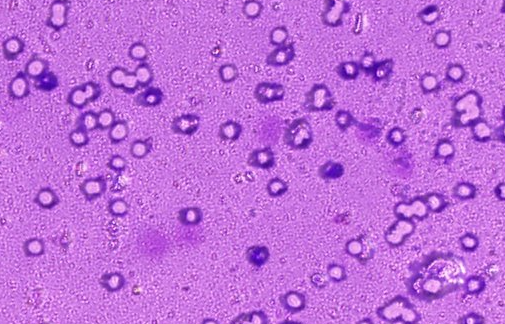

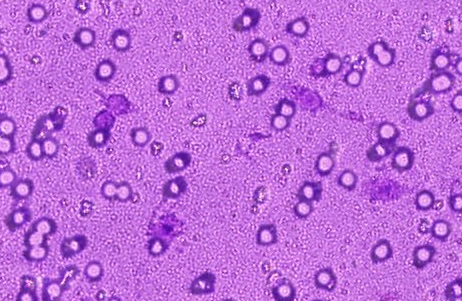


**A**

**Relative cell migration (%)**

**pyk90-DEL**

**N-BLR**

**WT**

**N-BLR**

**Empty**

**vector control**

**Relative cell invasion (%)**

**WT**

**N-BLR**

**Empty**

**vector control**

**pyk90-DEL**

**N-BLR**


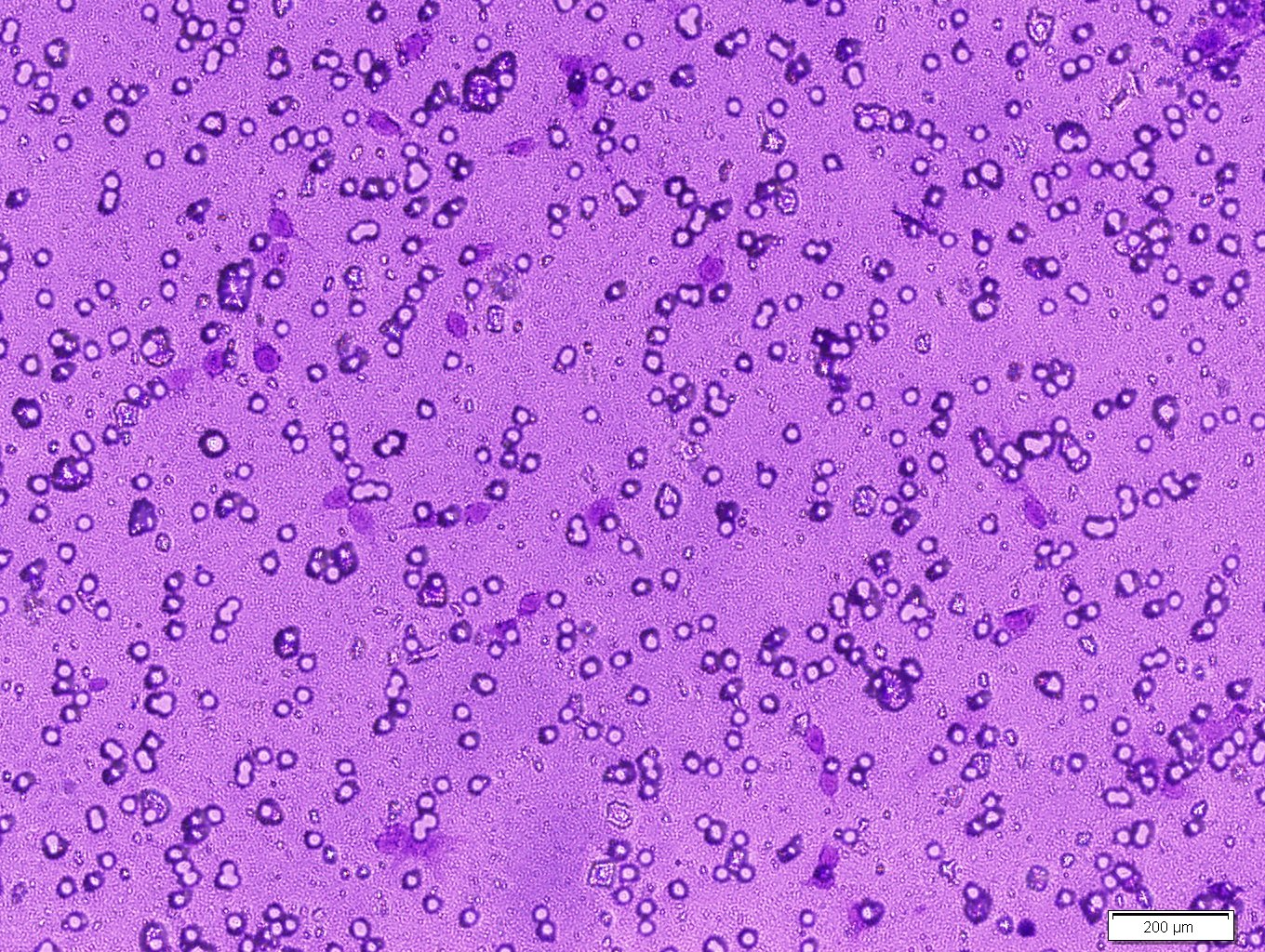

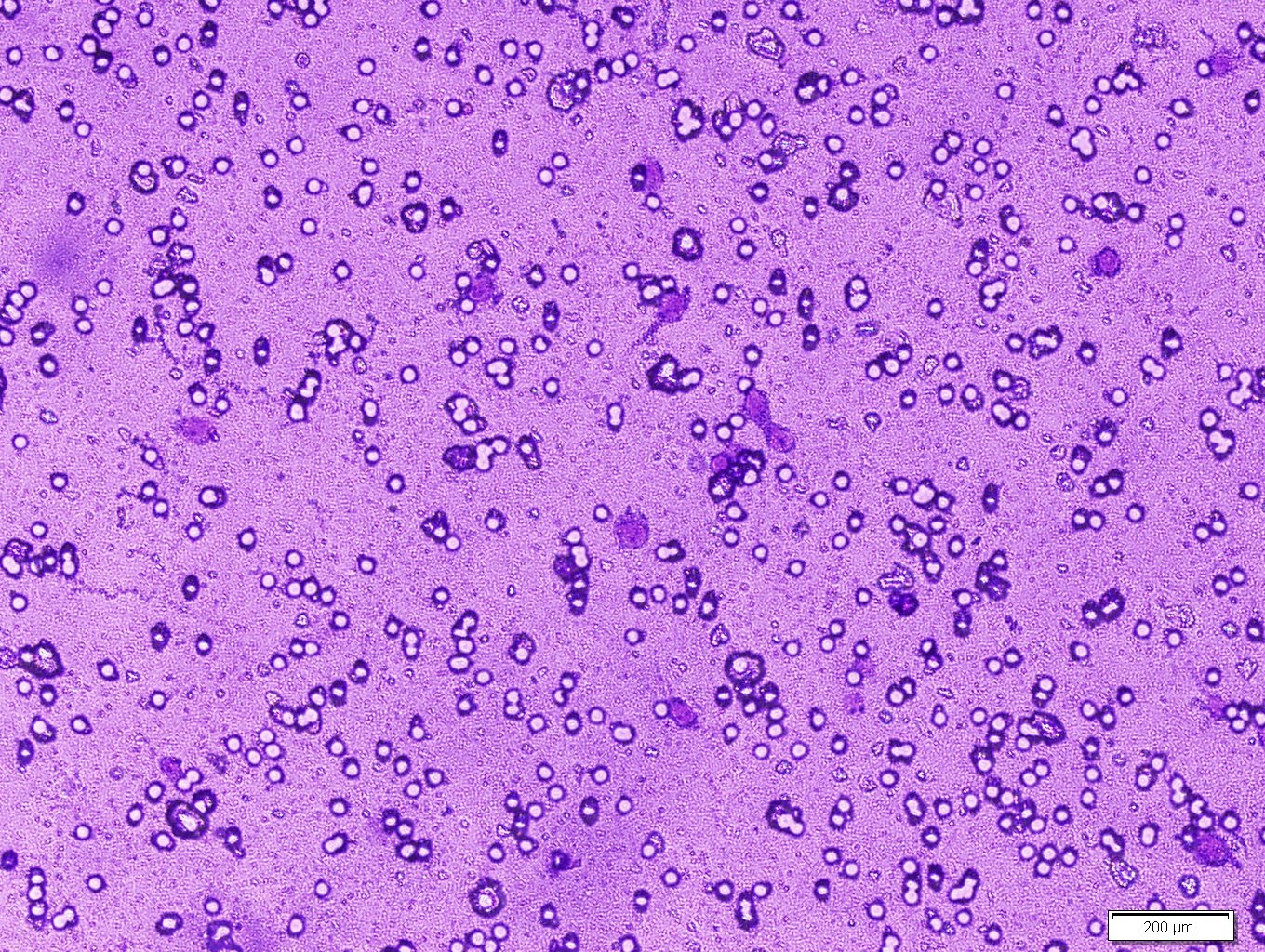

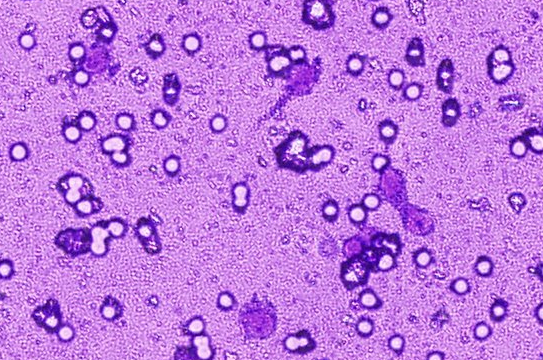

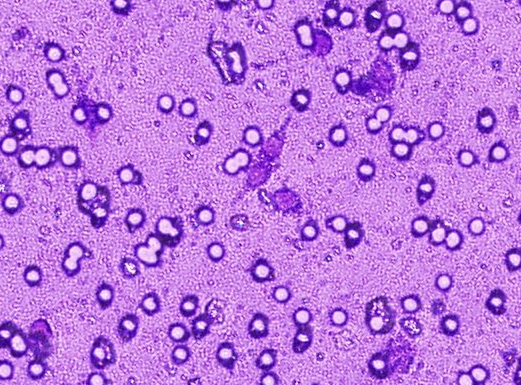

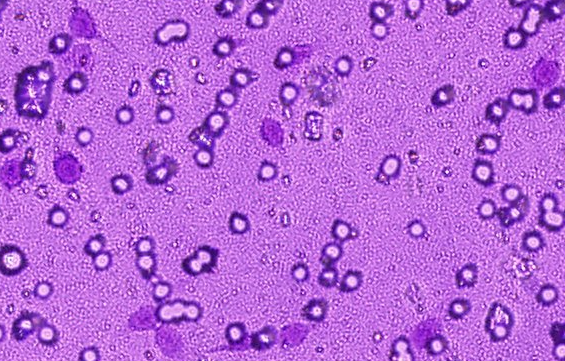

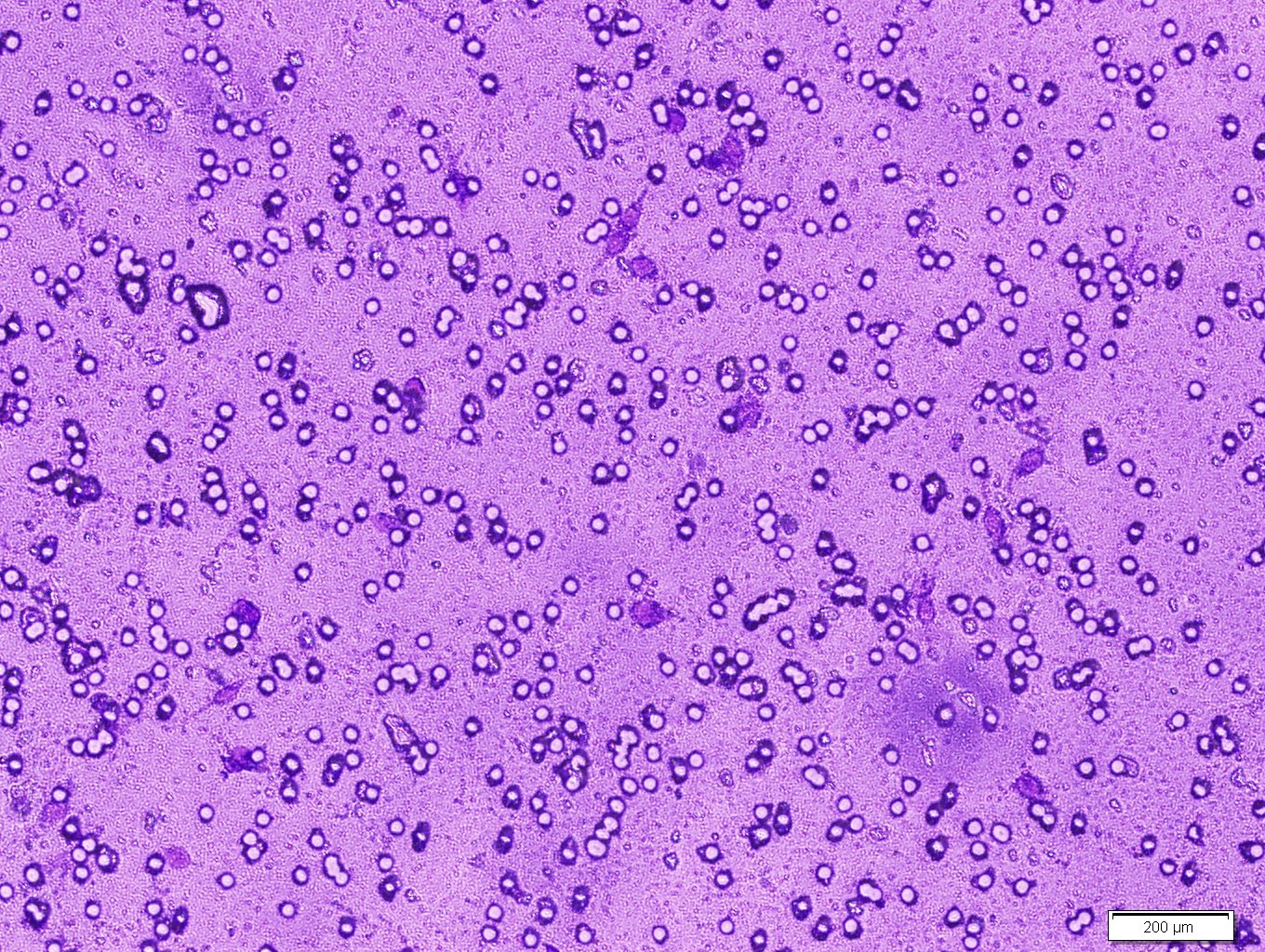


*

*

**B**

**Additional file 3: Fig. S17. Effect of deletion of pyk90 sequence between the binding sites of miR-141-3p and miR-200c-3p (DEL2) on migration and invasion.** RKO **(A)** and HCT116 **(B)** cells were transiently transfected with vectors containing N-BLR sequences: wild type (WT N-BLR); wild type lacking part of the miR-200c-3p binding site (pyk90-DEL N-BLR); wild type with the deletion spanning only the portion of the pyknon that is between the miR-141-3p and miR-200c-3p binding sites (pyk90-DEL2 N-BLR). RKO cells transfected with empty vector were used as control. Migration (left) and invasion (right) experiments were conducted in triplicate and the results were normalized by proliferation rate of each cell type. Data are shown as mean ± stdev. (n=3; Student´s t-test; *p<0.05; **p<0.01; ***p<0.001; ****p<0.0001).

**A**


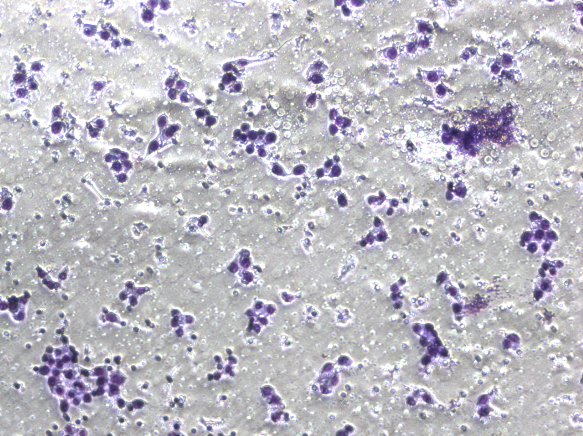


**pyk90-DEL2 N-BLR**


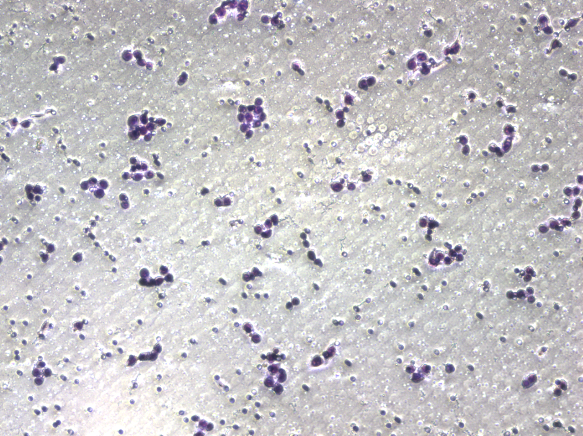


**Empty vector**


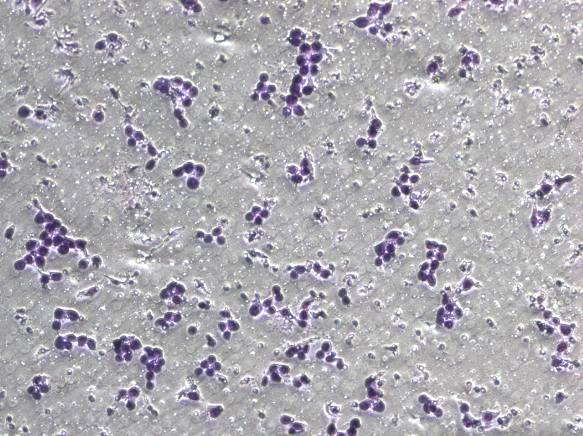


**WT N-BLR**


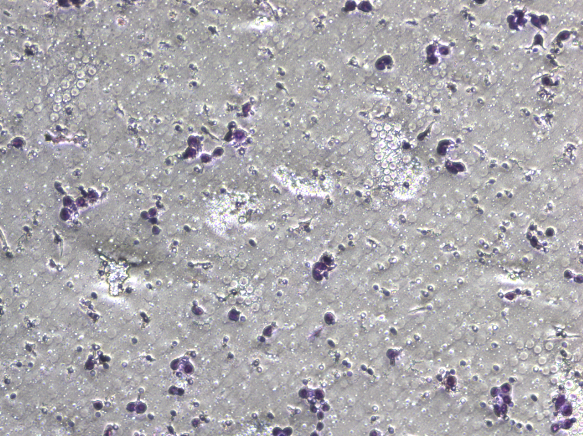


**pyk90-DEL N-BLR**


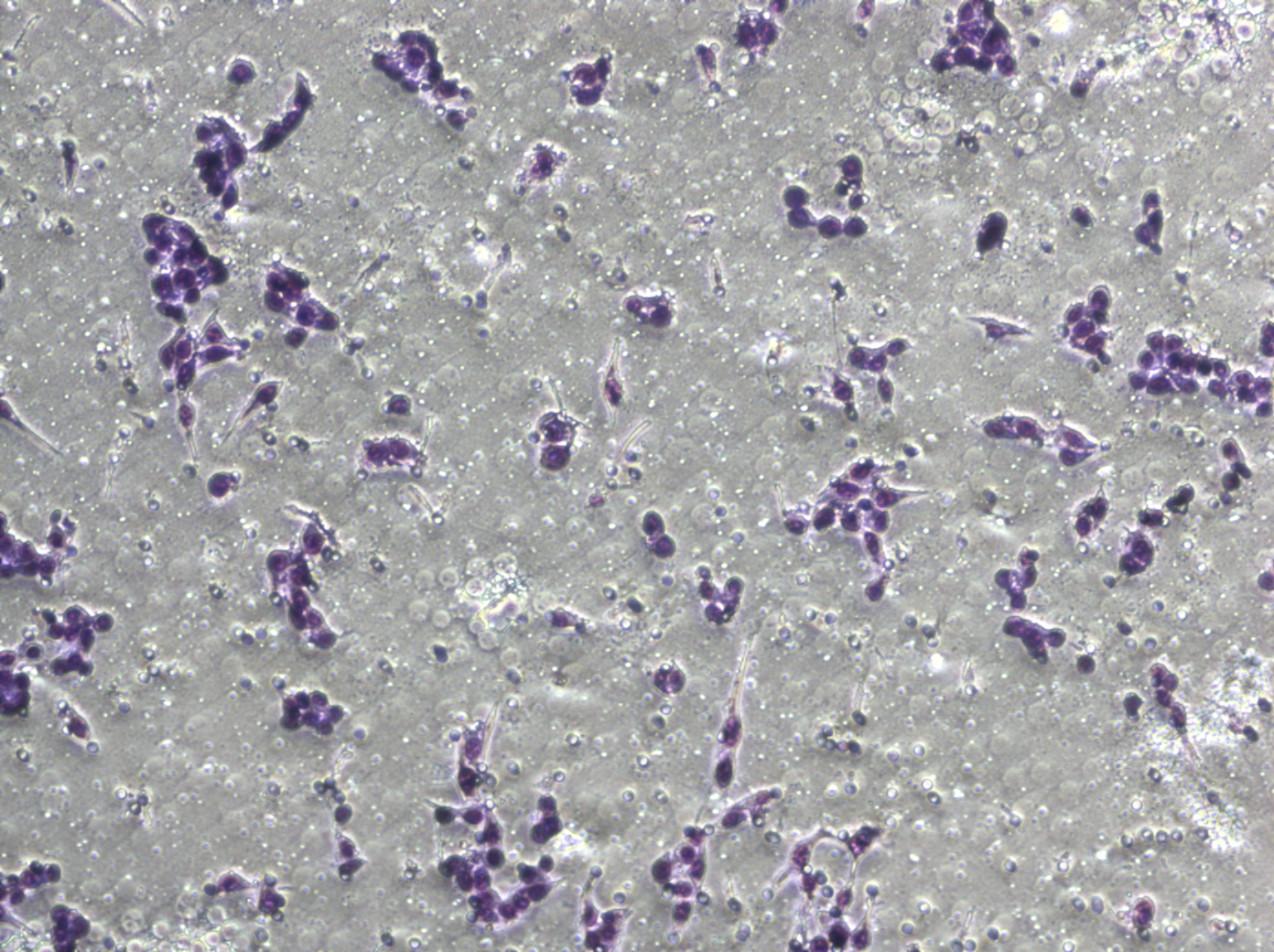

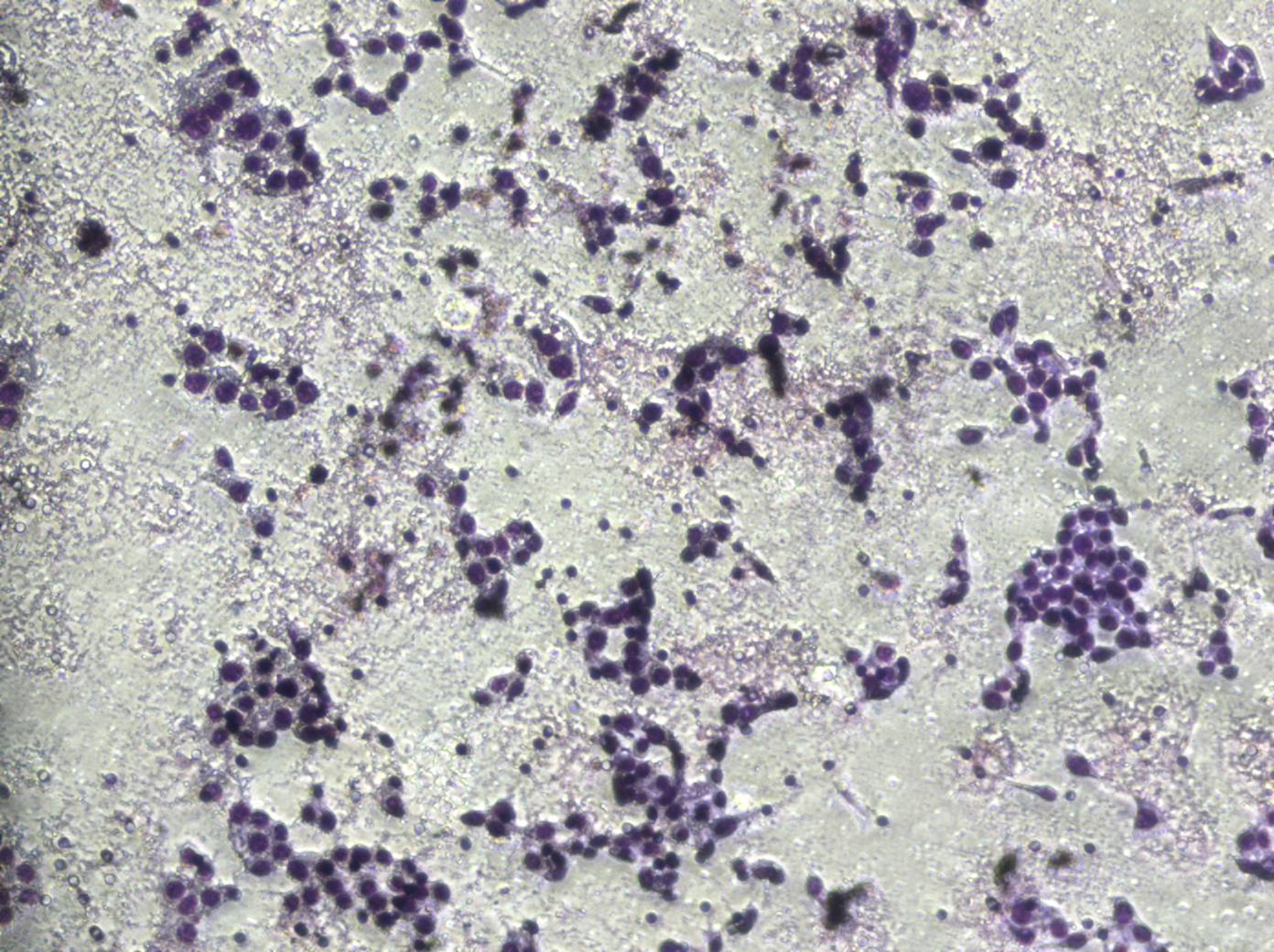

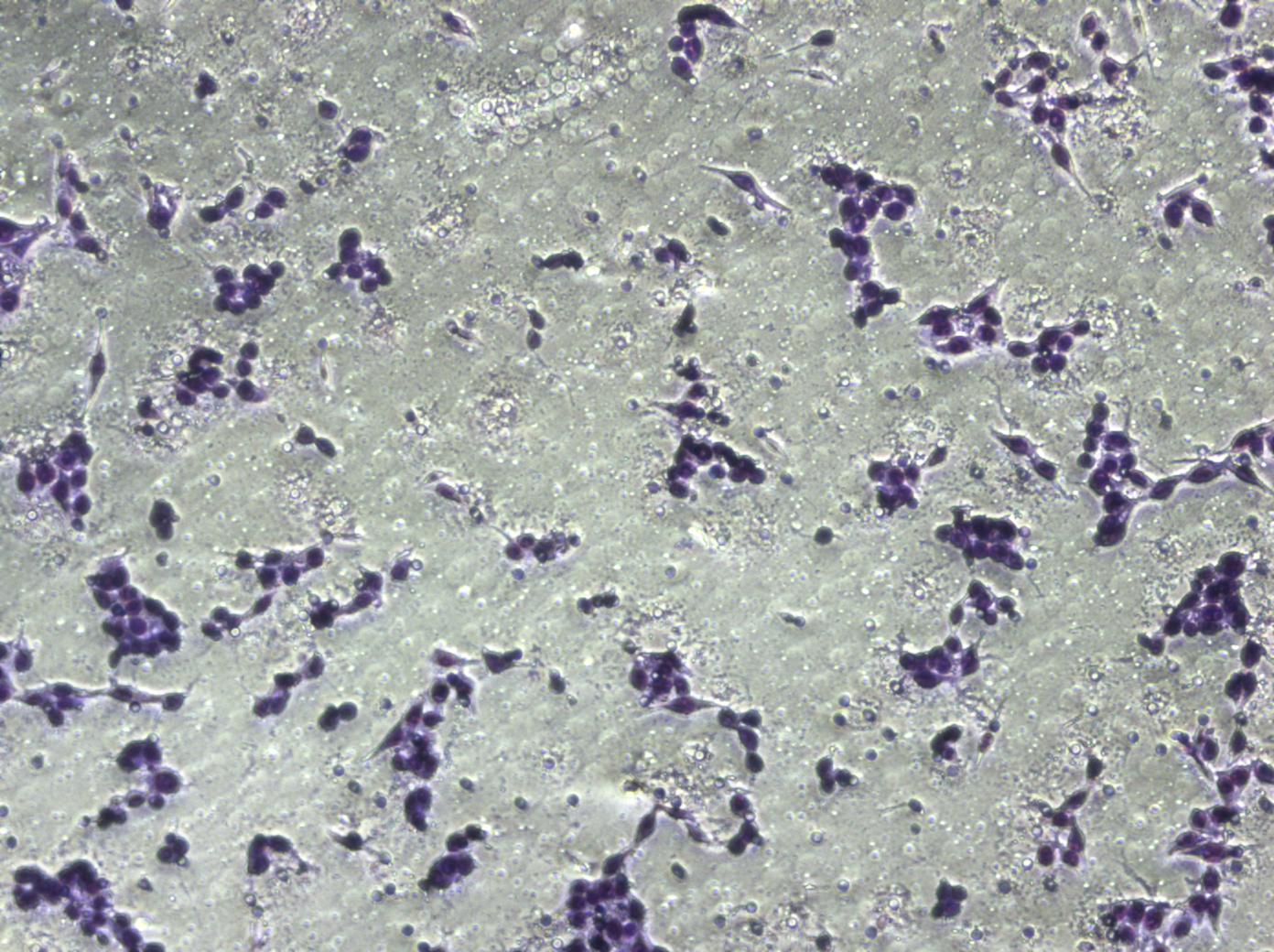

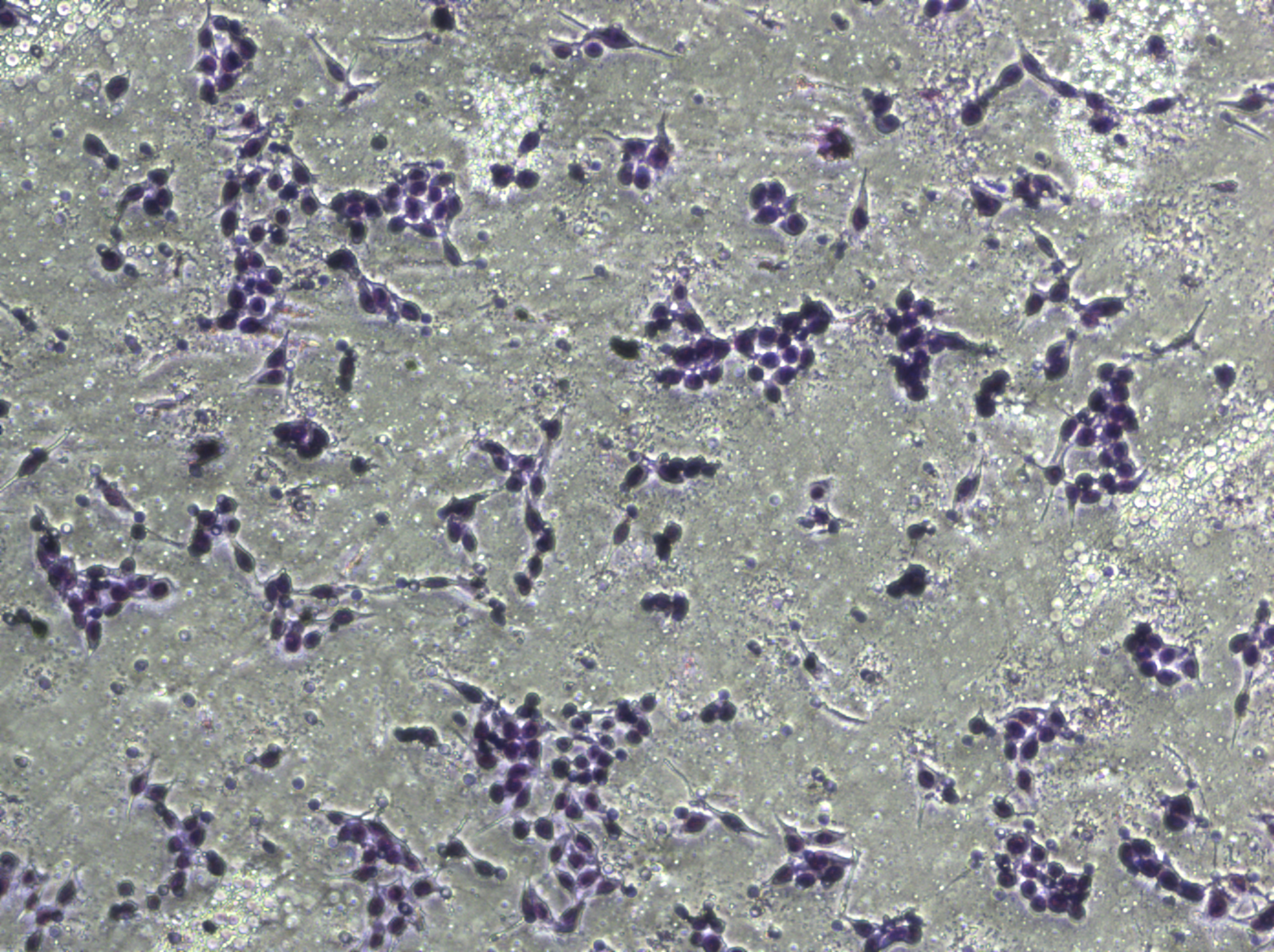


**pyk90-DEL2 N-BLR**

**Empty vector**

**WT N-BLR**

**pyk90-DEL N-BLR**


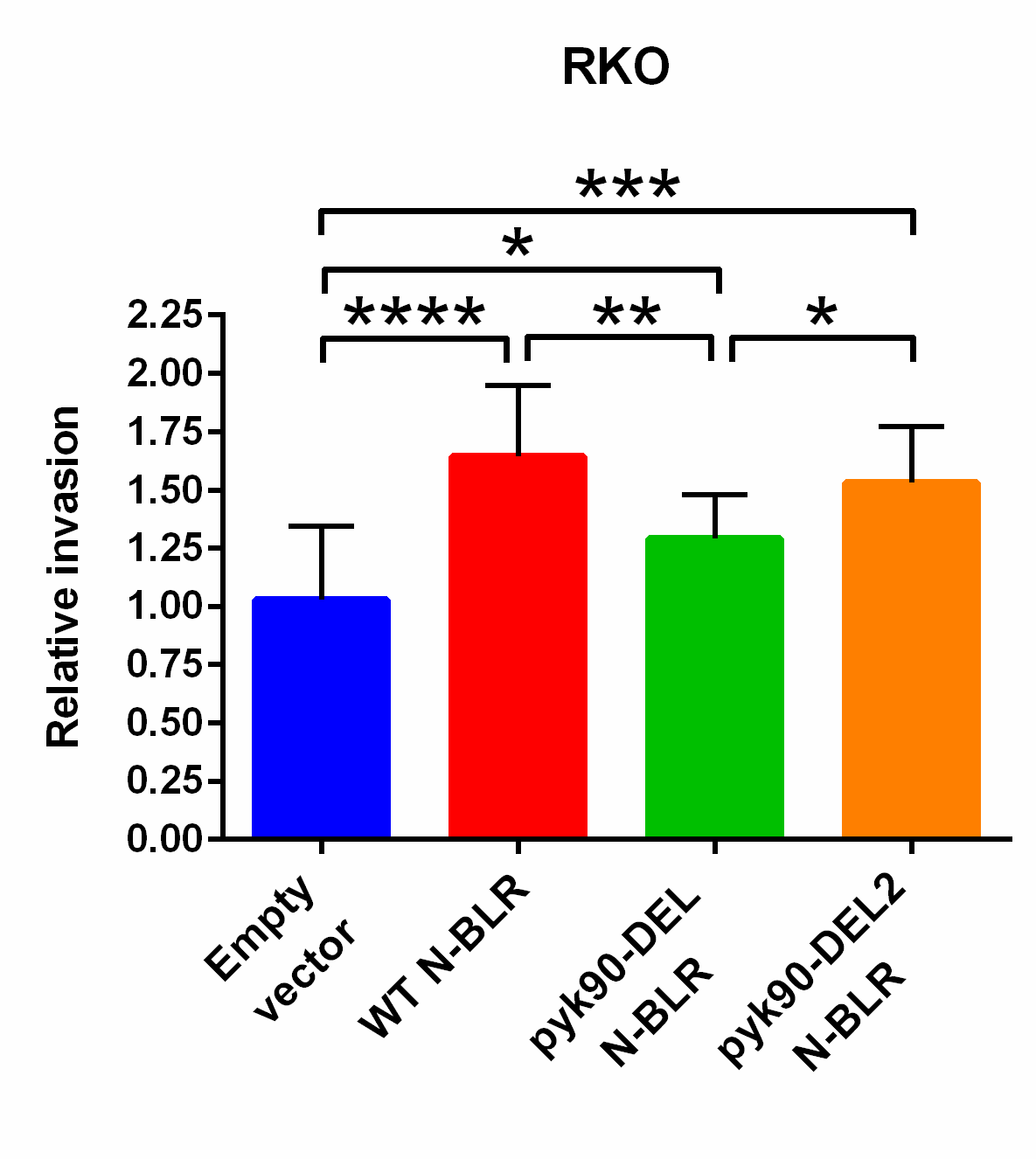

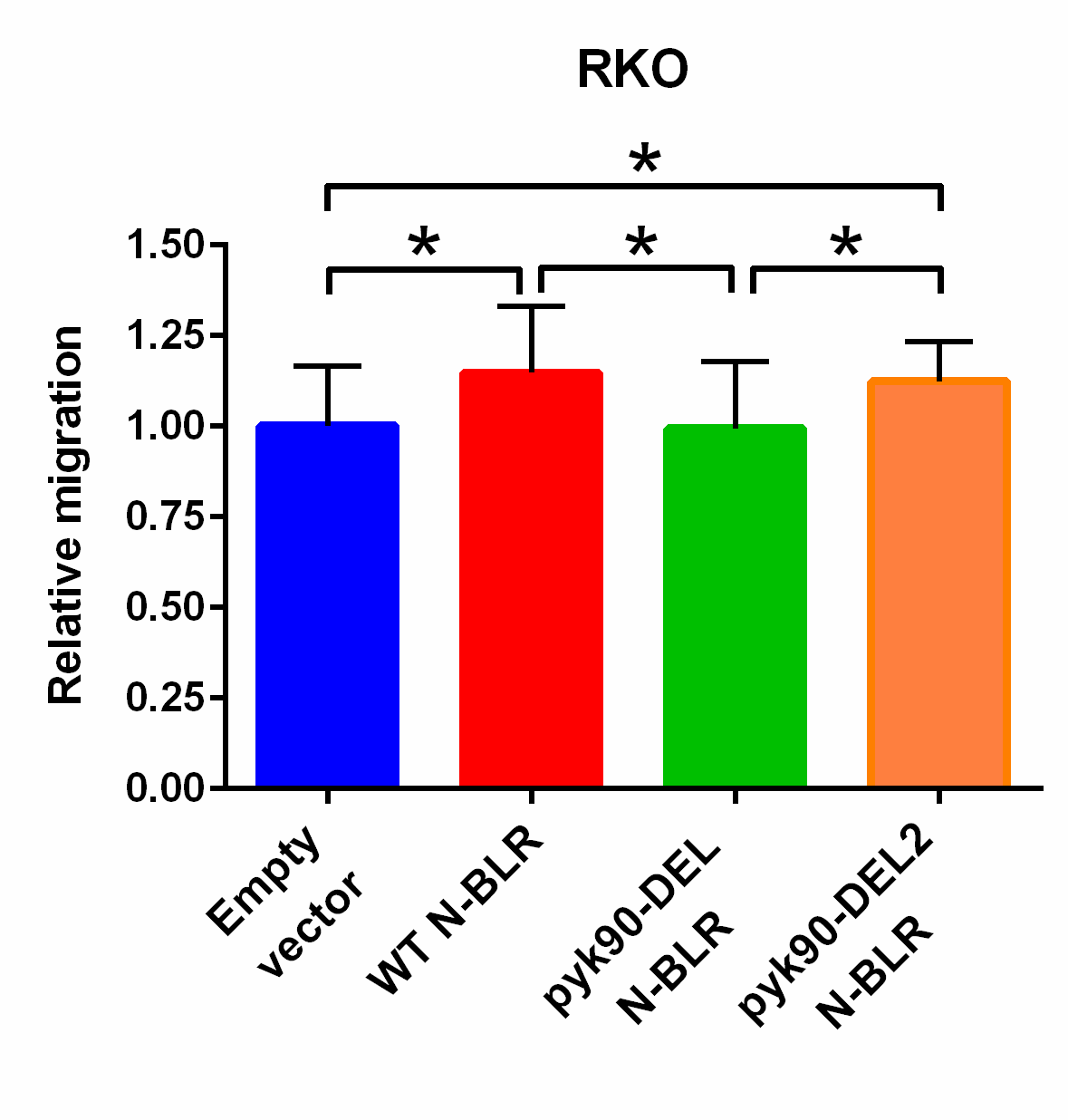


**Additional file 3: Fig. S17 (Continued).**

**B**

**
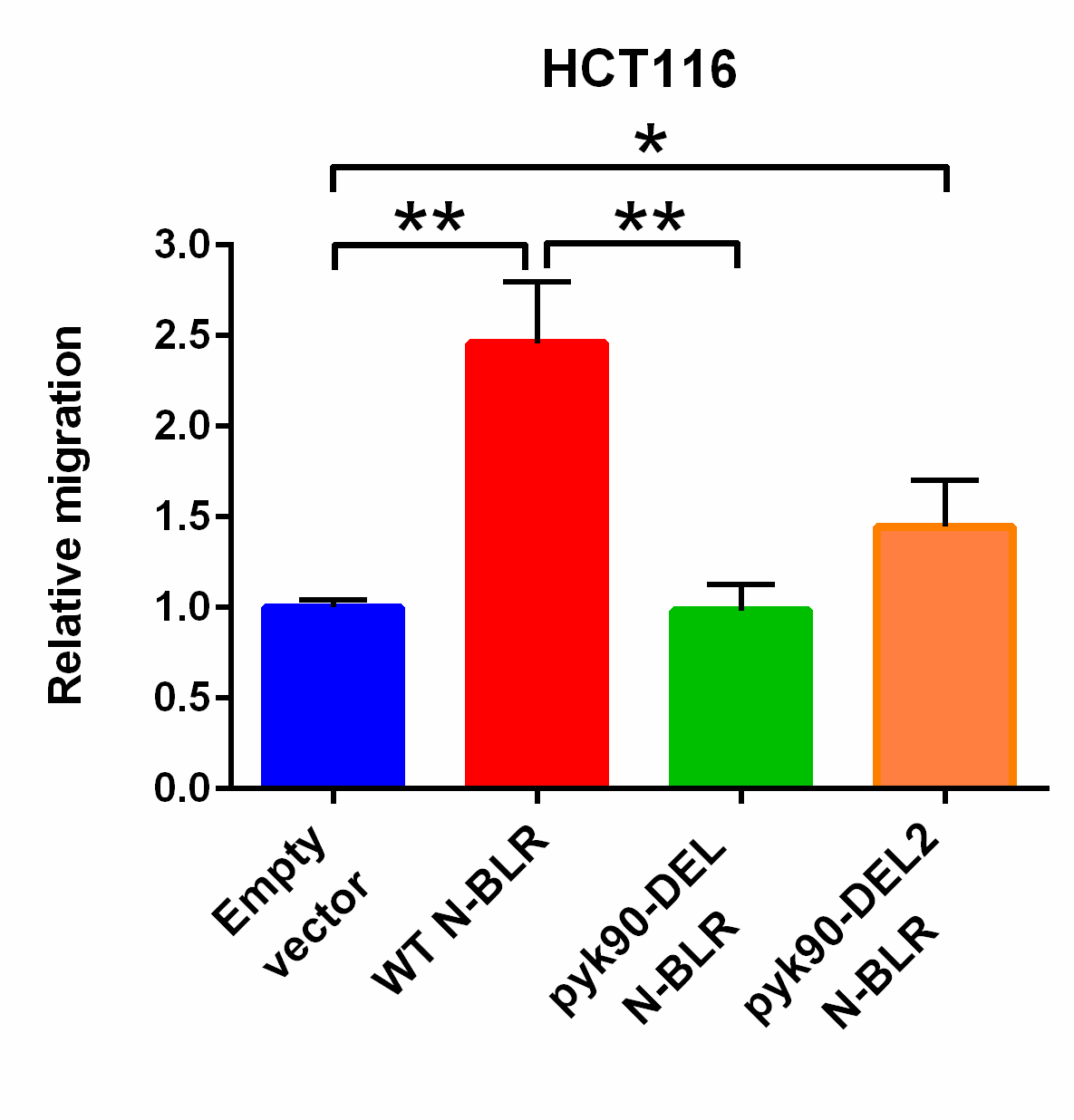

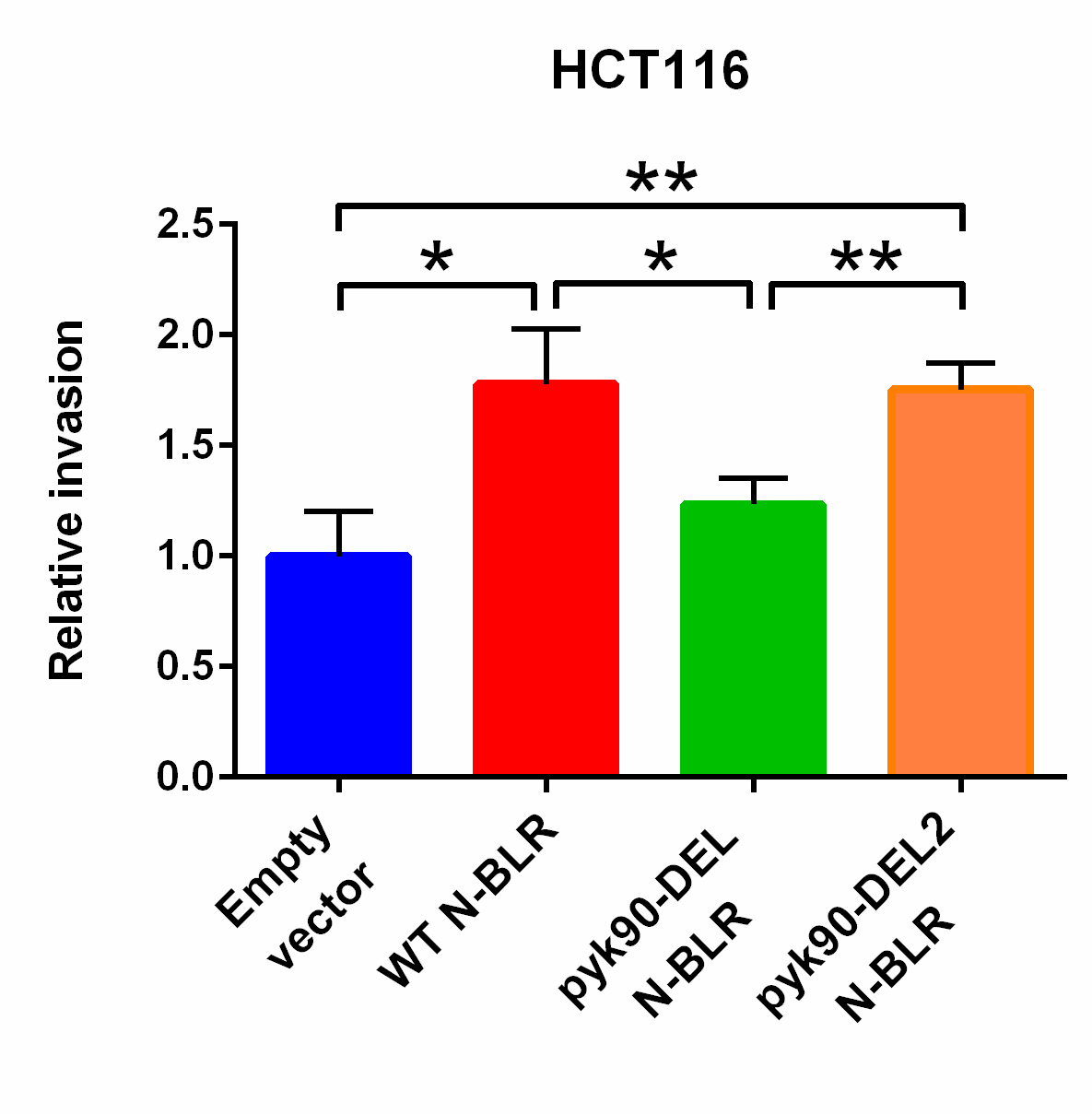
**

**Empty vector**

**WT-NBLR**

**Empty vector**

**WT-NBLR**


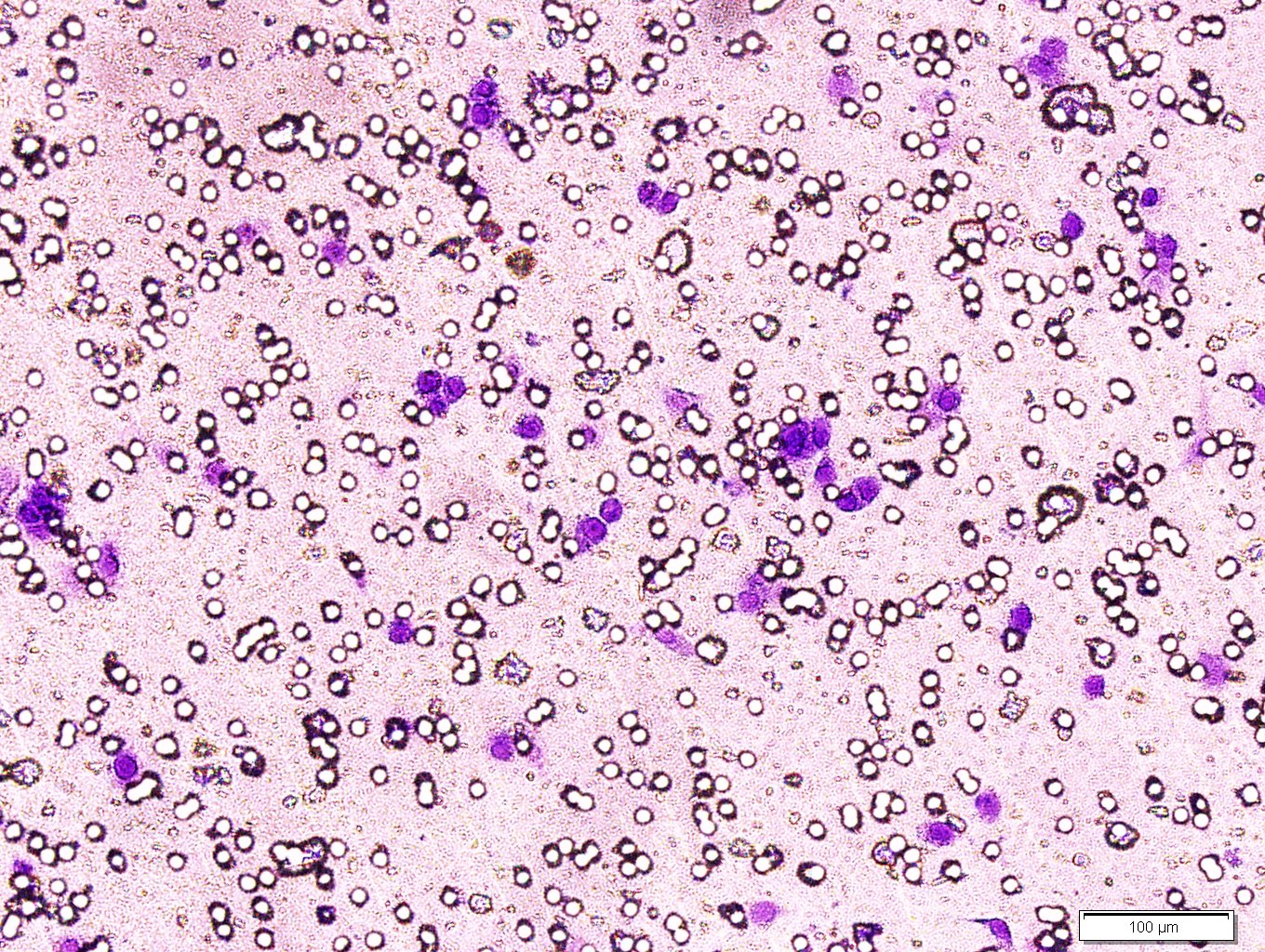

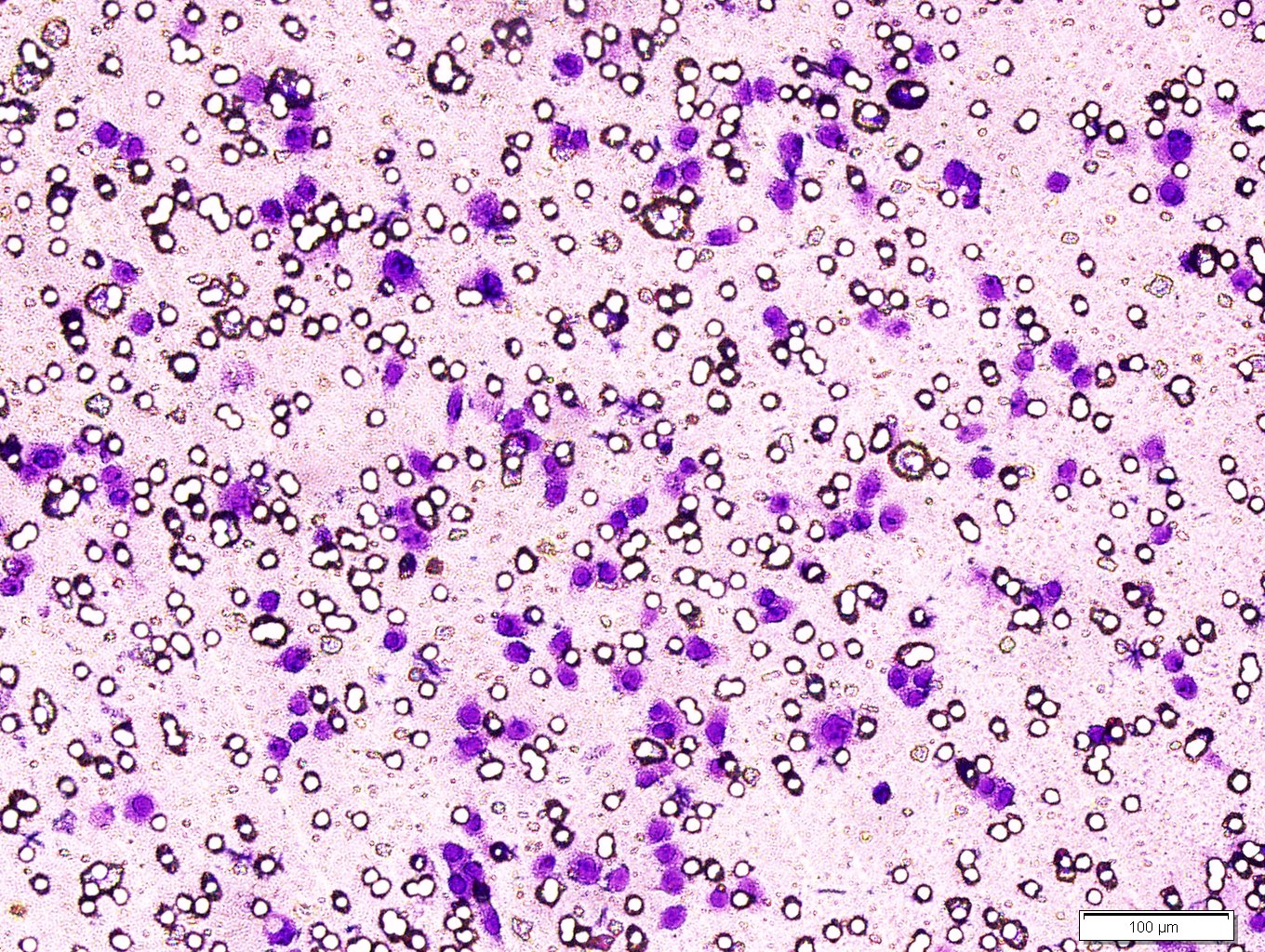

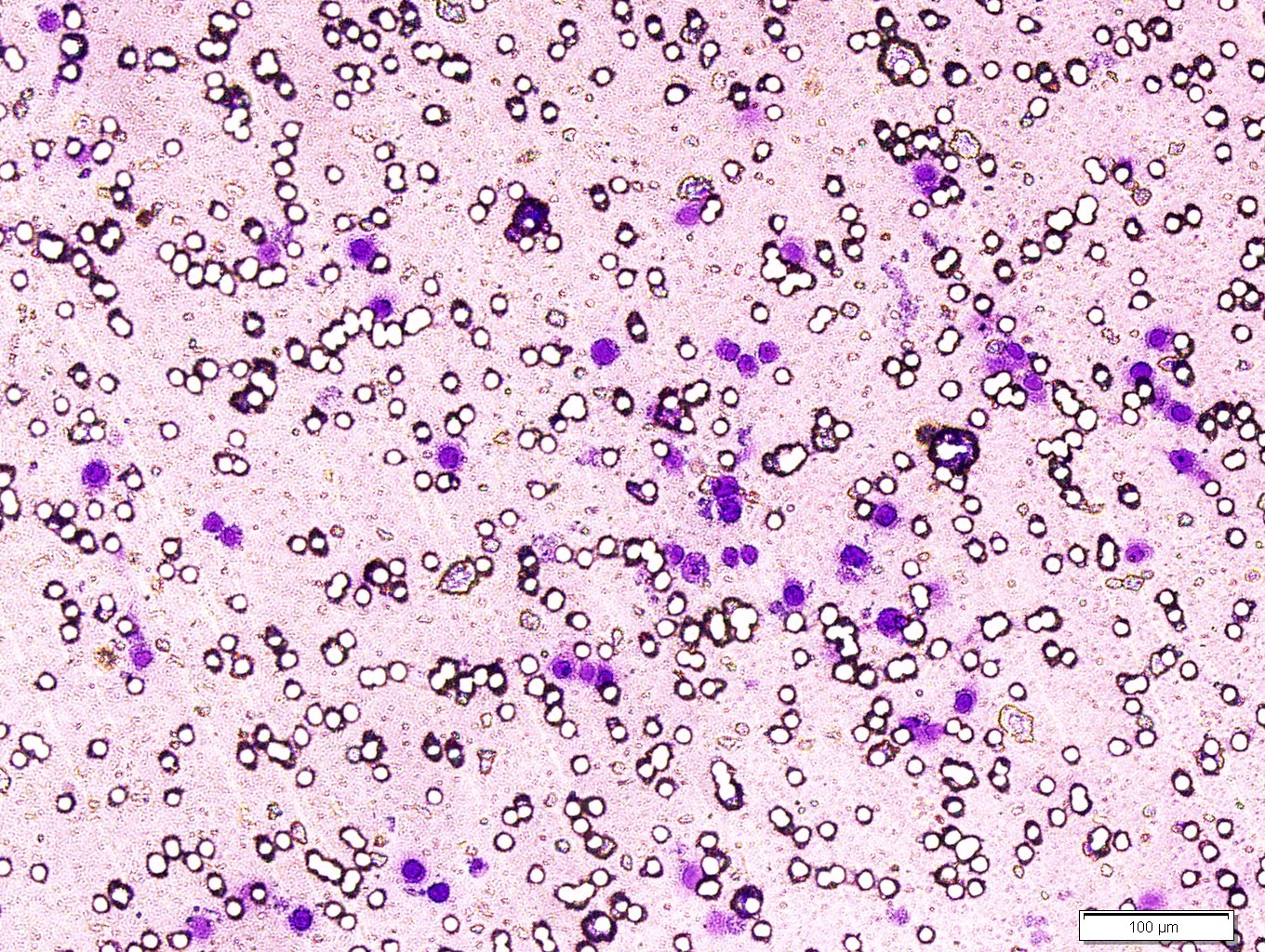

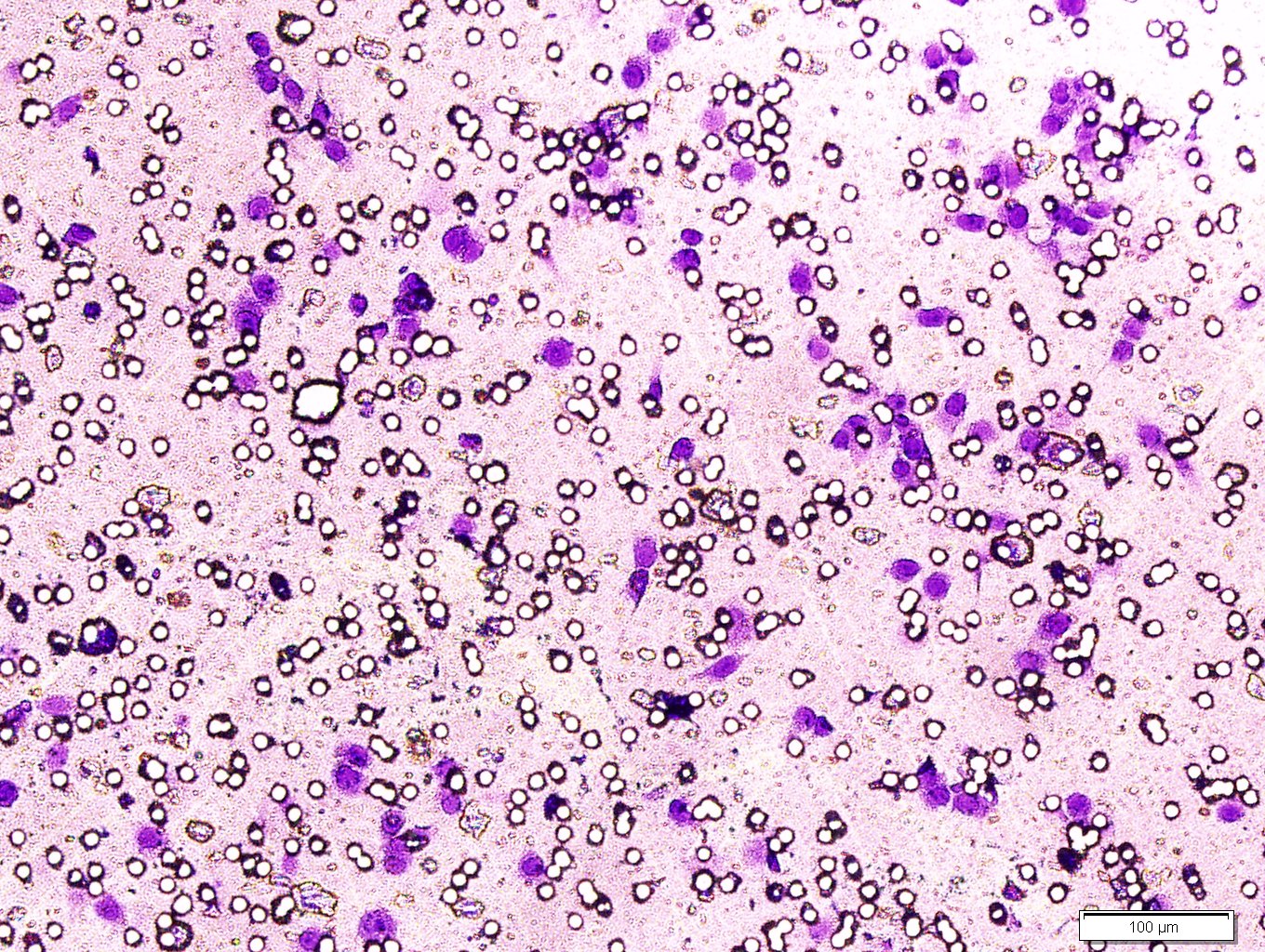

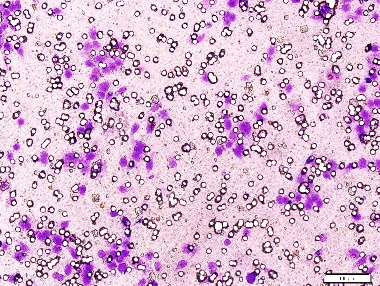


**pyk90-DEL** **N-BLR**

**pyk90-DEL2** **N-BLR**

**pyk90-DEL** **N-BLR**

**pyk90-DEL2** **N-BLR**

**Additional file 3: Fig. S18. Pyknon probe distribution across the genome. (A)** The probes used in a custom-made microarray for evaluating the transcription potential of selected pyknon-regions were widely distributed across the whole genome. **(B)** The probes’ design in relation with the position of the pyknon motif.

**A**

**B**

**Additional file 3: Fig. S19. Similarities and differences across tissues and tissue states.** Spearman correlation coefficient-based heat map matrix of normal tissues shows increased tissue specificity for pyknon regions (left) compared with miRNAs (right).

**Pyknon probes**

**miRNAs**

**Additional file 3: Fig. S20. Pyknons expression across disease states. (A)** An example of a pyknon signature formed solely of intergenic unique probes that can distinguish healthy colon samples from CRC samples. **(B)** A pyknon signature formed solely of intergenic unique probes that can distinguish CLL samples from healthy B-cell samples.

**A**

**B**

**B**

**Additional file 3: Fig. S21. Array data confirmation. (A)** Expression of pyk-reg-14 was higher in CLL than in B cells by both qRT-PCR and microarray (CLL with ZAP<10%: n=4 in qRT-PCR and n=7 in microarray; CLL with ZAP>60%: n=5 in qRT-PCR and n=8 in microarray; B cells: n=2 in both qRT-PCR and microarray). Data shown represent the mean ± SEM. The asterisk represents a statistically significant difference (***p*≤ 0.01). ZAP is ZAP70, the zeta-chain (TCR) associated protein kinase 70kDa, a clinically used prediction marker for CLL patient´s natural history. Expression of pyk-reg-10 **(B)** and pyk-reg-40 **(C)** in colorectal cancer was higher than in normal by microarray and qRT-PCR (colon cancer: n=80 in qRT-PCR and n=12 in microarray; normal colon: n=28 in qRT-PCR and n=4 in microarray). Data shown represent the mean ± SEM. The asterisk represents a statistically significant difference in the comparison between colon cancer and normal colon (**p*≤0.05; ***p*≤0.01).

**A**

**B**

**C**
